# Supplementary material for: Notifications on Pesticide Residues in the Rapid Alert System for Food and Feed (RASFF)
Source: Int J Environ Res Public Health. 2022 Jul 12;19(14):8525. doi: 10.3390/ijerph19148525 (PMC9324178; doi:10.3390/ijerph19148525)
Supplement: Supplementary file 1 [file ijerph-19-08525-s001.zip › ijerph-1777921-supplementary.pdf]

Supplementary Material

Notifications on Pesticide Residues in the Rapid Alert System for Food and Feed

Table S1. Number of notifications on particular pesticides in the RASFF in 1981–2020.

Pesticide	Number of Notifications	Pesticide	Number of Notifications
**pesticides analysed in detail**	**4061**	buprofezin	57
acephate	172	captan	8
acetamiprid	257	carbaryl	48
carbendazim	333	carbosulfan	7
carbofuran	184	chlorantraniliprole	4
chlorpyrifos	460	chlorate	27
dichlorvos	112	chlorbufam	2
dimethoate	326	chlorfenapyr	44
ethylene oxide	494	chlorfenvinphos	2
fipronil	186	chlorfluazuron	22
formetanate	203	chlormequat	99
methamidophos	176	chlorothalonil	20
methomyl	260	chlorpropham	7
monocrotophos	159	chlorpyrifos-methyl	63
omethoate	223	clofentezine	33
oxamyl	160	clothianidin	15
profenofos	143	copper	10
triazophos	213	cotinine	1
**all other pesticides**	**3952**	cyfluthrin	5
1,4-dichlorobenzene	8	cyhalothrin	12
2,4-D	1	cymoxanil	2
2-chloroethanol	7	cypermethrin	77
3-hydroxycarbofuran	2	cyproconazole	4
4-CPA	2	cyprodinil	10
abamectin	9	cyromazine	4
acrinathrin	4	DDT	29
aldicarb	5	deltamethrin	17
aldrin	2	demeton-S-methyl	1
allethrin	1	diafenthiuron	46
amitraz	77	diazinon	37
anthraquinone	77	dichlofluanide	1
azaconazole	1	dicloran	3
azinphos-methyl	19	dicofol	33
azoxystrobin	27	dicrotophos	19
bifenthrin	38	didecyldimethylammonium chloride (DDAC)	22
biphenyl	16		
bitertanol	2	dieldrin	7
boscalid	19	diethion	21
bromide	16	difenoconazole	16
bromopropylate	10	diflubenzuron	2
bromuconazole	1	dimefuron	1
bupirimate	1	dimethomorph	18

Pesticide	Number of notifications	Pesticide	Number of notifications
diniconazole	4	fonofos	2
dinotefuran	47	formothion	1
diphenylamine	7	fosthiazate	43
disulfoton	1	glyphosate	30
dithiocarbamates	67	haloxyfop	2
dodine	5	HCH	29
E 324 - ethoxyquin	3	heptachlor	7
emamectin	5	heptenophos	1
endosulfan	79	hexachlorobenzene	12
endrin	1	hexachlorohexane	2
EPN	26	hexaconazole	55
esfenvalerate	20	hexaflumuron	9
ethephon	93	hydrogen phosphide	3
ethion	92	hymexazol	1
ethofumesate	1	imazalil	61
ethoprofos	3	imidacloprid	88
ethylene dibromide	1	indoxacarb	21
etofenprox	3	iprobenfos	3
etoxazole	4	iprodione	40
famoxadone	6	iprovalicarb	2
fenamidone	1	isocarbophos	13
fenamiphos	21	isofenphos-methyl	43
fenarimol	3	isoprocarb	8
fenazaquin	2	isoprothiolane	9
fenbutatin oxide	7	kresoxim-methyl	6
fenhexamid	7	lambda-cyhalothrin	72
fenitrothion	29	linuron	1
fenobucarb	4	lufenuron	18
fenpropathrin	39	magnesium phosphide	1
fenpropidin	2	malathion	75
fenpyroximate	1	mandipropamid	3
fenthion	16	matrine	6
fenvalerate	53	mepiquat	3
flazasulfuron	1	mepronil	1
flonicamid	27	metalaxyl	27
fluazifop-p	11	metaldehyde	1
fluazifop-P-butyl	6	metamitron	2
fluazinam	1	methidathion	30
flubendiamide	11	methiocarb	23
fludioxonil	6	methoxychlor	3
flufenoxuron	3	methoxyfenozide	9
fluopicolide	2	methyl bromide	5
fluopyram	4	metrafenone	4
fluoxastrobin	1	mirex	1
flupyradifuron	1	myclobutanil	12
flusilazole	22	N,N-diethyl-meta-toluamide (DEET)	5
flutriafol	4		
folpet	10	naphthalene	2

Pesticide	Number of notifications	Pesticide	Number of notifications
nicotine	23	pyrazophos	1
nitrofen	17	pyridaben	69
novaluron	1	pyridalyl	4
nuarimol	4	pyrimethanil	11
organophosphate pesticides	3	quinalphos	10
orthophenylphenol	3	quinoxyfen	1
oxadixyl	1	quintozene	2
oxydemeton-methyl	9	raticide	5
paclobutrazol	1	roxymidone	1
paraoxon-methyl	1	slug pellets	2
parathion	4	spinosad	3
parathion-methyl	22	spiromesifen	7
penconazole	8	spirotetramat	2
pencycuron	3	sulfotep	3
pentachlorophenol	12	sulphur	3
permethrin	43	syprodiinil	1
pesticide residues (not specified)	34	tau-fluvalinate	10
		tebuconazole	47
phenmedipham	1	tebufenozide	1
phentachloroaniline	1	tebufenpyrad	7
phenthoate	17	tecnazene	1
phorate	5	teflubenzuron	4
phosalone	13	terbucarb	1
phosmet	7	tetraconazole	7
phosphamidone	1	tetradifon	44
phoxim	1	tetramethrin	9
picoxystrobin	1	thiabendazole	21
piperonylbutoxide	4	thiacloprid	8
pirimicarb	6	thiamethoxam	35
pirimiphos-methyl	38	thiodicarb	10
prochloraz	76	thiophanate-methyl	41
procymidone	96	tolfenpyrad	63
promecarb	3	triadimefon	8
prometryn	5	triadimenol	13
propamocarb	9	trichlorfon	10
propargite	94	tricyclazole	61
propiconazole	31	trifloxystrobin	11
propoxur	4	trifluralin	17
proquinazid	1	triforine	1
prothiofos	15	vinclozolin	1
pymetrozine	2	**Total**	**8013**
pyraclostrobin	17		

Table S2. Short names of some values of the variables: product category, notification basis, distribution status and action taken.

Short Name	Original Name
**Product Category**	
cereals	cereals and bakery products
cocoa, coffee, tea	cocoa and cocoa preparations, coffee and tea
crustaceans	crustaceans and products thereof
dietetic foods…	dietetic foods, food supplements, fortified foods
eggs	eggs and egg products
fats, oils	fats and oils
feed for food	feed for food-producing animals
fish	fish and fish products
food additives	food additives and flavourings
food materials	food contact materials
fruits, vegetables	fruits and vegetables
herbs, spices	herbs and spices
honey	honey and royal jelly
meat	meat and meat products (other than poultry)
milk	milk and milk products
beverages	non-alcoholic beverages
nuts	nuts, nut products and seeds
other food product	other food product / mixed
poultry meat	poultry meat and poultry meat products
prepared dishes	prepared dishes and snacks
soups, broths…	soups, broths, sauces and condiments
**notification basis**	
border control – detained	border control - consignment detained
border control – released	border control - consignment released
border control – customs	border control - consignment under customs
official control / RASFF	official control following RASFF notification
official control	official control on the market
**distribution status**	
distribution (possible)	distribution on the market (possible)
distribution restricted	distribution restricted to notifying country
distr. to non-member countries	distribution to non-member countries only
distribution to other countries	distribution to other member countries
information not available	information on distribution not (yet) available
no distr. from notif. country	no distribution from notifying country
no distr. to other countries	no distribution to other member countries
product no longer on market	product (presumably) no longer on the market
product consumed	product already consumed
product forw. to destination	product forwarded to destination
product not placed on market	product not (yet) placed on the market
**action taken**	
destination identified	destination of the product identified
import not authorised	import not authorised
phys./chem. treatment	physical/chemical treatment
placed under customs	placed under customs seals
product consumed	product already consumed
product recall/withdrawal	product recall or withdrawal
sales ban	prohibition to trade - sales ban
public warning – press	public warning - press release
product passed the MDD	product passed the Minimal Durability Date
use for other purpose	use for other purpose than food/feed
withdrawal from recipient	withdrawal from recipient(s)
withdrawal from market	withdrawal from the market

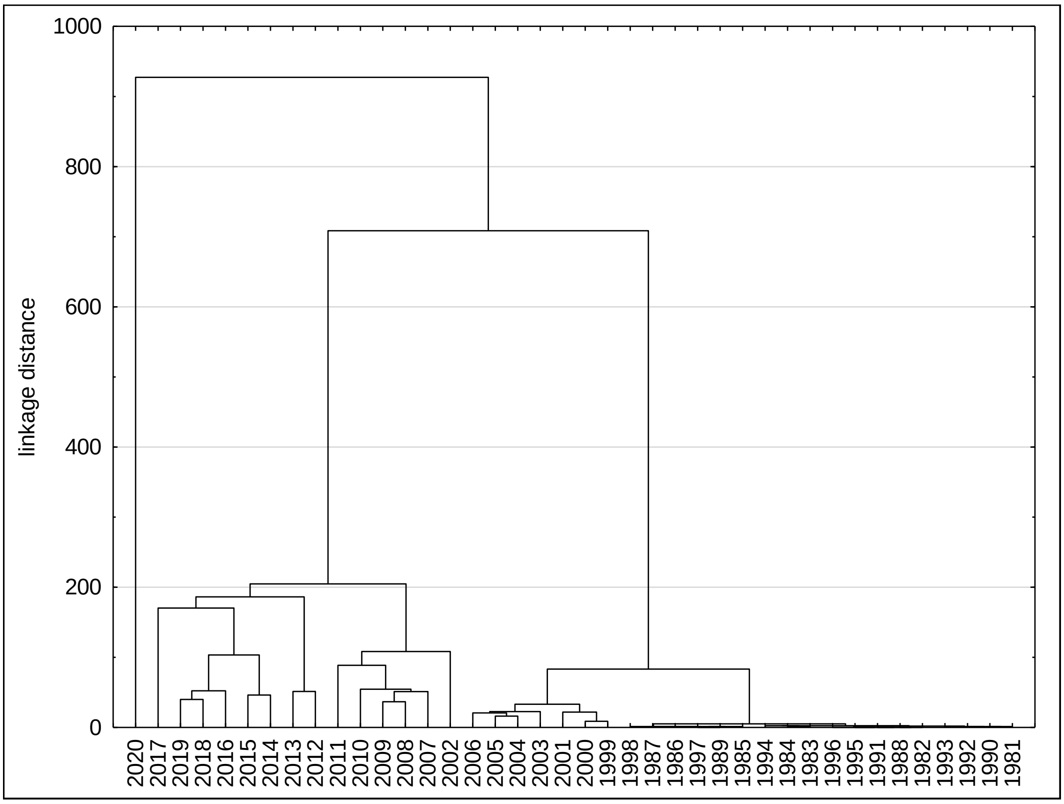

Figure S1a. Results of joining cluster analysis for years

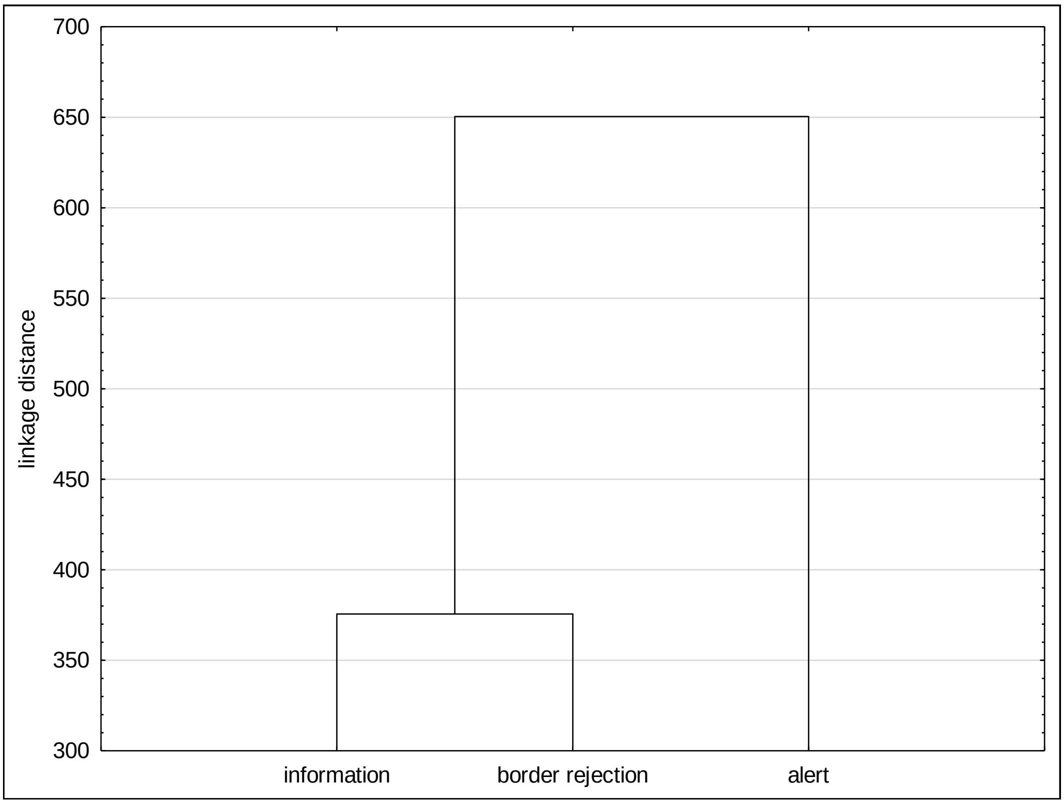

Figure S1b. Results of joining cluster analysis for notification type

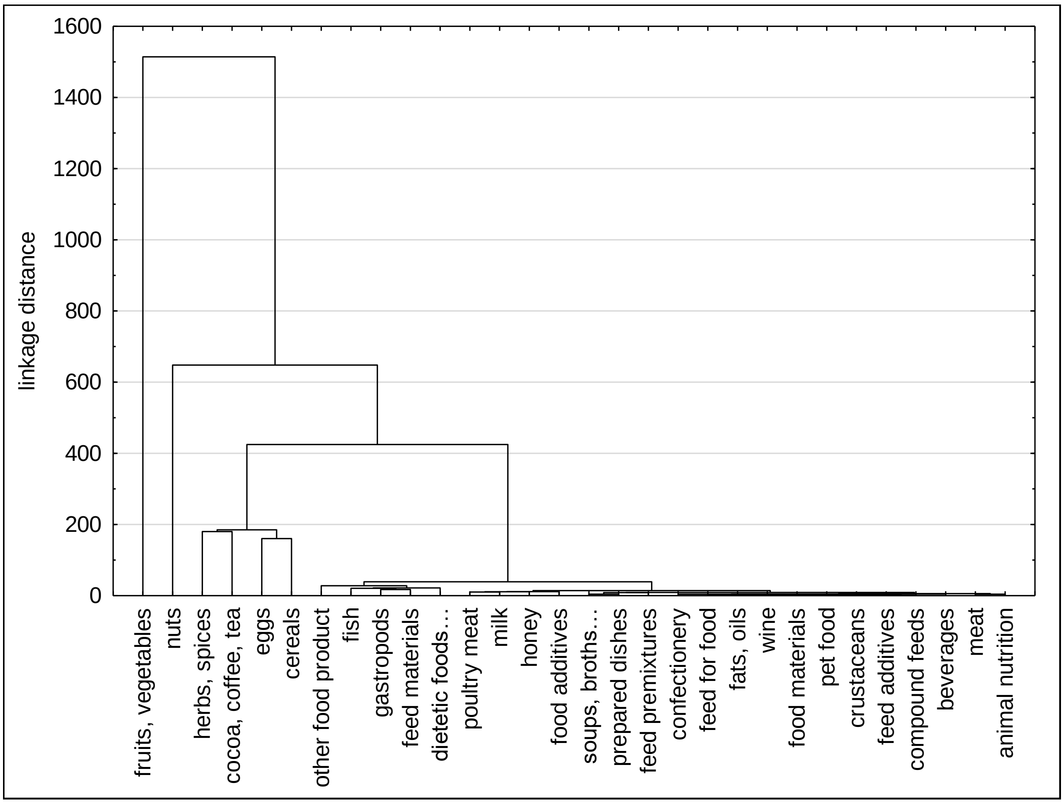

Figure S1c. Results of joining cluster analysis for product categories

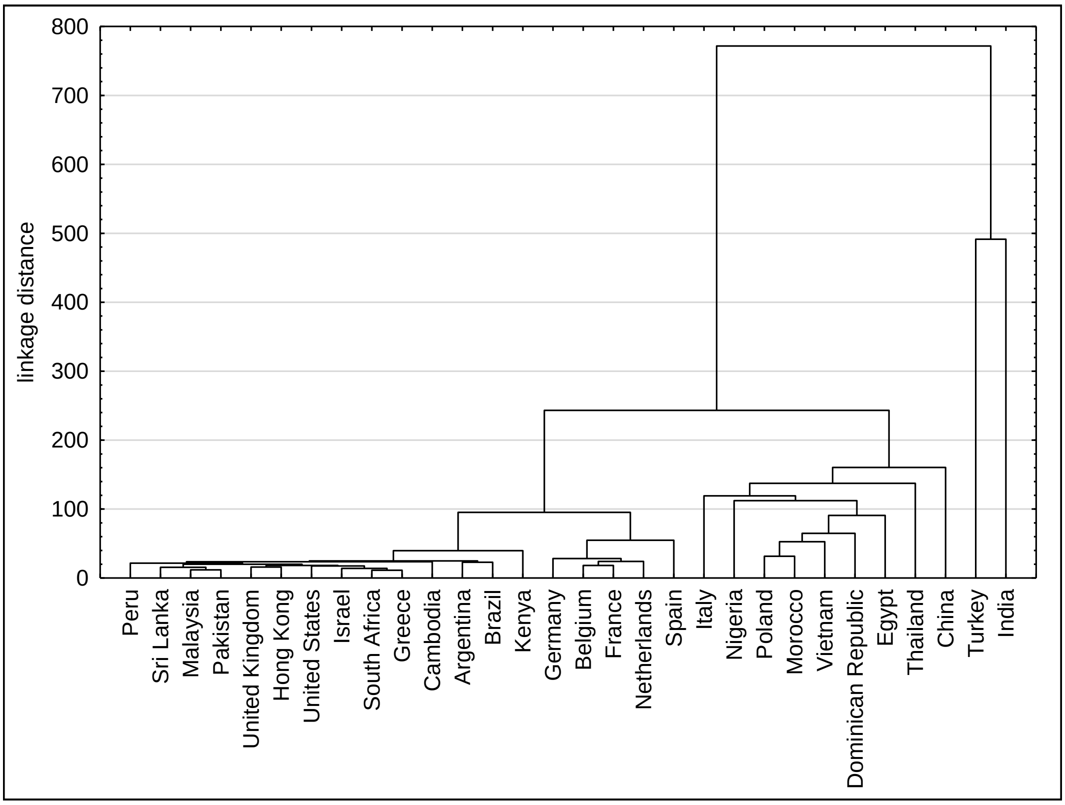

Figure S1d. Results of joining cluster analysis for origin countries

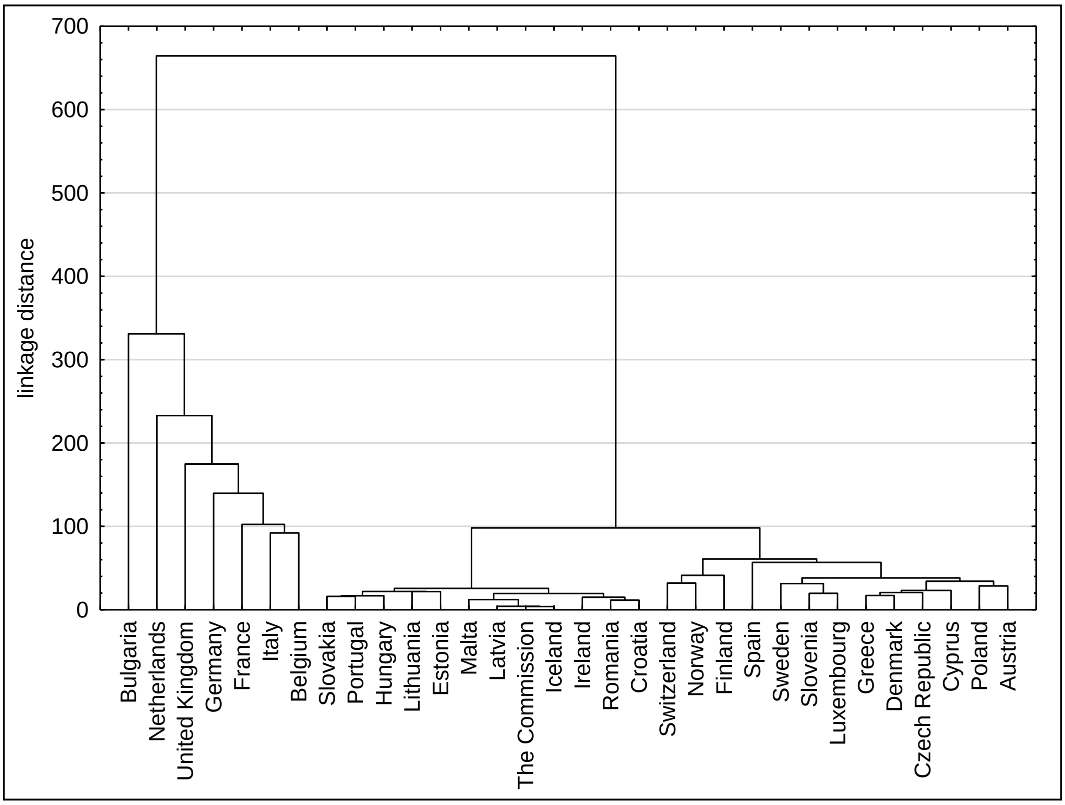

Figure S1e. Results of joining cluster analysis for notifying countries

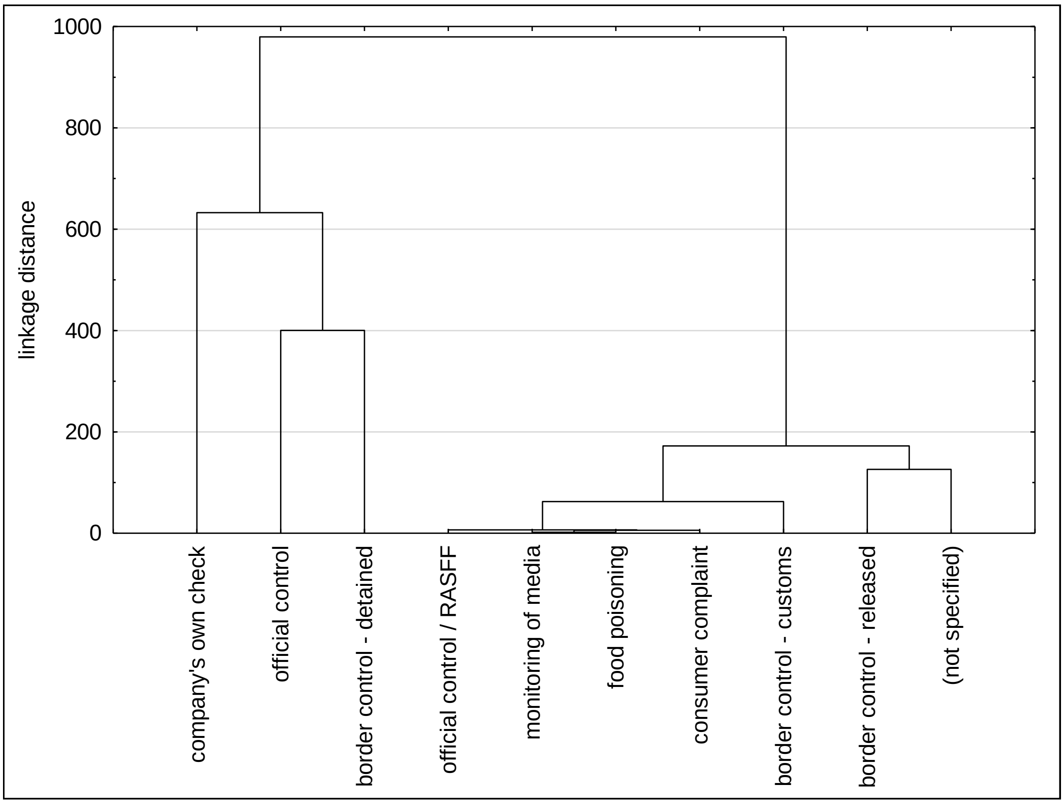

Figure S1f. Results of joining cluster analysis for notification basis

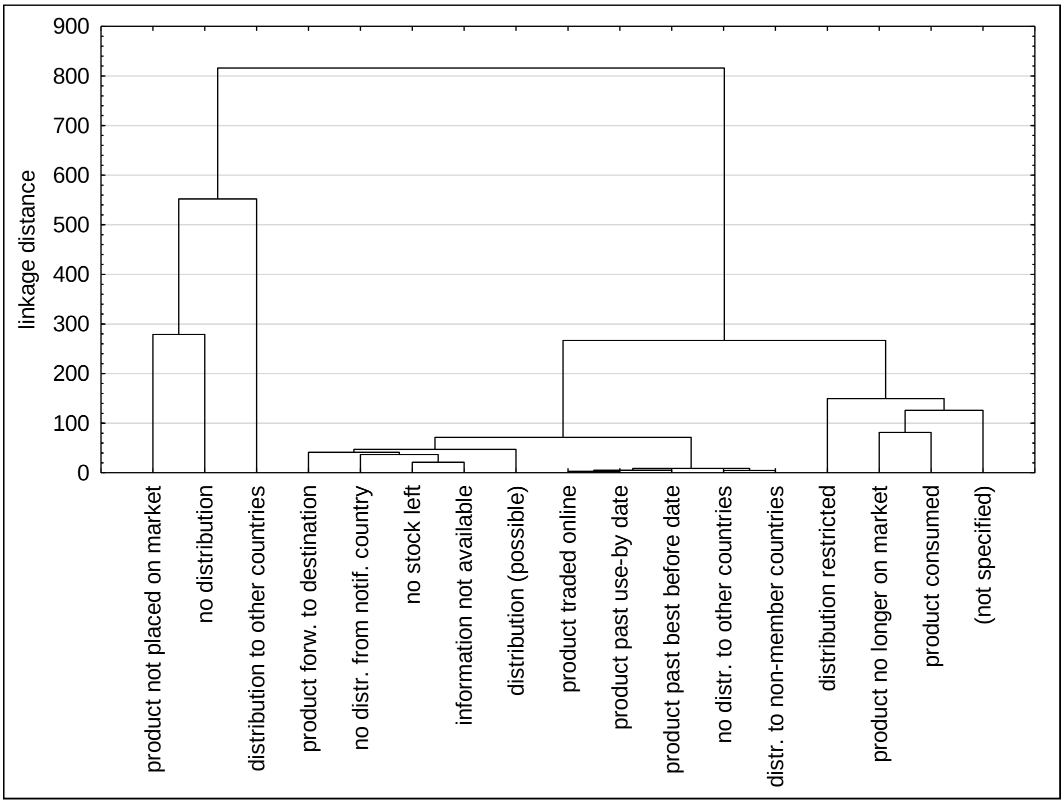

Figure S1g. Results of joining cluster analysis for distribution status

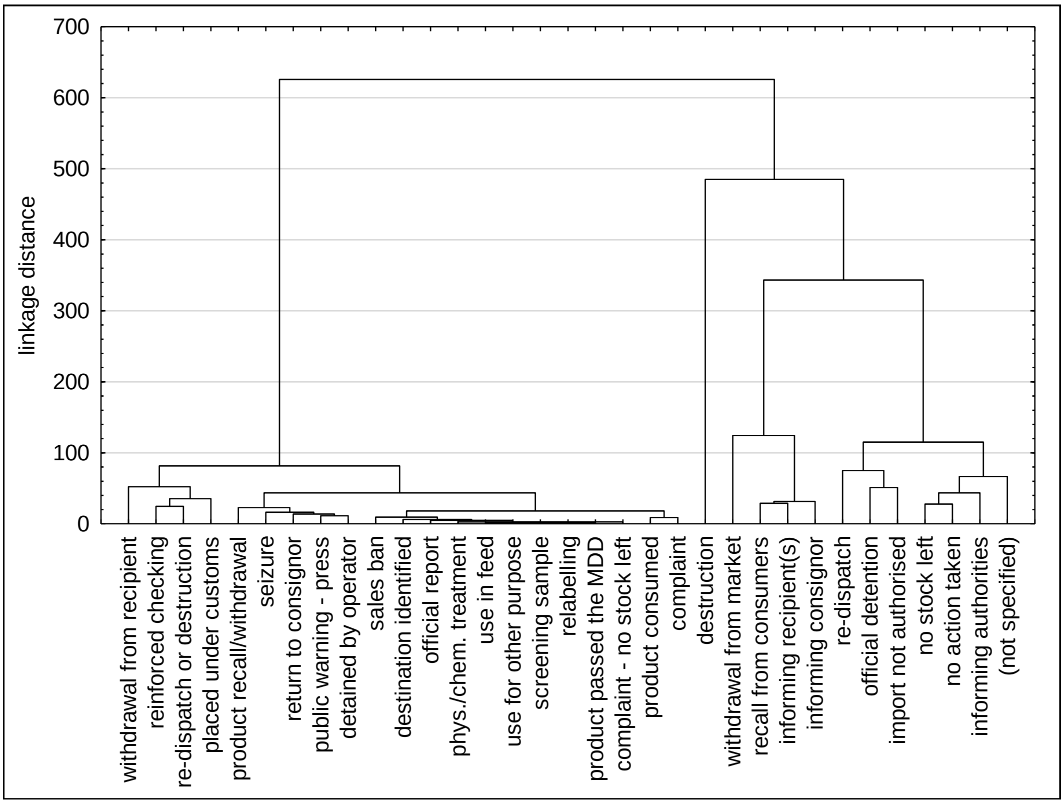

Figure S1h. Results of joining cluster analysis for action taken

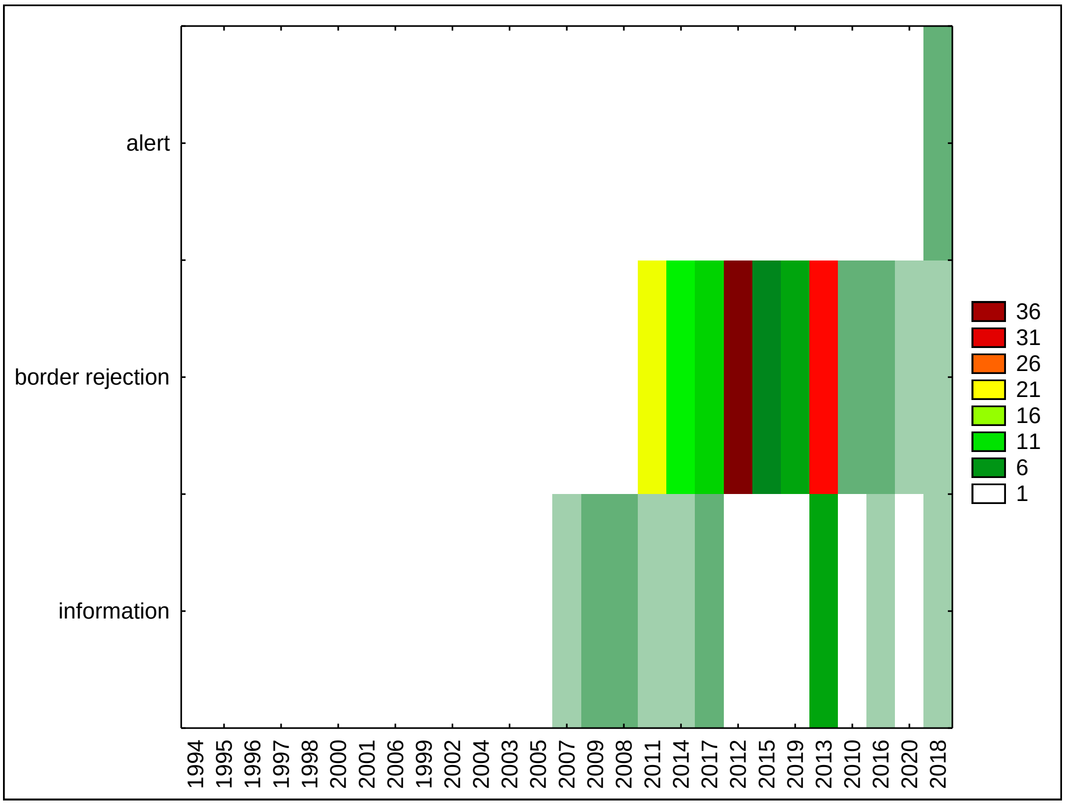

Figure S2a. Results of two-way joining cluster analysis for acephate (notification type)

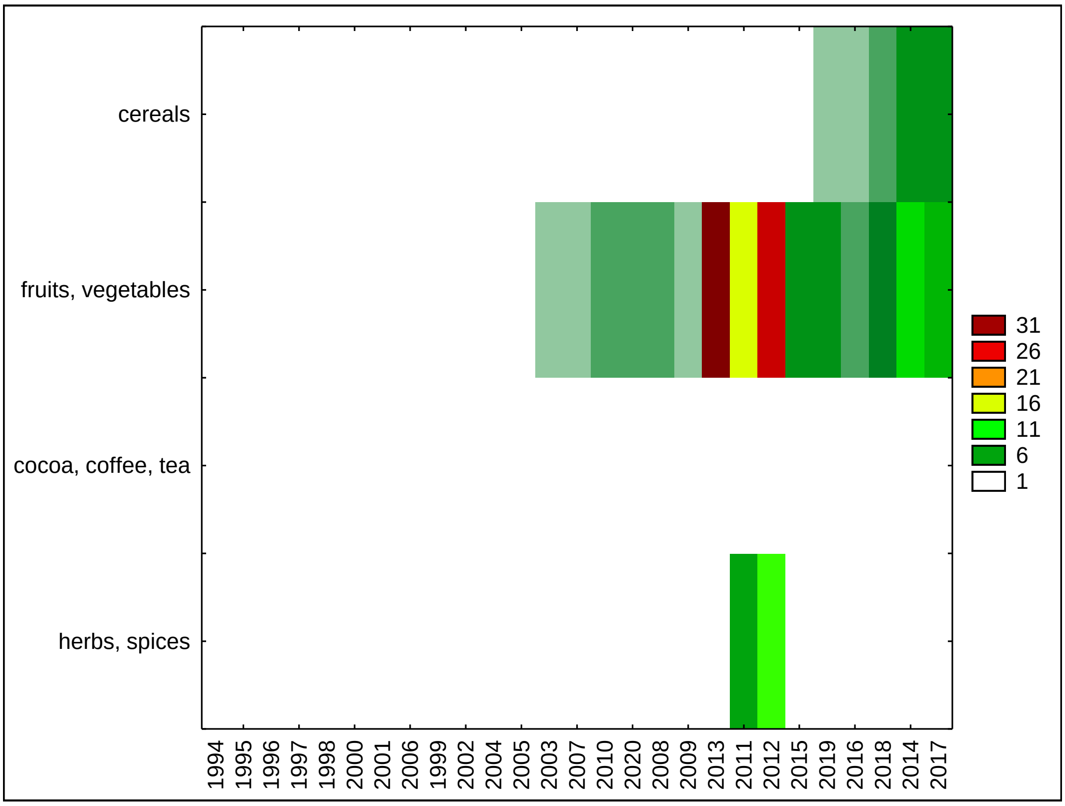

Figure S2b. Results of two-way joining cluster analysis for acephate (product category)

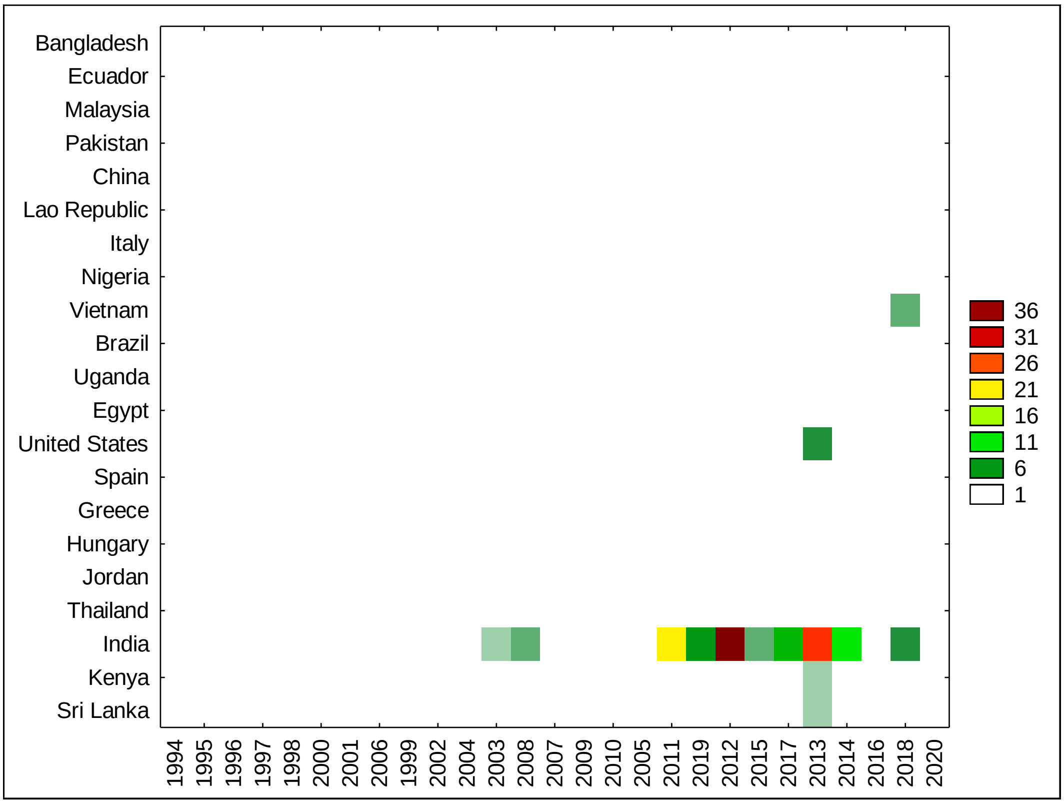

Figure S2c. Results of two-way joining cluster analysis for acephate (origin country)

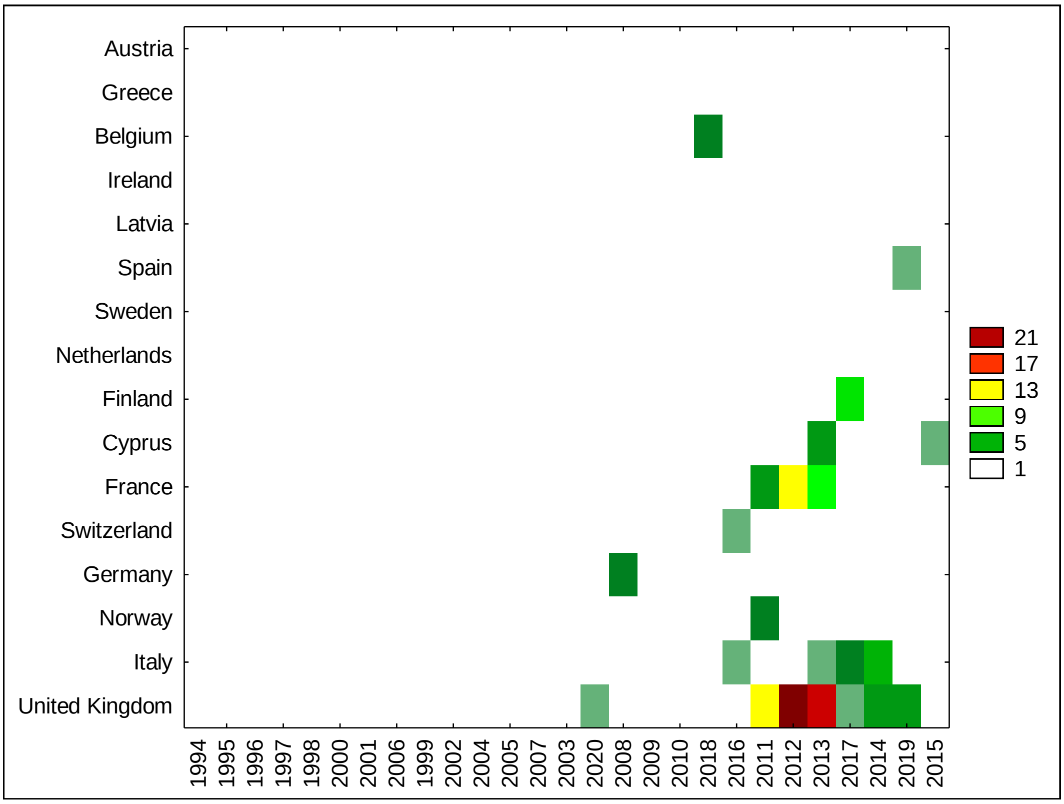

Figure S2d. Results of two-way joining cluster analysis for acephate (notifying country)

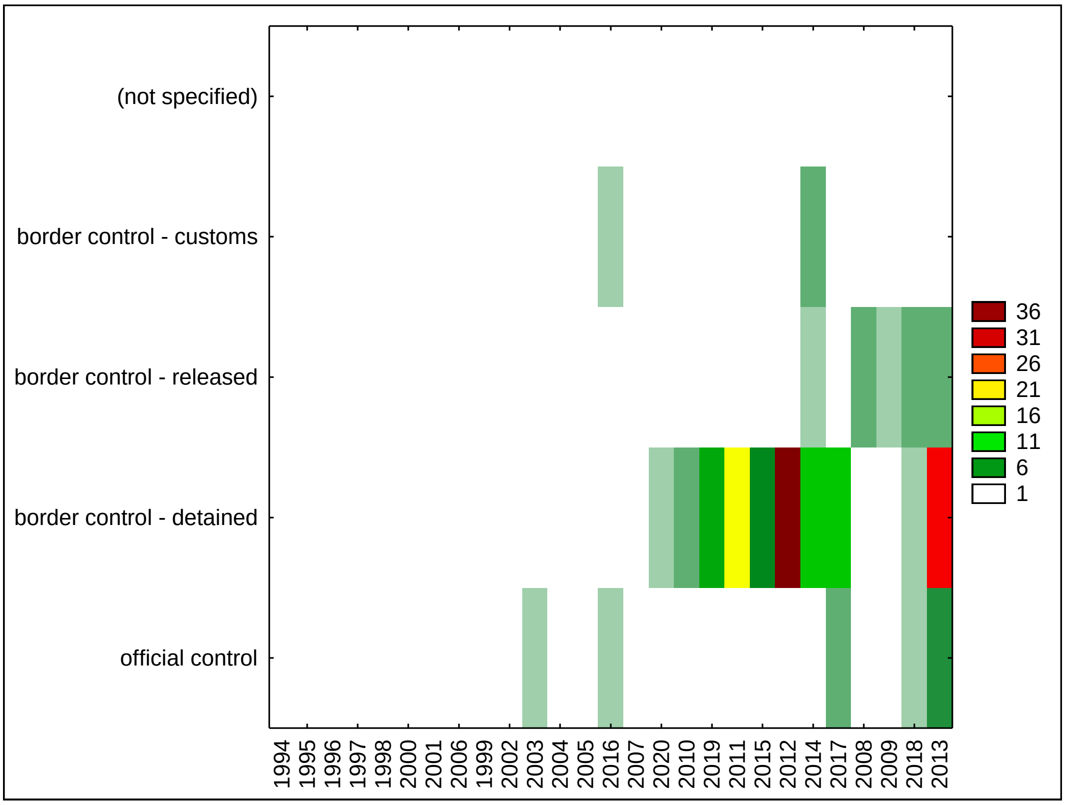

Figure S2e. Results of two-way joining cluster analysis for acephate (notification basis)

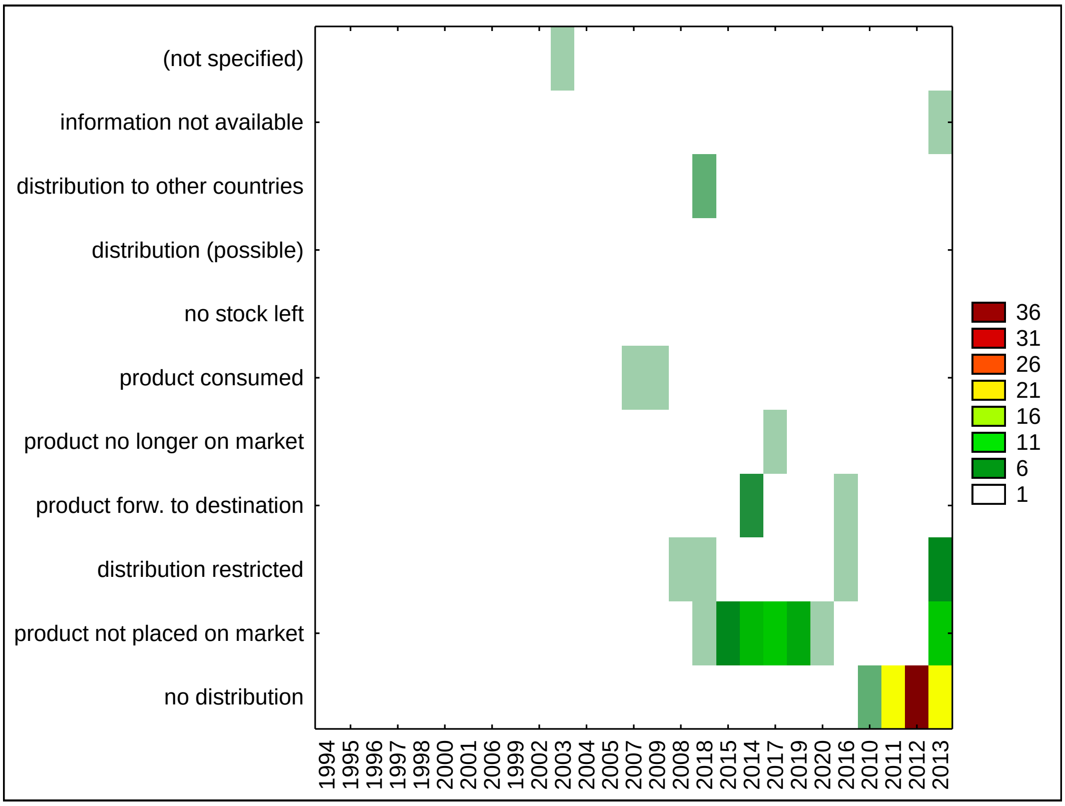

Figure S2f. Results of two-way joining cluster analysis for acephate (distribution status)

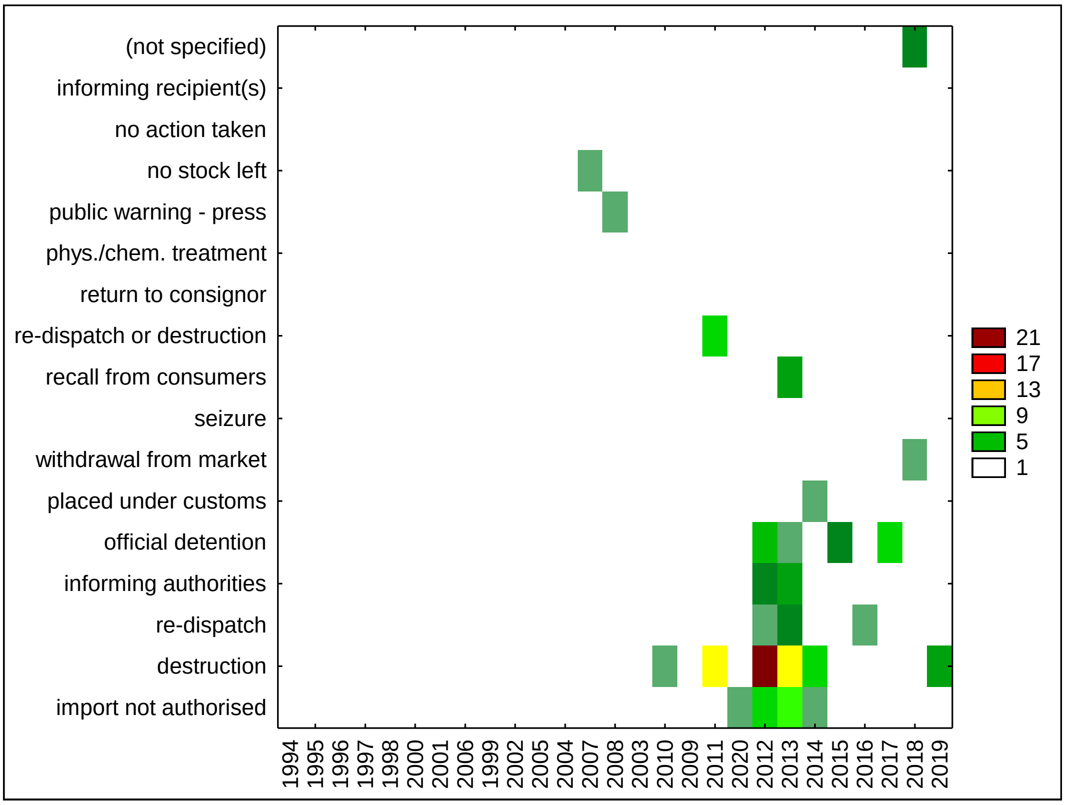

Figure S2g. Results of two-way joining cluster analysis for acephate (action taken)

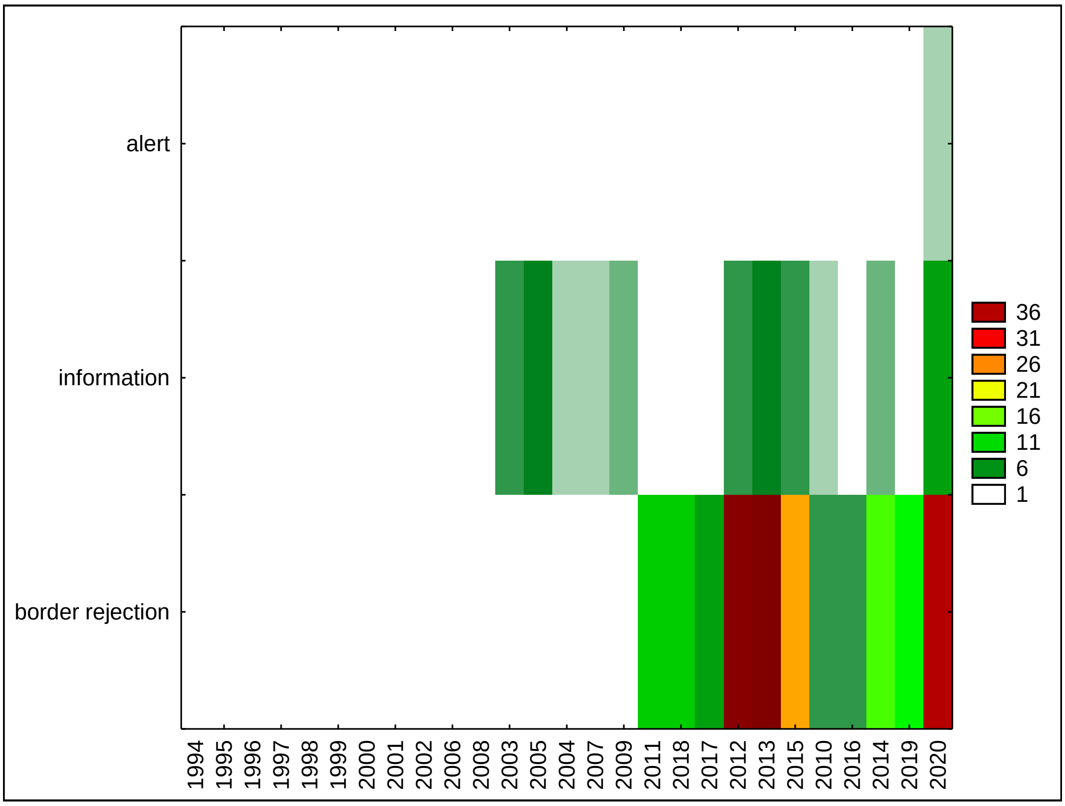

Figure S3a. Results of two-way joining cluster analysis for acetamiprid (notification type)

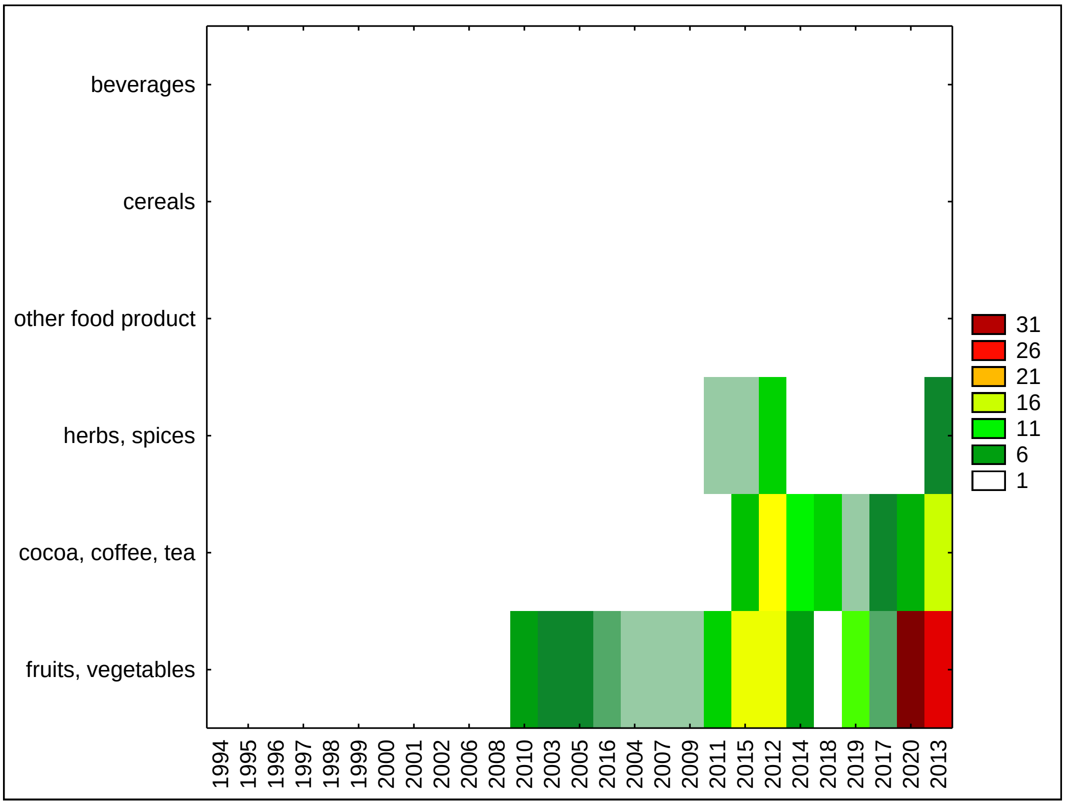

Figure S3b. Results of two-way joining cluster analysis for acetamiprid (product category)

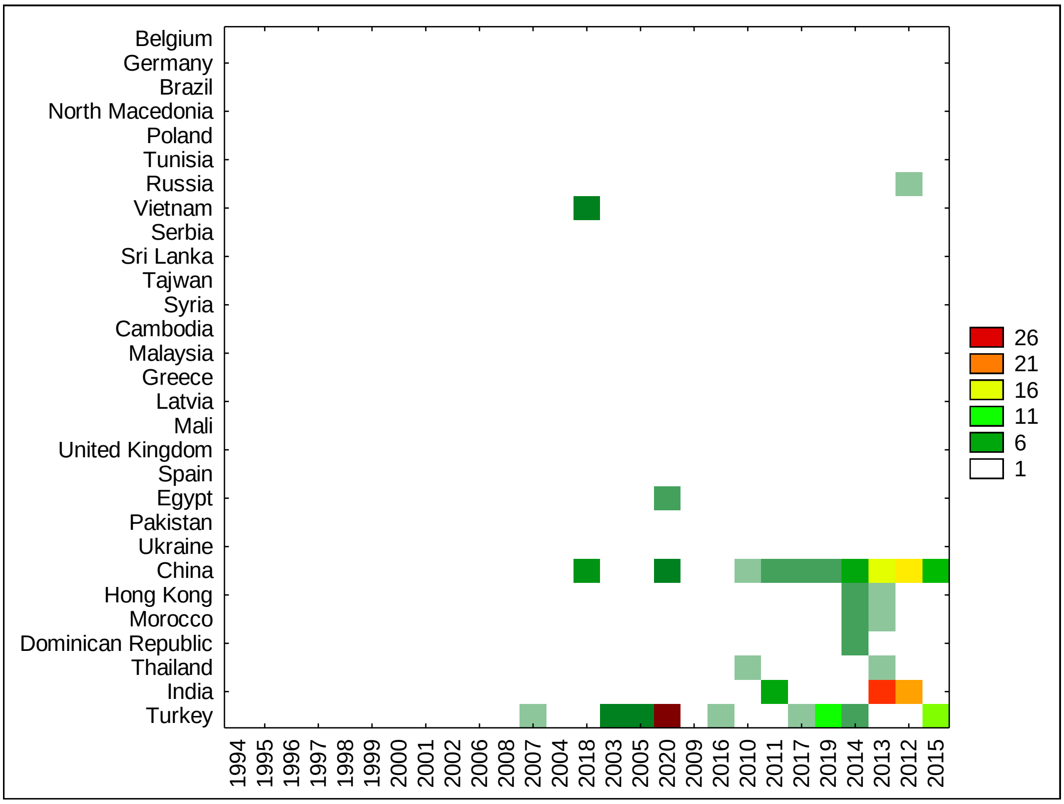

Figure S3c. Results of two-way joining cluster analysis for acetamiprid (origin country)

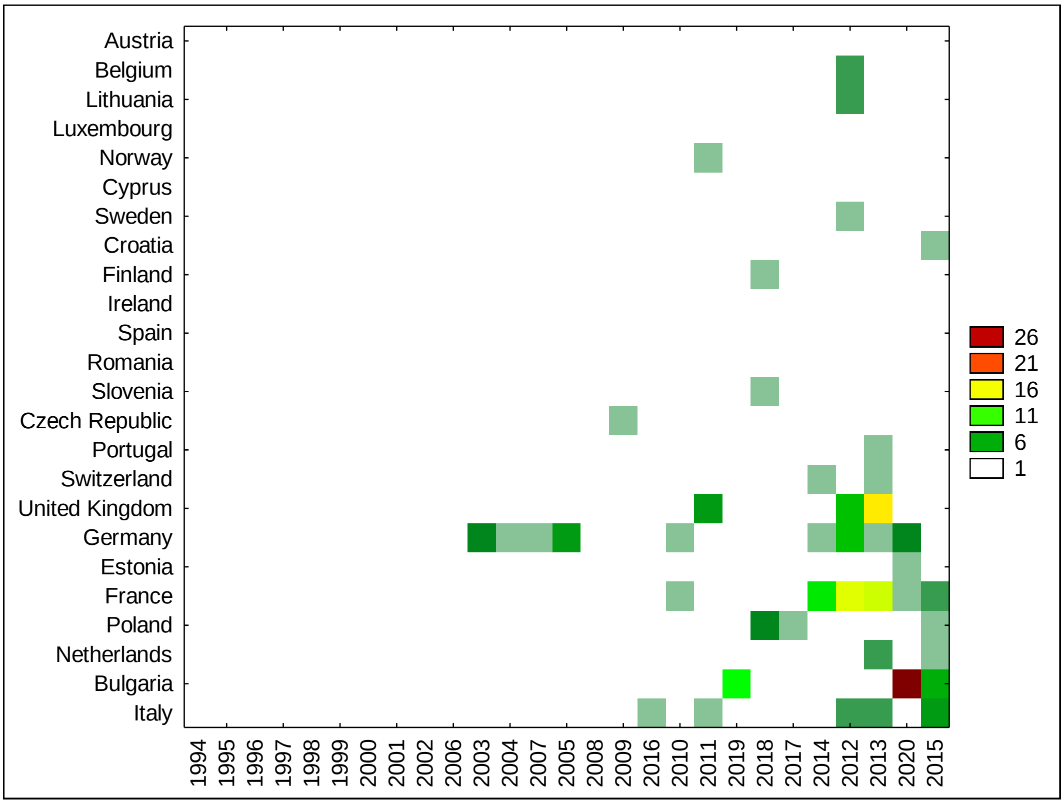

Figure S3d. Results of two-way joining cluster analysis for acetamiprid (notifying country)

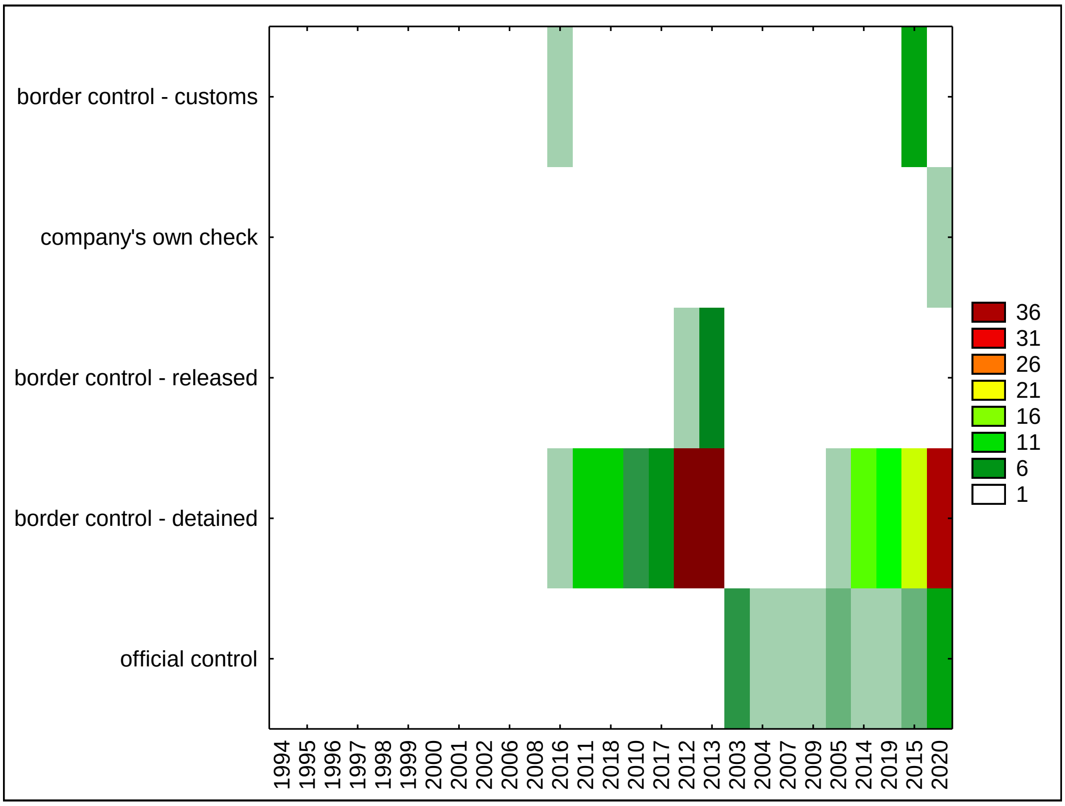

Figure S3e. Results of two-way joining cluster analysis for acetamiprid (notification basis)

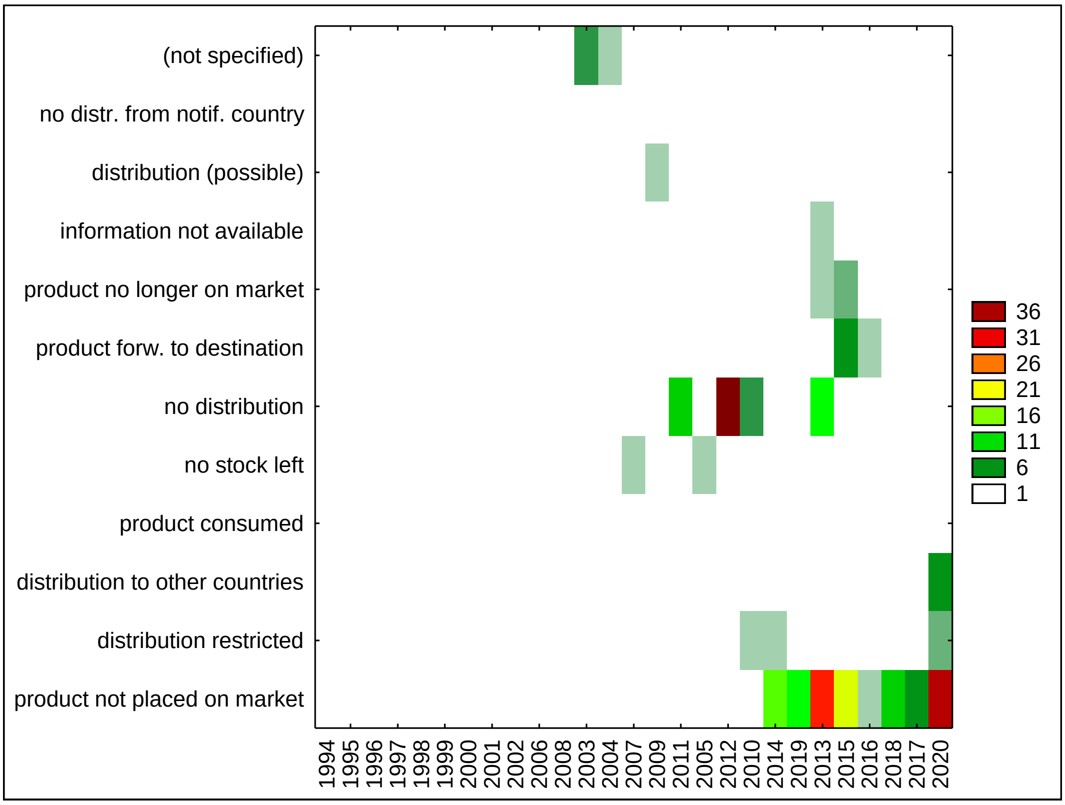

Figure S3f. Results of two-way joining cluster analysis for acetamiprid (distribution status)

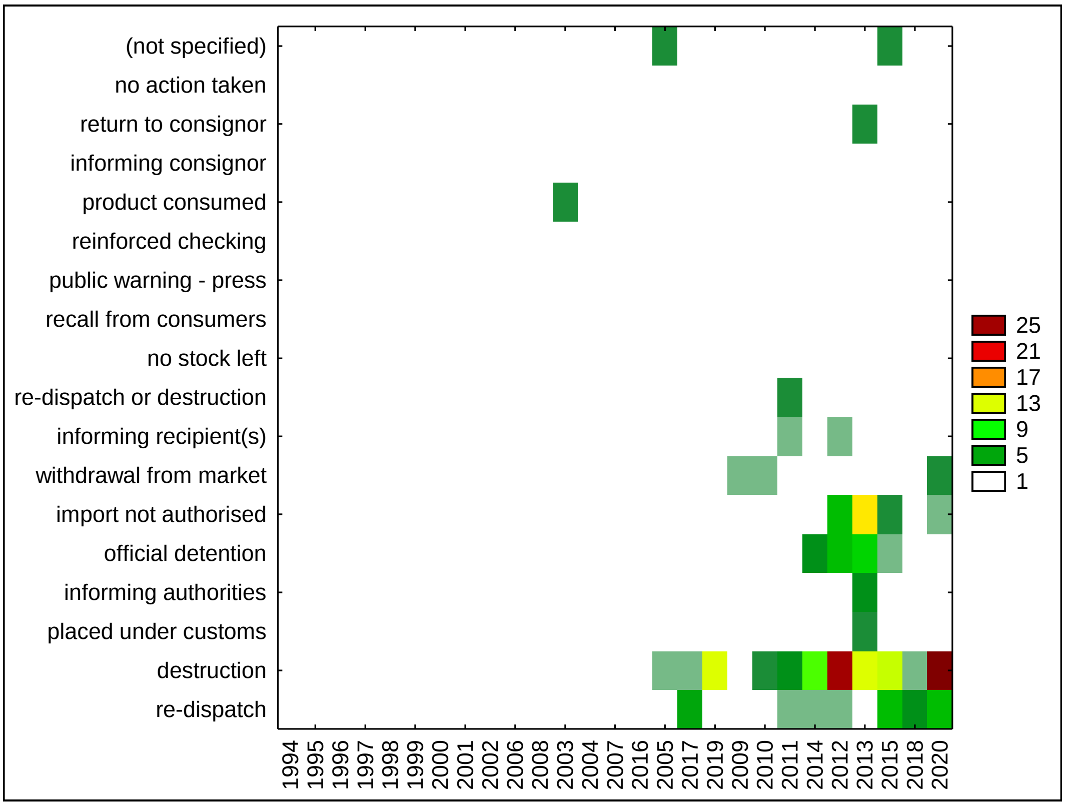

Figure S3g. Results of two-way joining cluster analysis for acetamiprid (action taken)

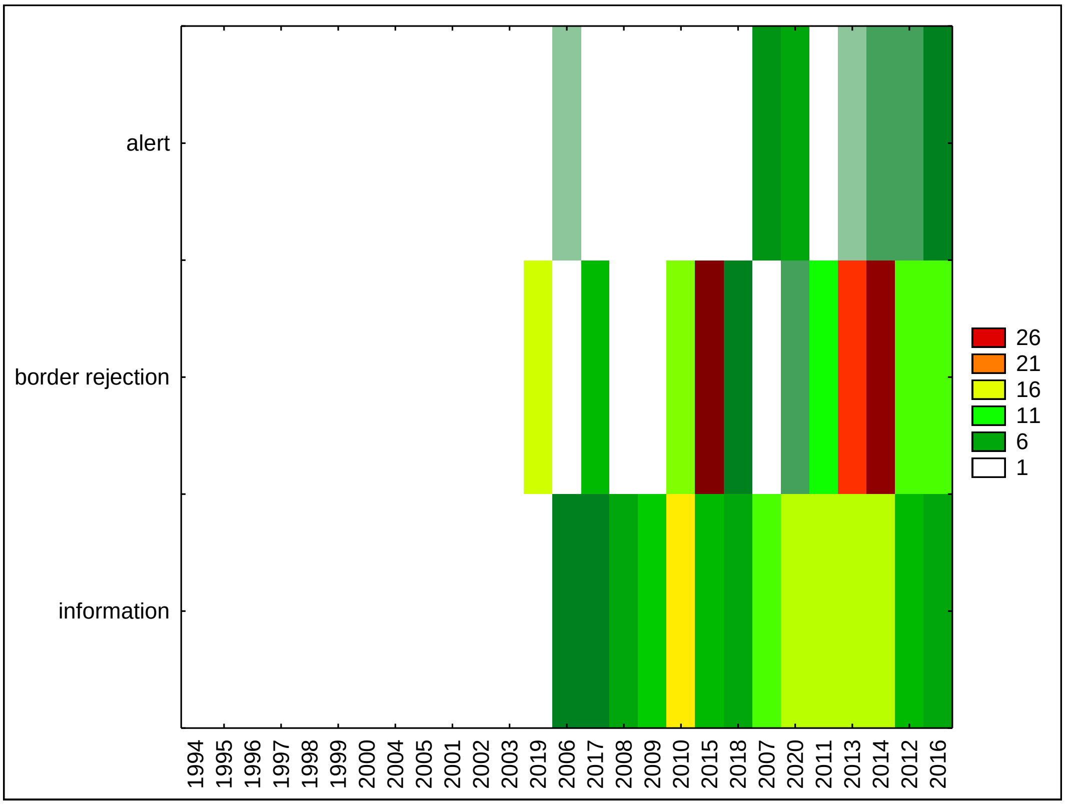

Figure S4a. Results of two-way joining cluster analysis for carbendazim (notification type)

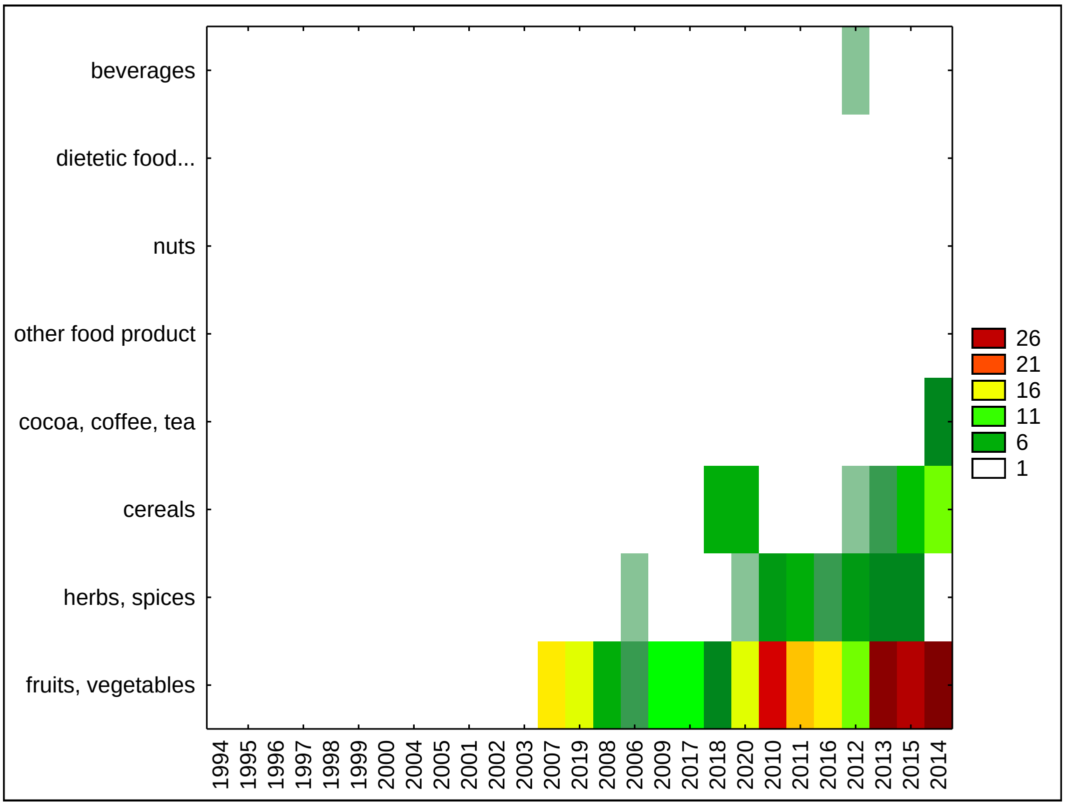

Figure S4b. Results of two-way joining cluster analysis for carbendazim (product category)

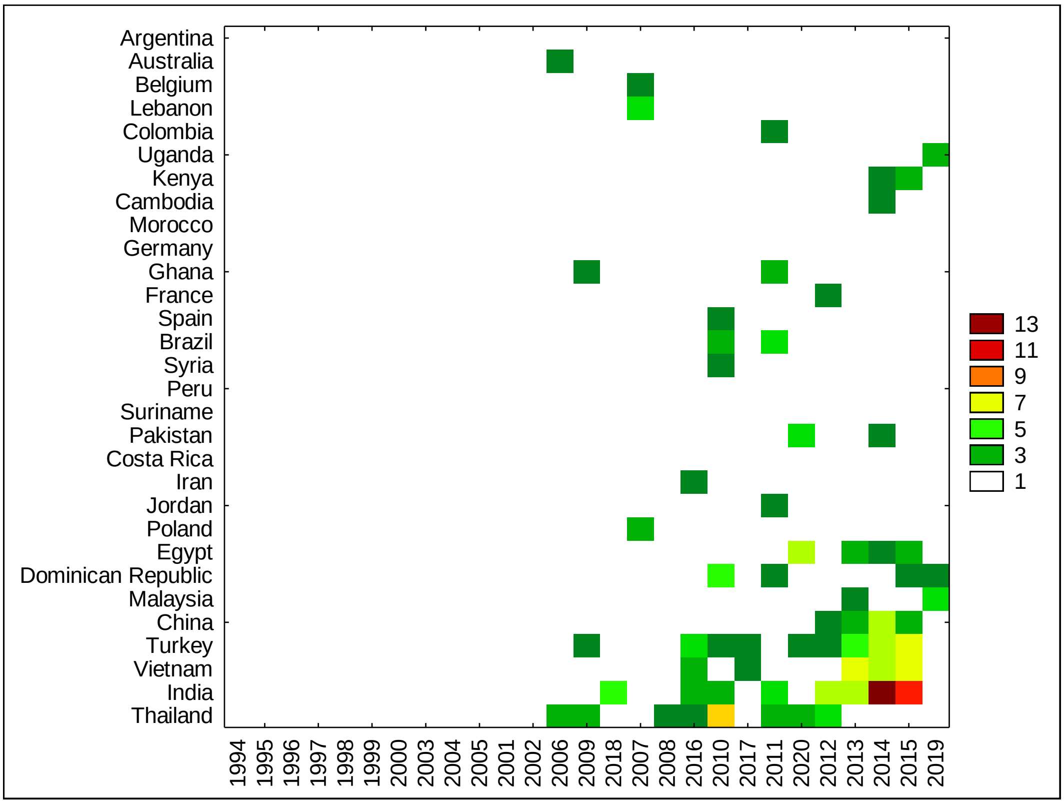

Figure S4c. Results of two-way joining cluster analysis for carbendazim (origin country)

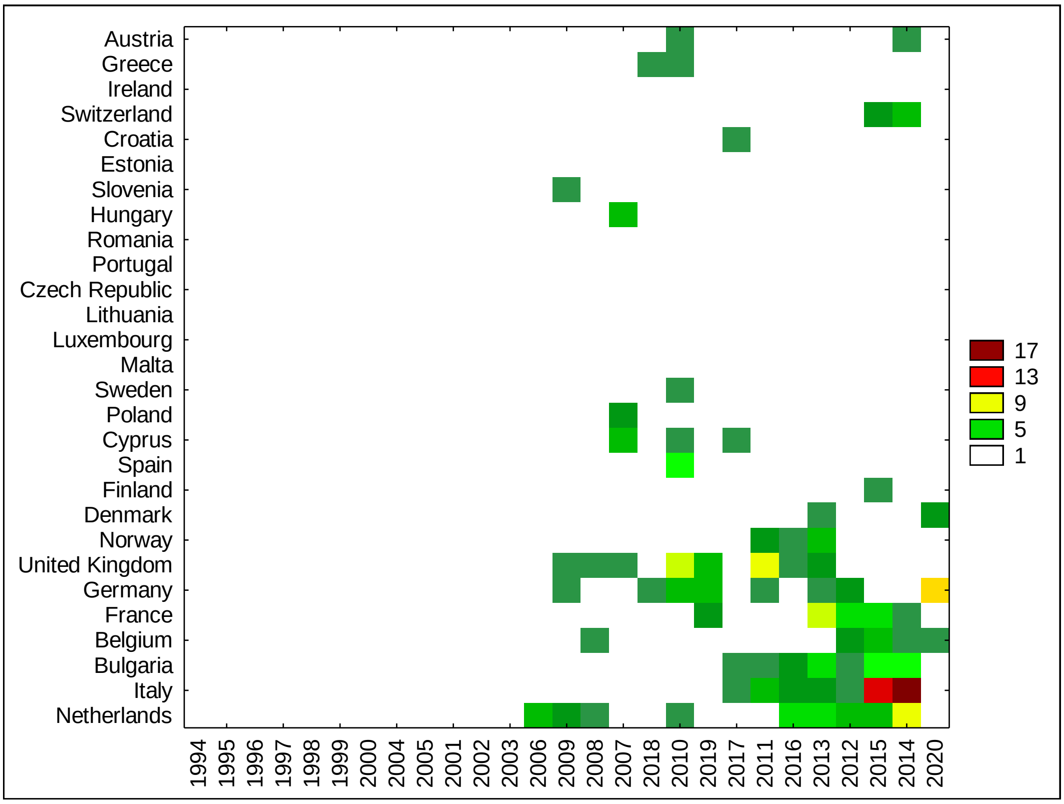

Figure S4d. Results of two-way joining cluster analysis for carbendazim (notifying country)

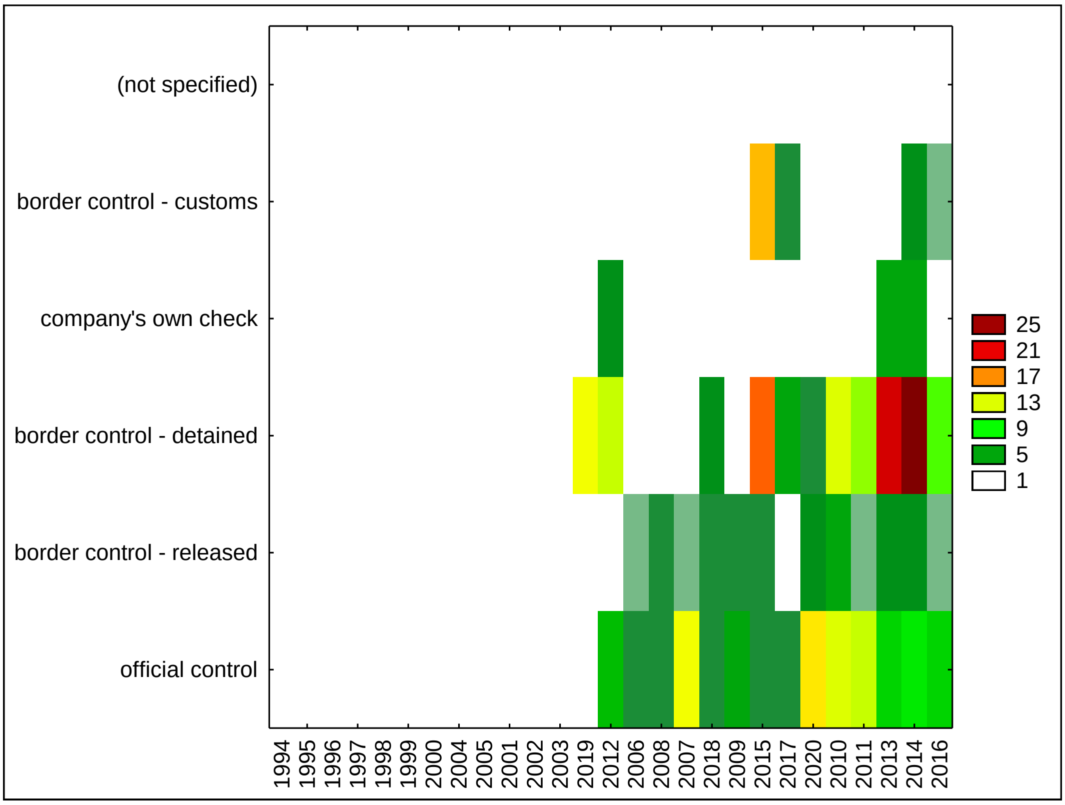

Figure S4e. Results of two-way joining cluster analysis for carbendazim (notification basis)

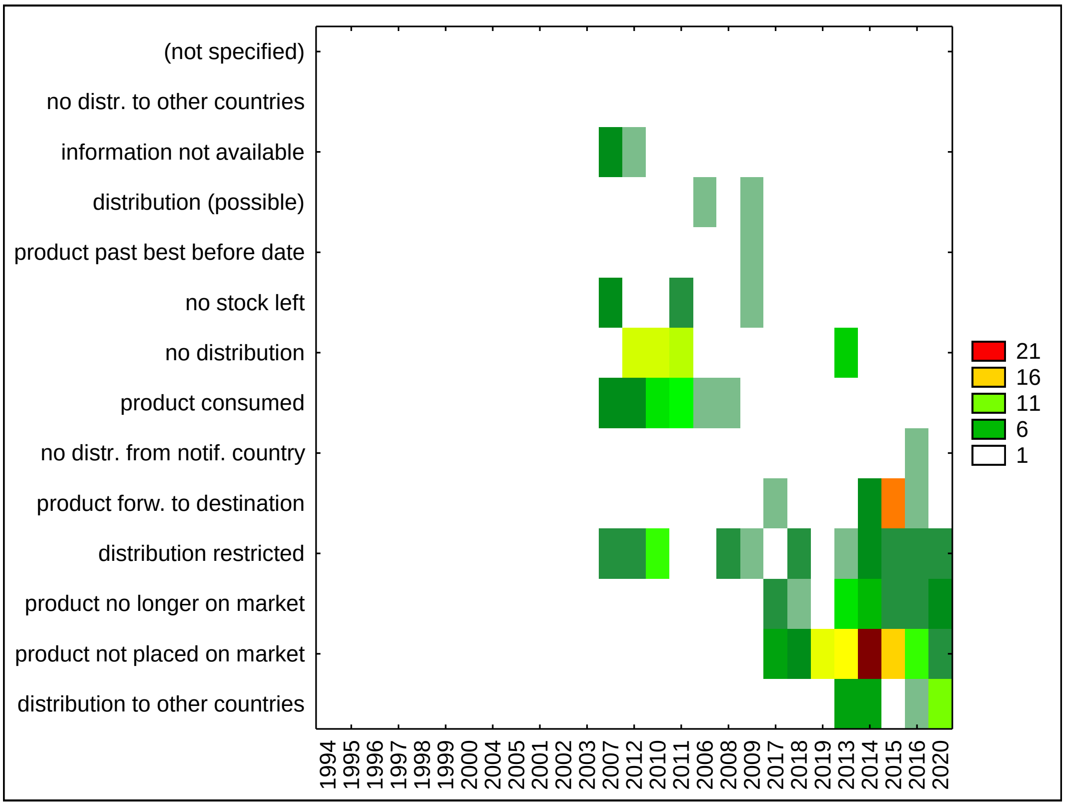

Figure S4f. Results of two-way joining cluster analysis for carbendazim (distribution status)

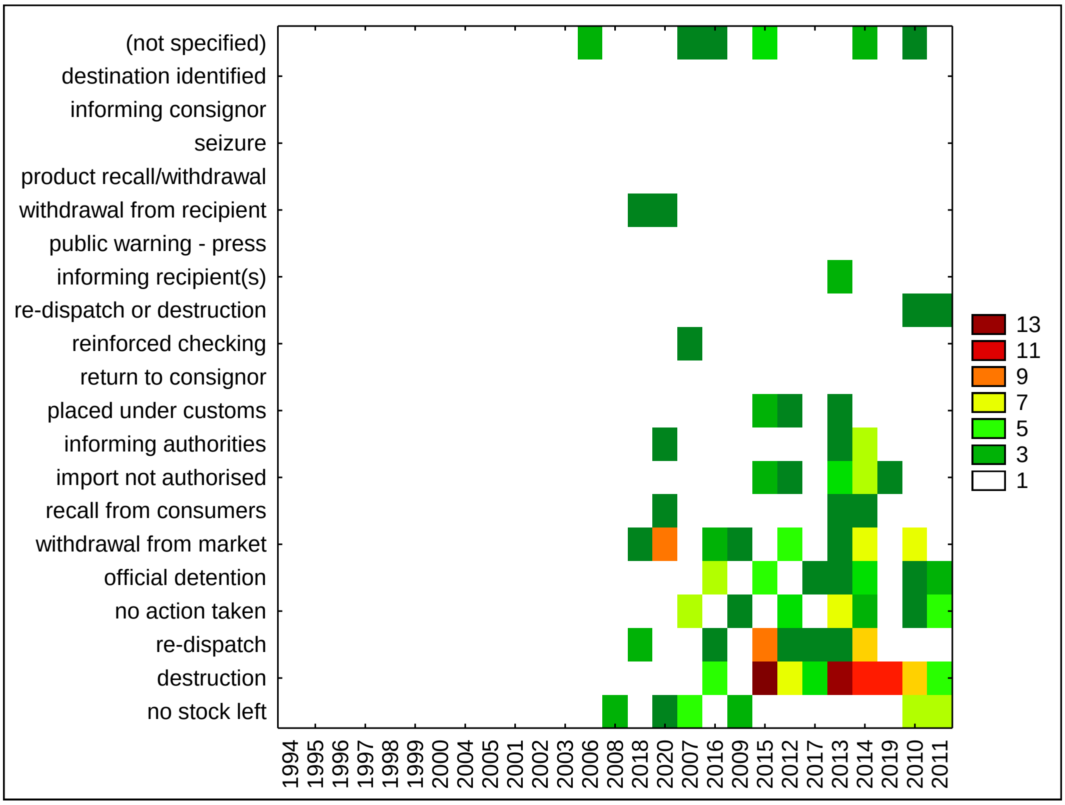

Figure S4g. Results of two-way joining cluster analysis for carbendazim (action taken)

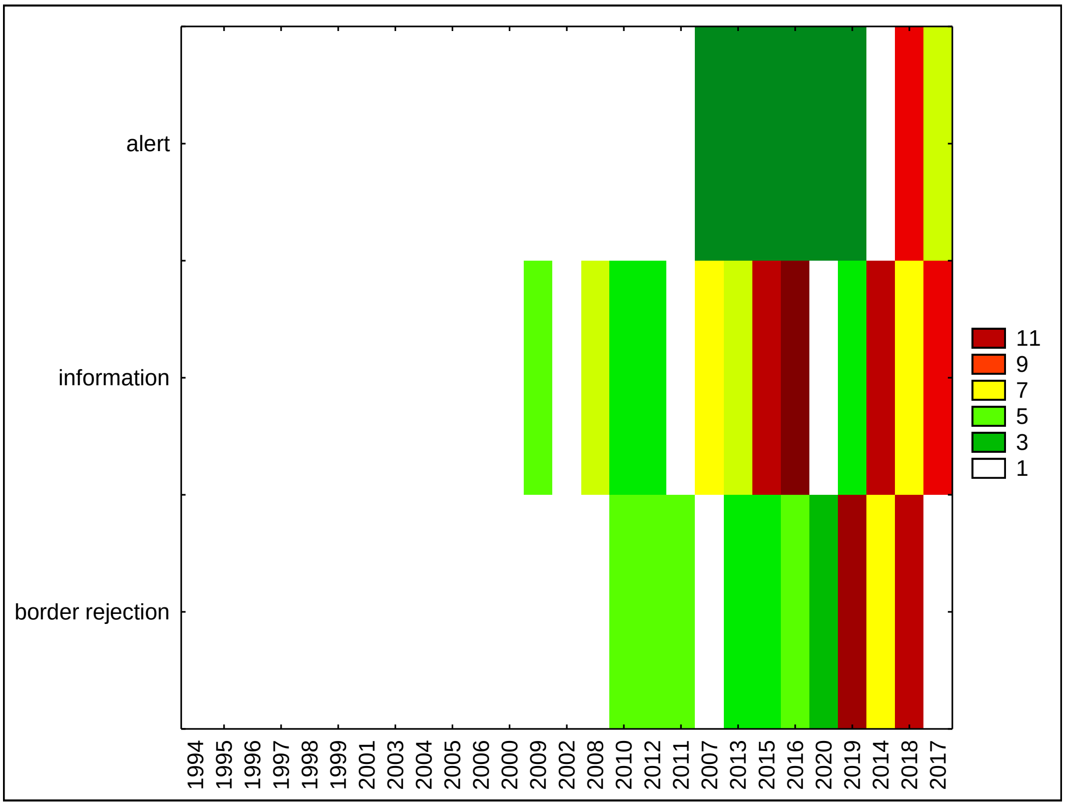

Figure S5a. Results of two-way joining cluster analysis for carbofuran (notification type)

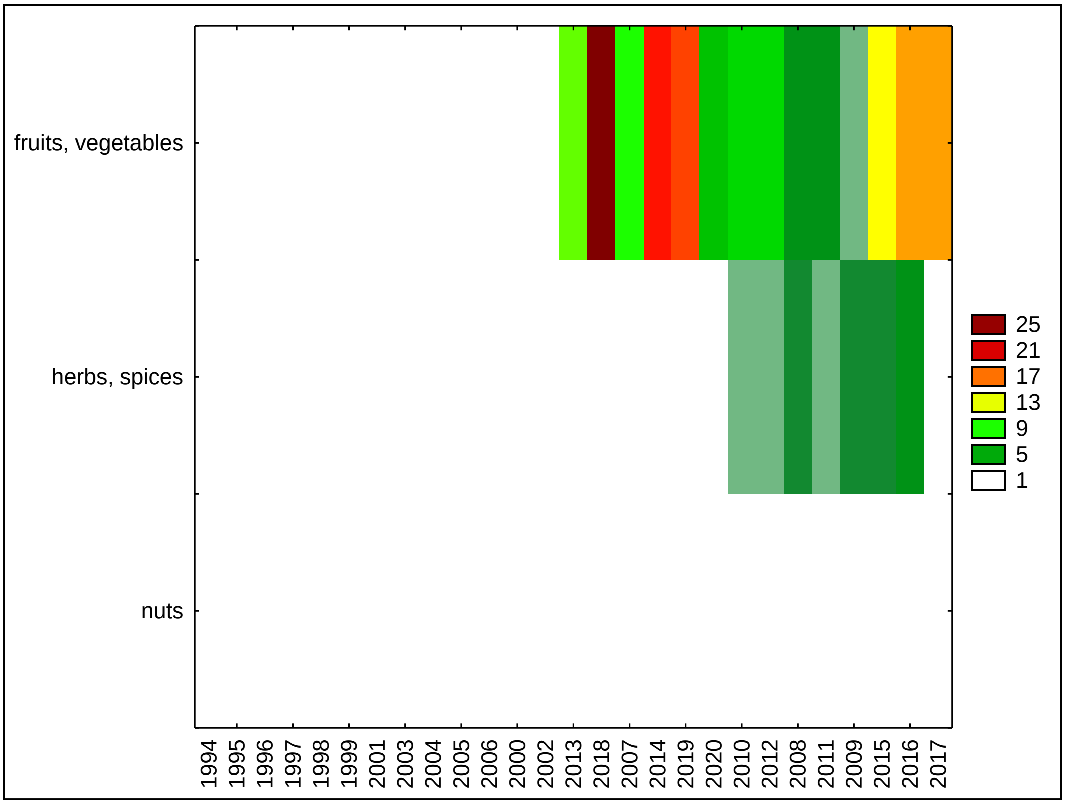

Figure S5b. Results of two-way joining cluster analysis for carbofuran (product category)

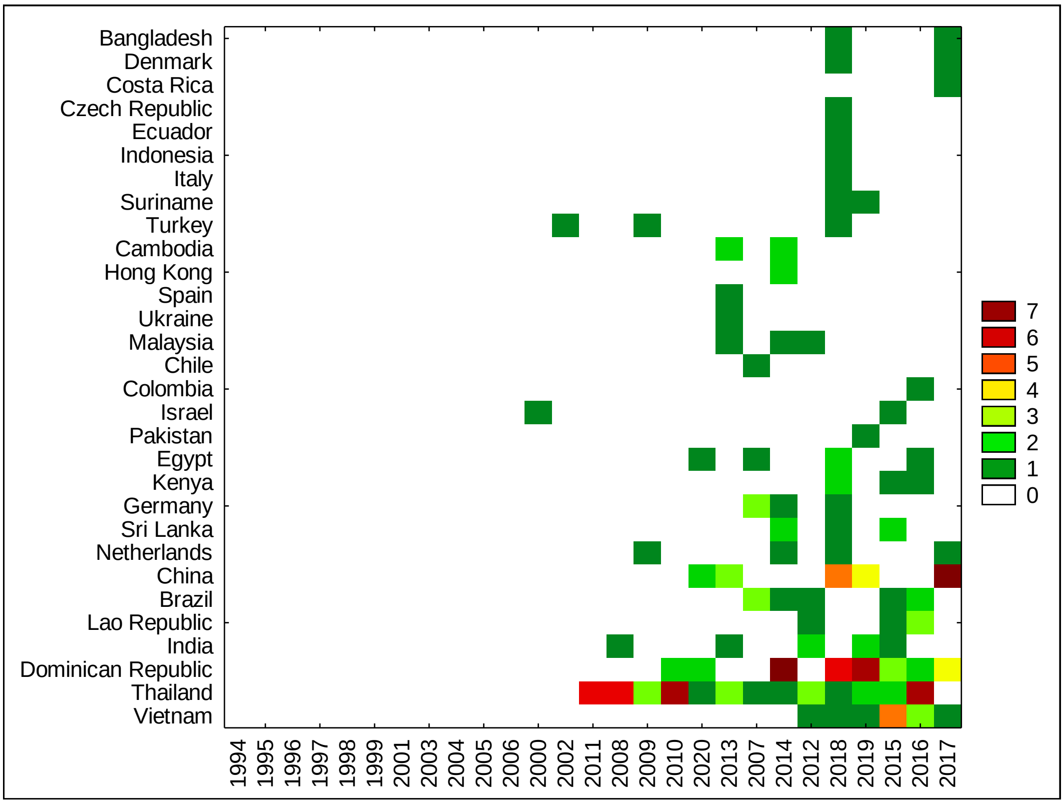

Figure S5c. Results of two-way joining cluster analysis for carbofuran (origin country)

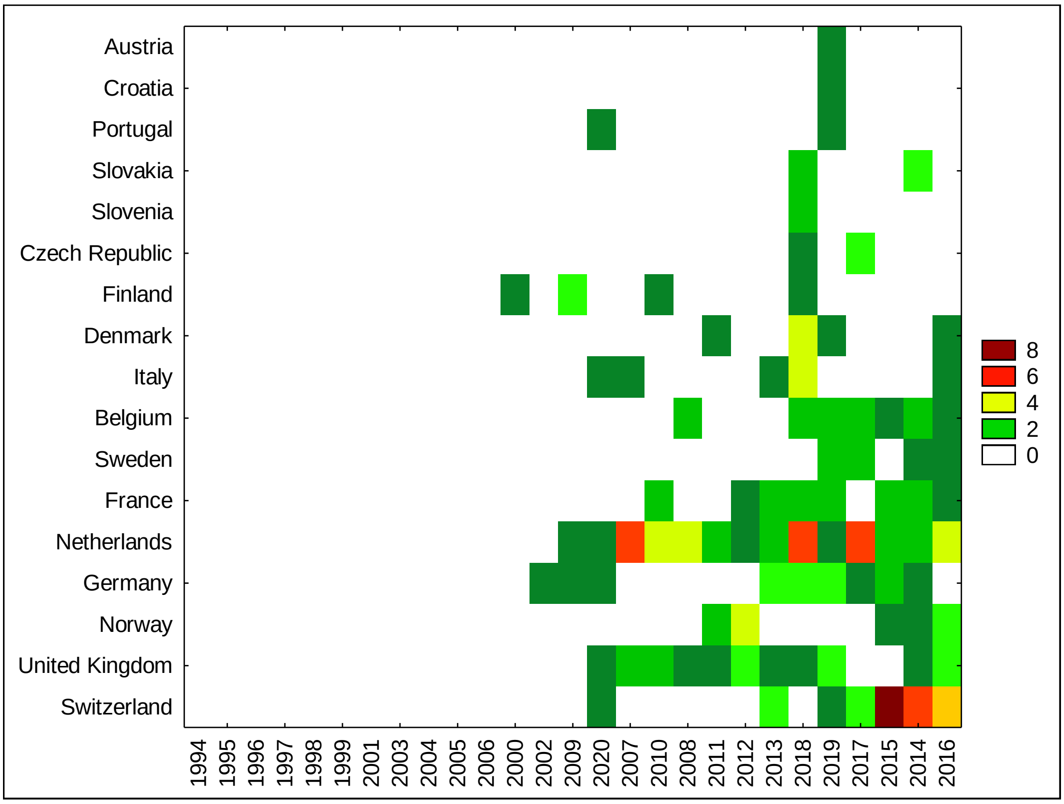

Figure S5d. Results of two-way joining cluster analysis for carbofuran (notifying country)

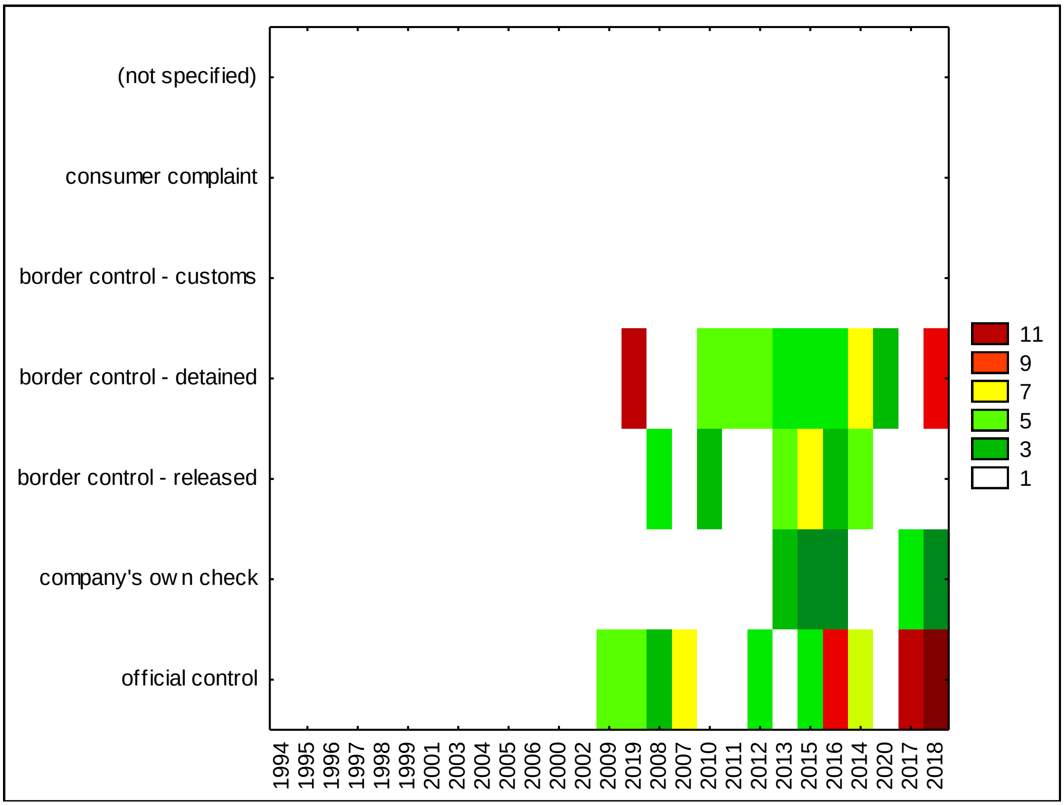

Figure S5e. Results of two-way joining cluster analysis for carbofuran (notification basis)

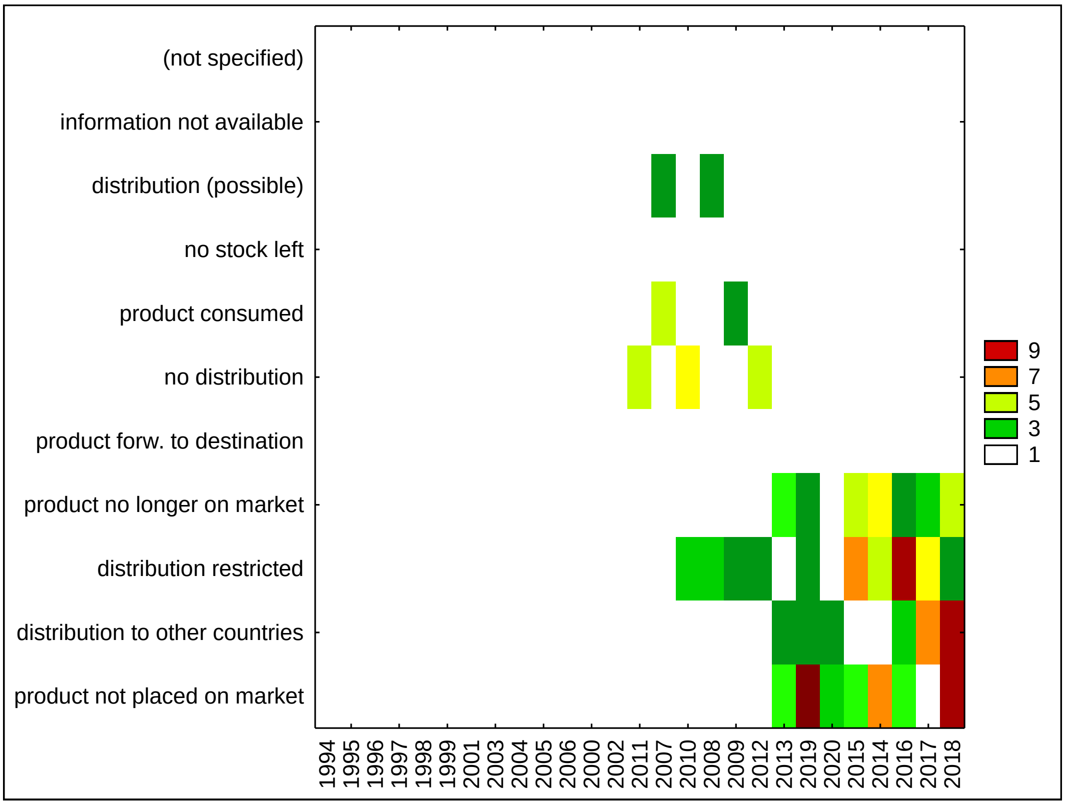

Figure S5f. Results of two-way joining cluster analysis for carbofuran (distribution status)

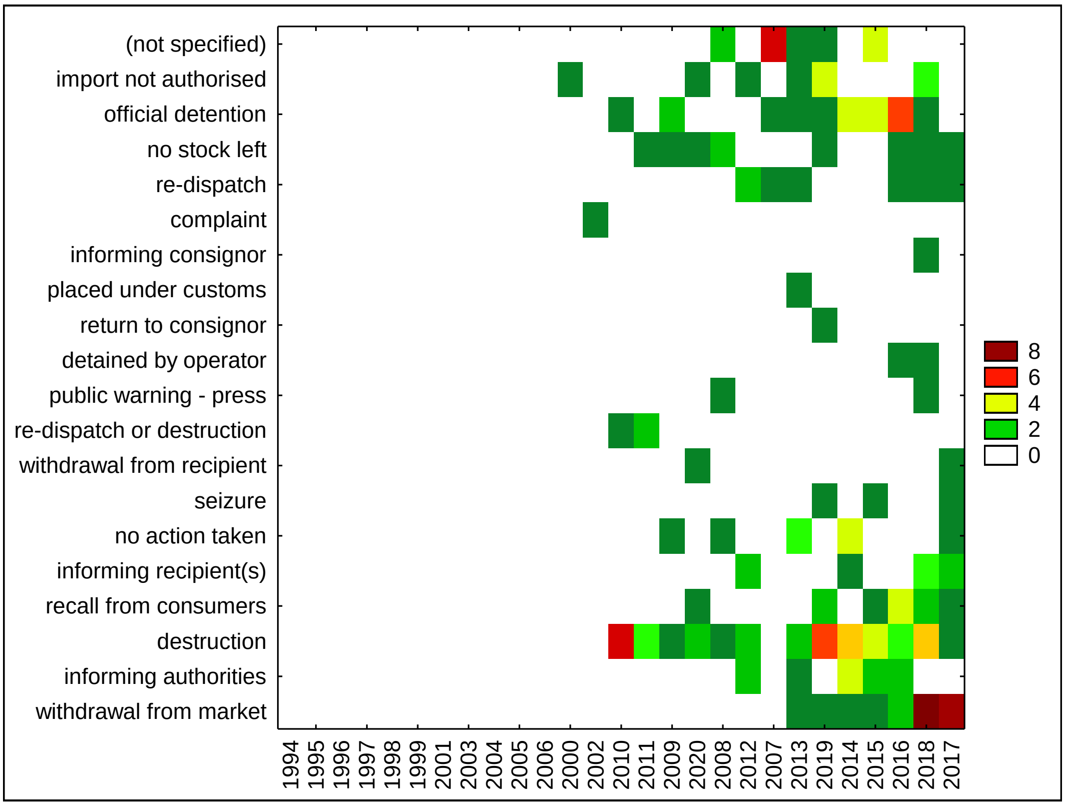

Figure S5g. Results of two-way joining cluster analysis for carbofuran (action taken)

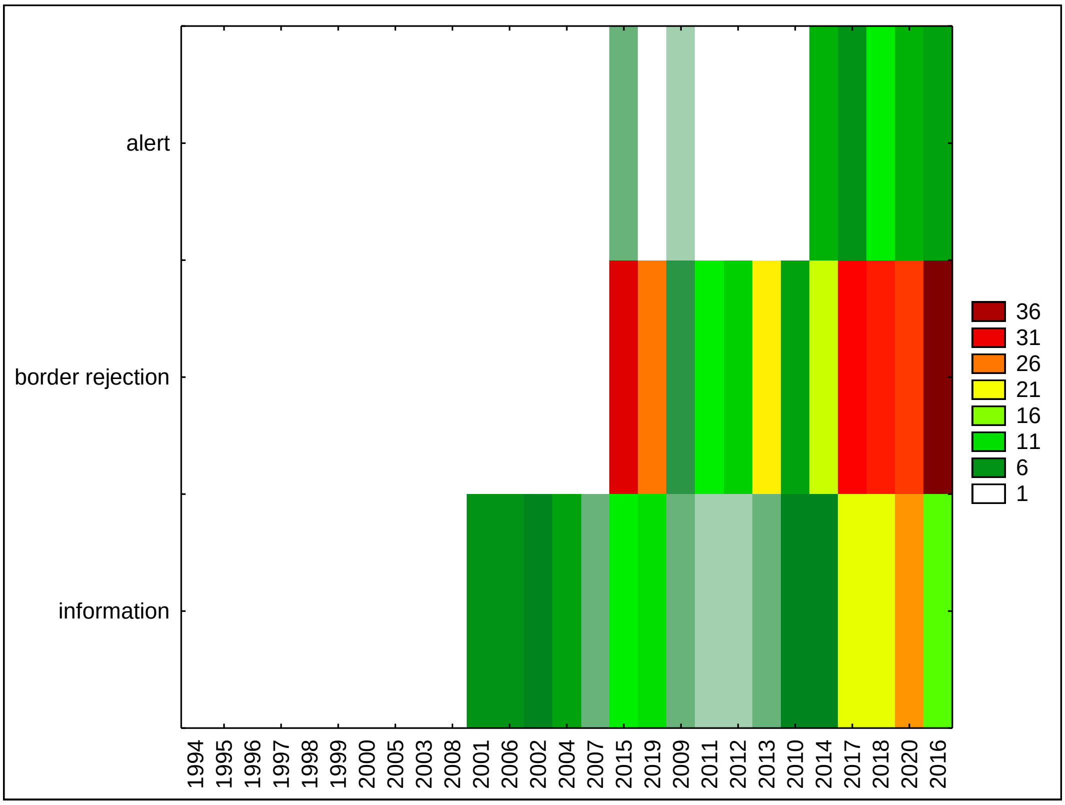

Figure S6a. Results of two-way joining cluster analysis for chlorpyrifos (notification type)

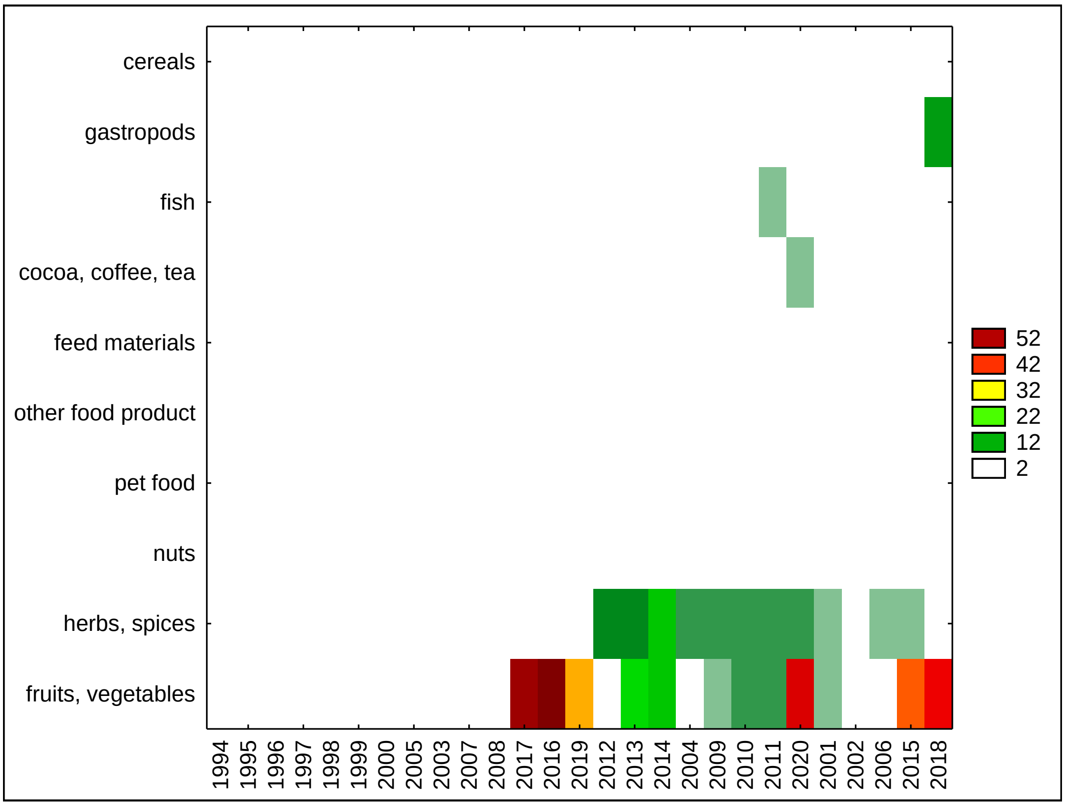

Figure S6b. Results of two-way joining cluster analysis for chlorpyrifos (product category)

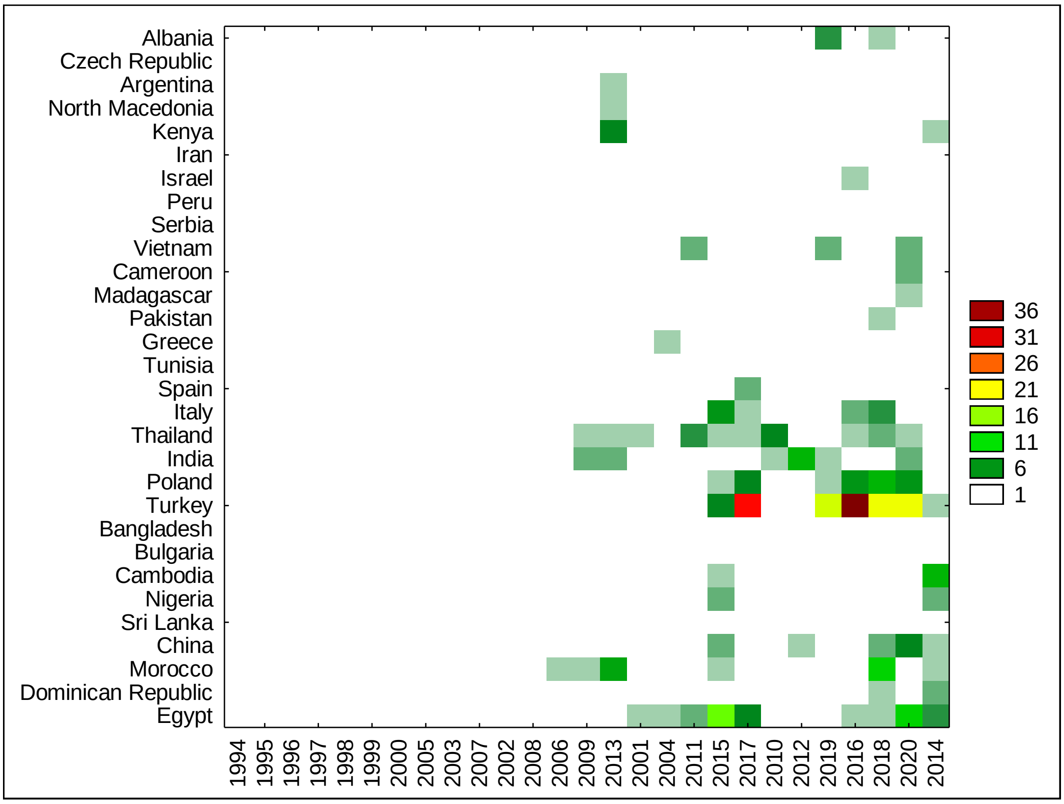

Figure S6c. Results of two-way joining cluster analysis for chlorpyrifos (origin country)

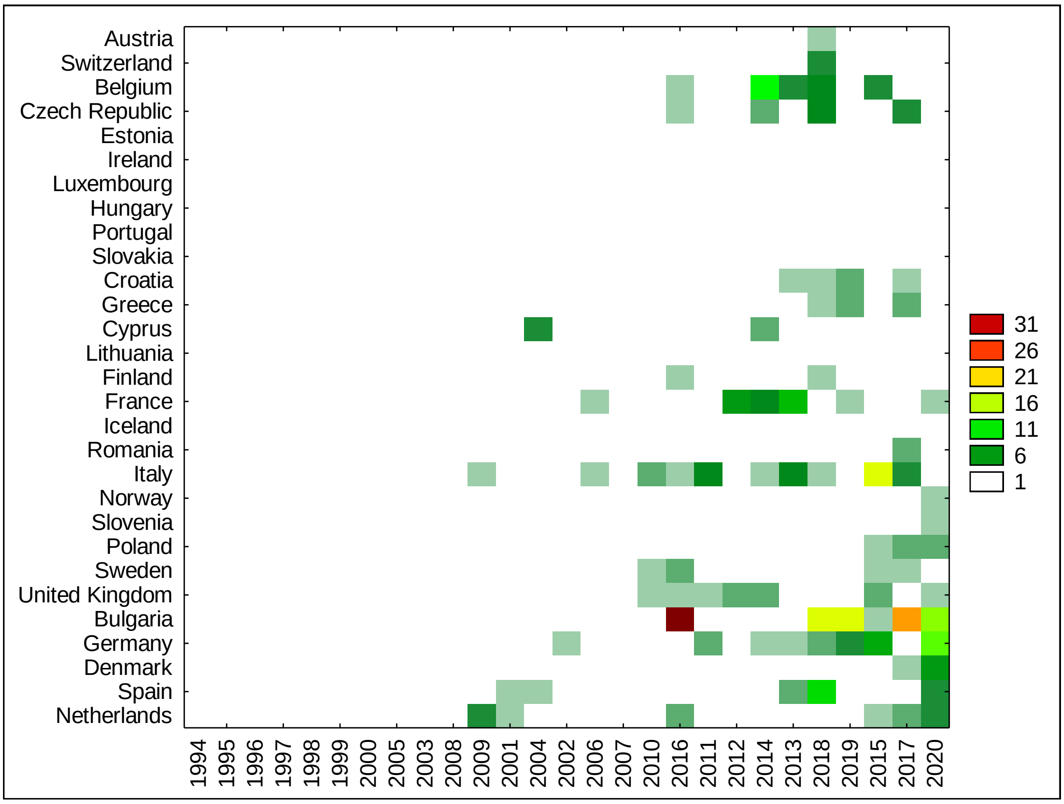

Figure S6d. Results of two-way joining cluster analysis for chlorpyrifos (notifying country)

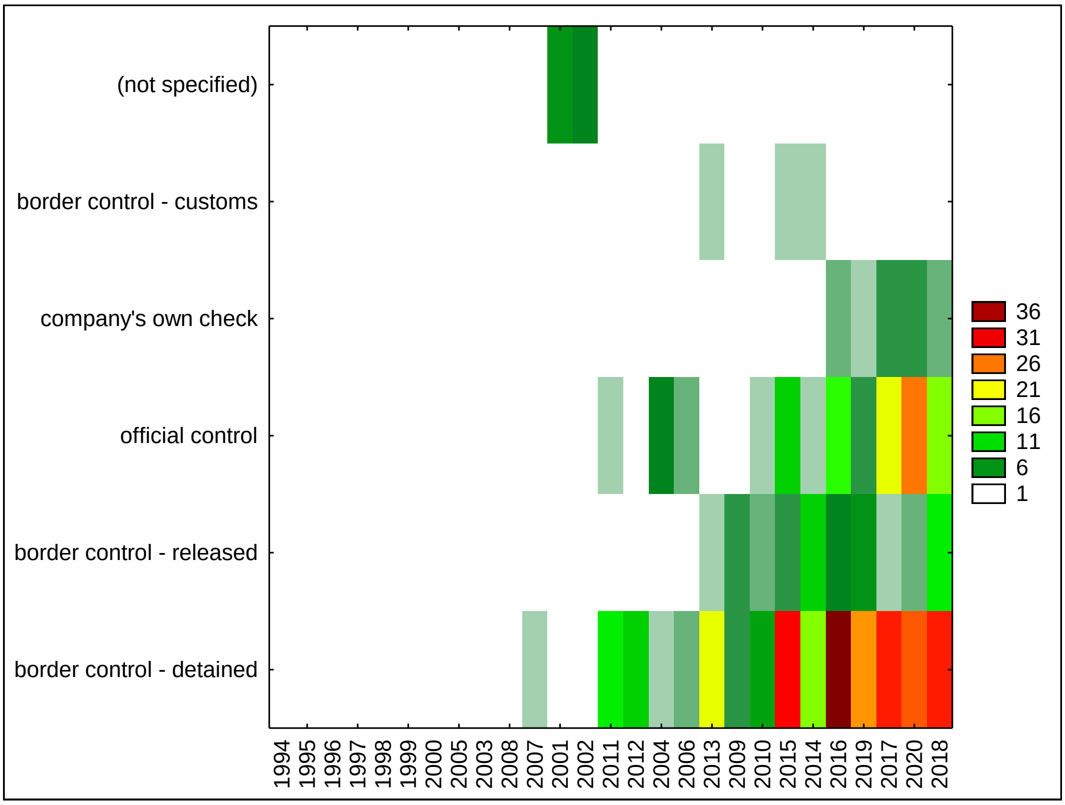

Figure S6e. Results of two-way joining cluster analysis for chlorpyrifos (notification basis)

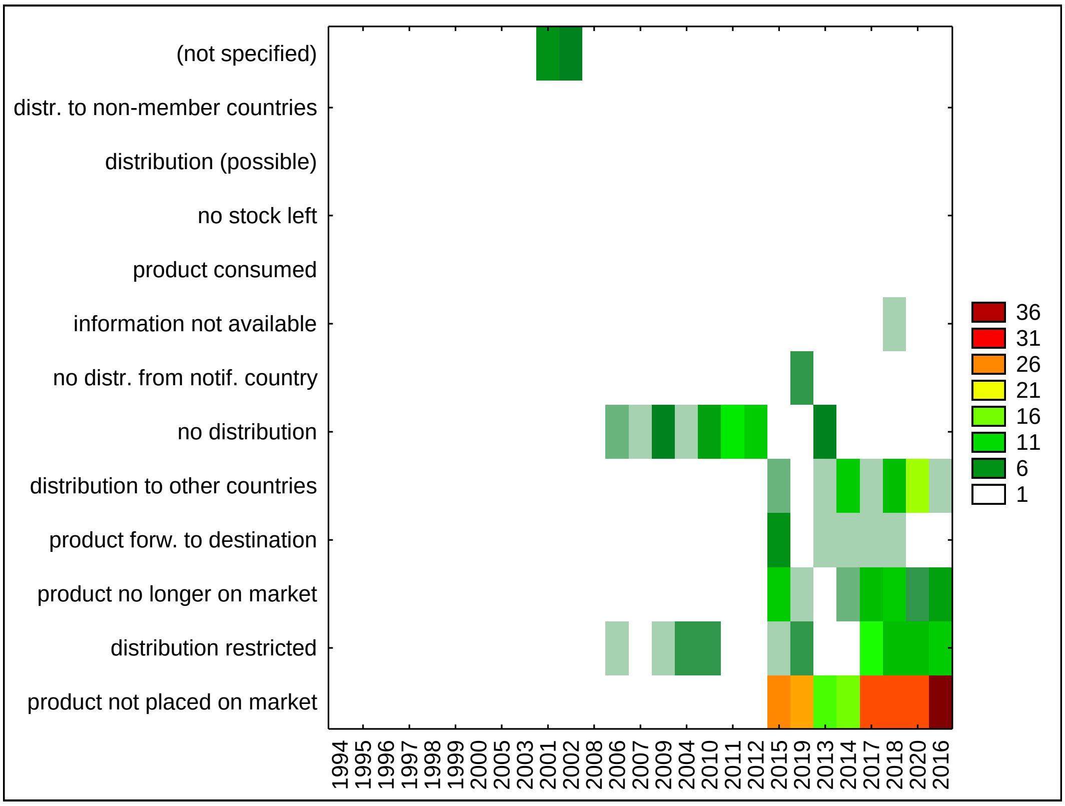

Figure S6f. Results of two-way joining cluster analysis for chlorpyrifos (distribution status)

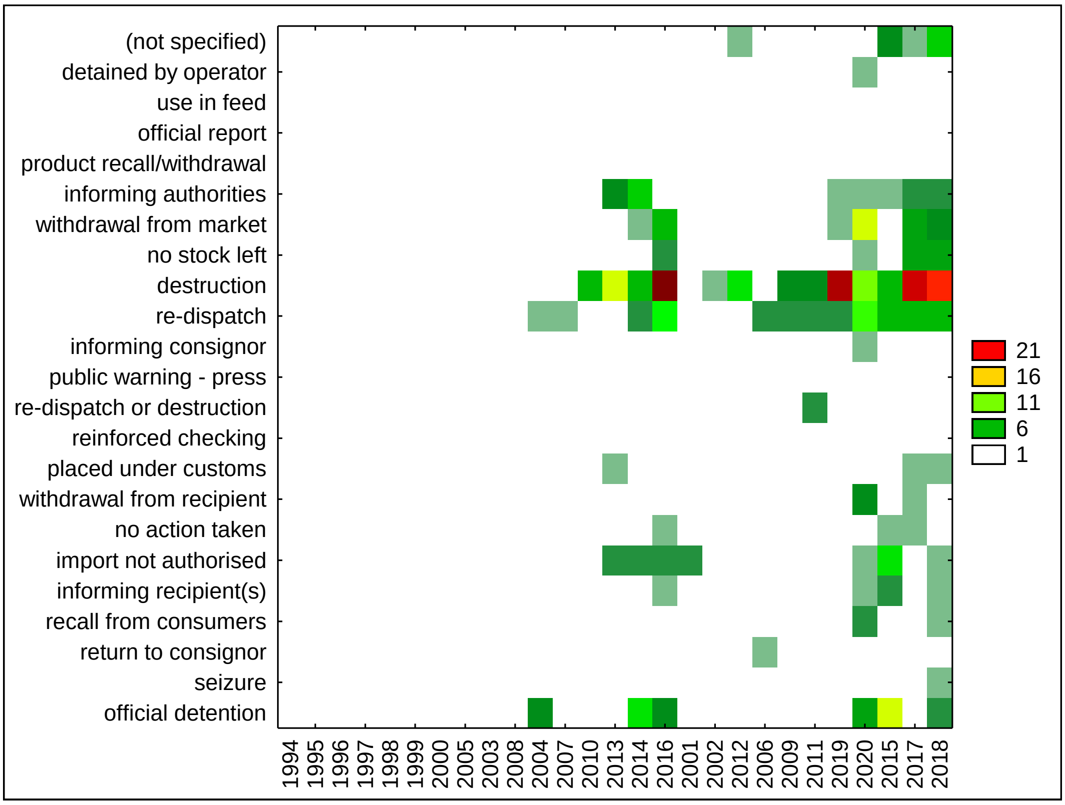

Figure S6g. Results of two-way joining cluster analysis for chlorpyrifos (action taken)

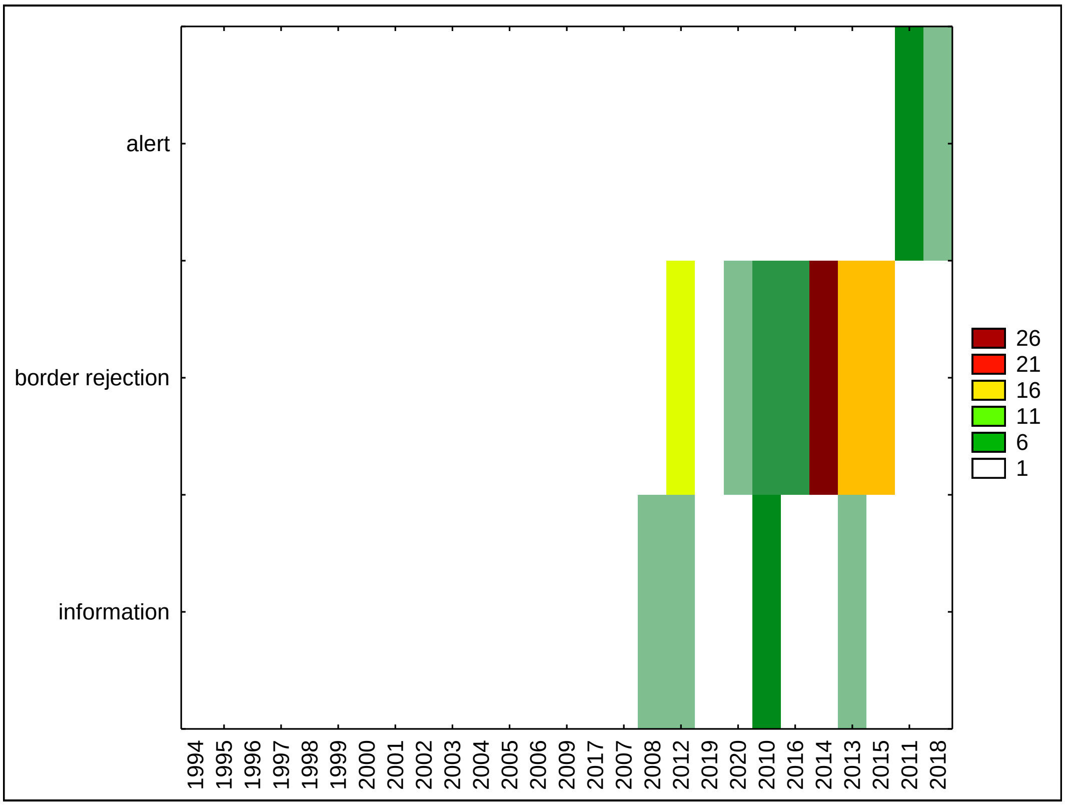

Figure S7a. Results of two-way joining cluster analysis for dichlorvos (notification type)

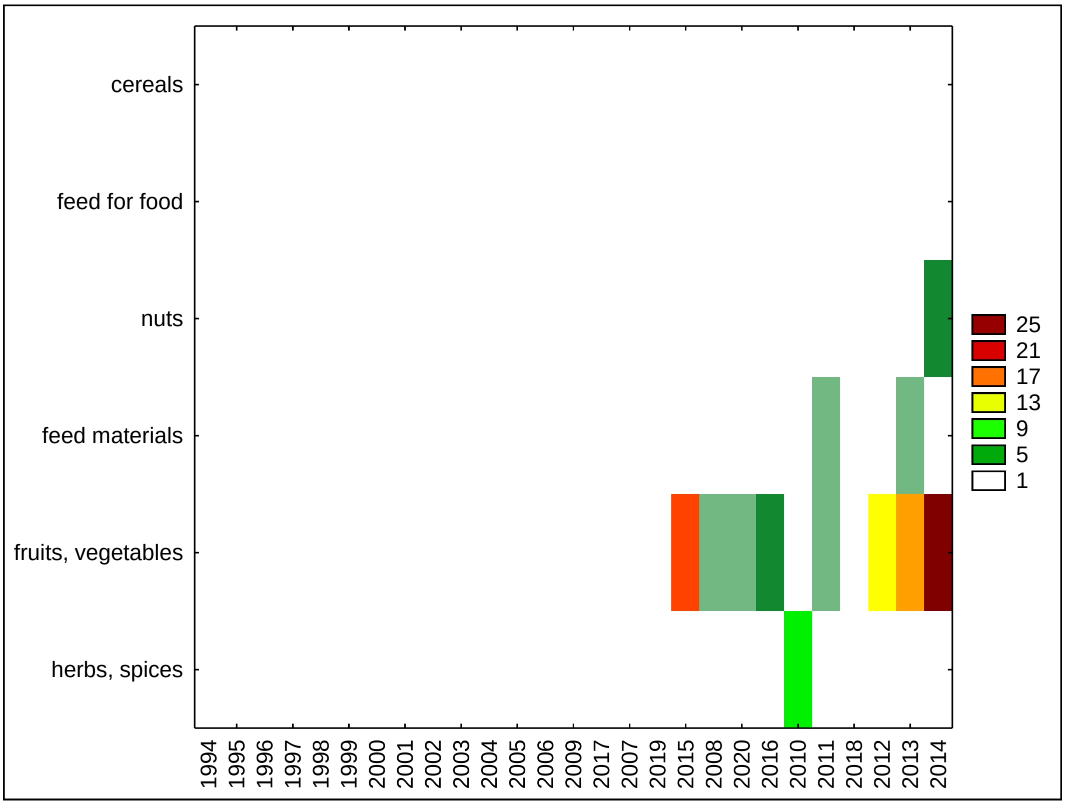

Figure S7b. Results of two-way joining cluster analysis for dichlorvos (product category)

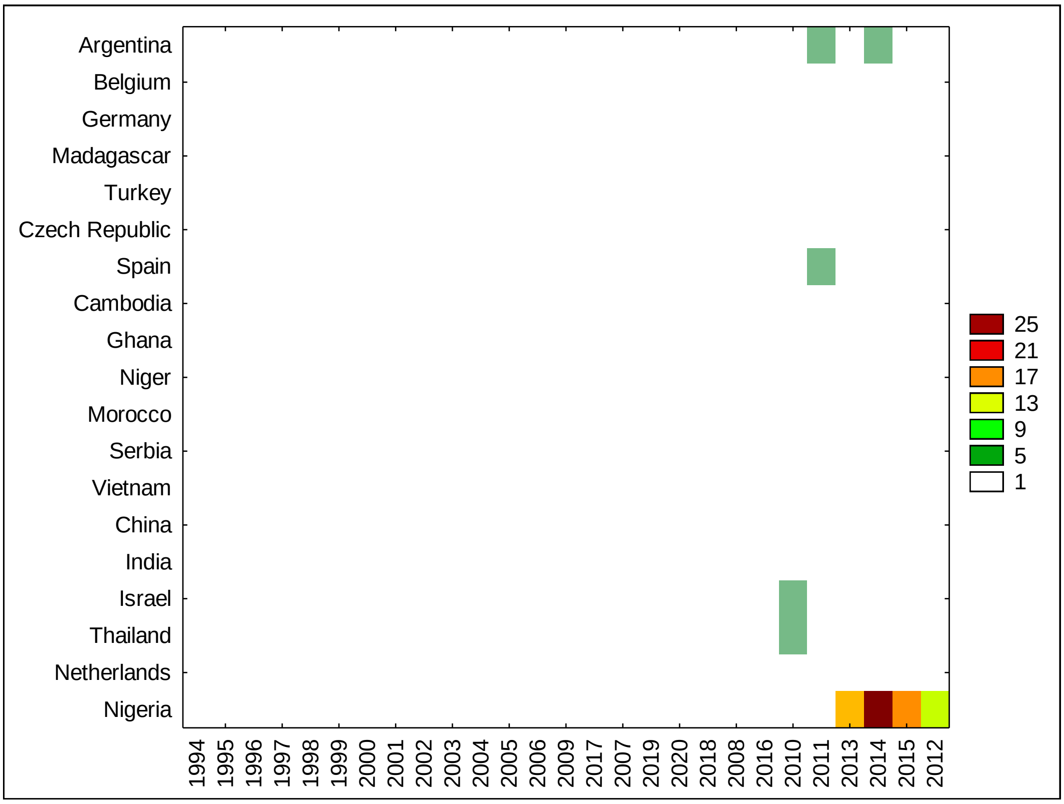

Figure S7c. Results of two-way joining cluster analysis for dichlorvos (origin country)

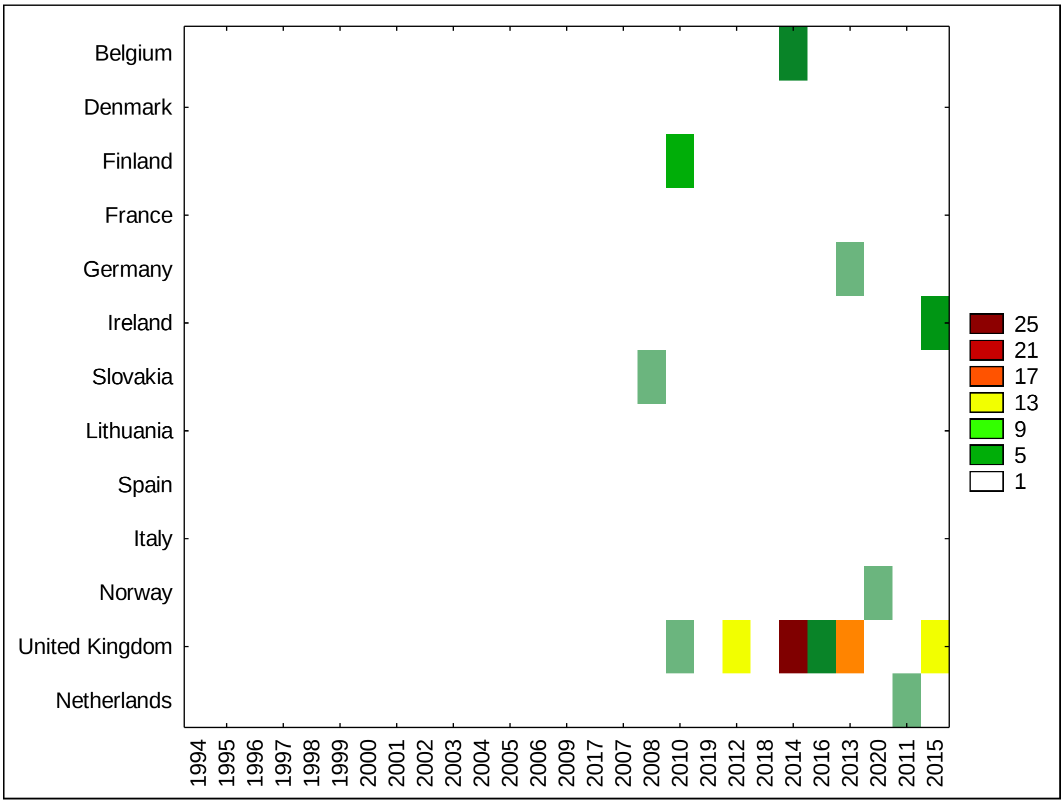

Figure S7d. Results of two-way joining cluster analysis for dichlorvos (notifying country)

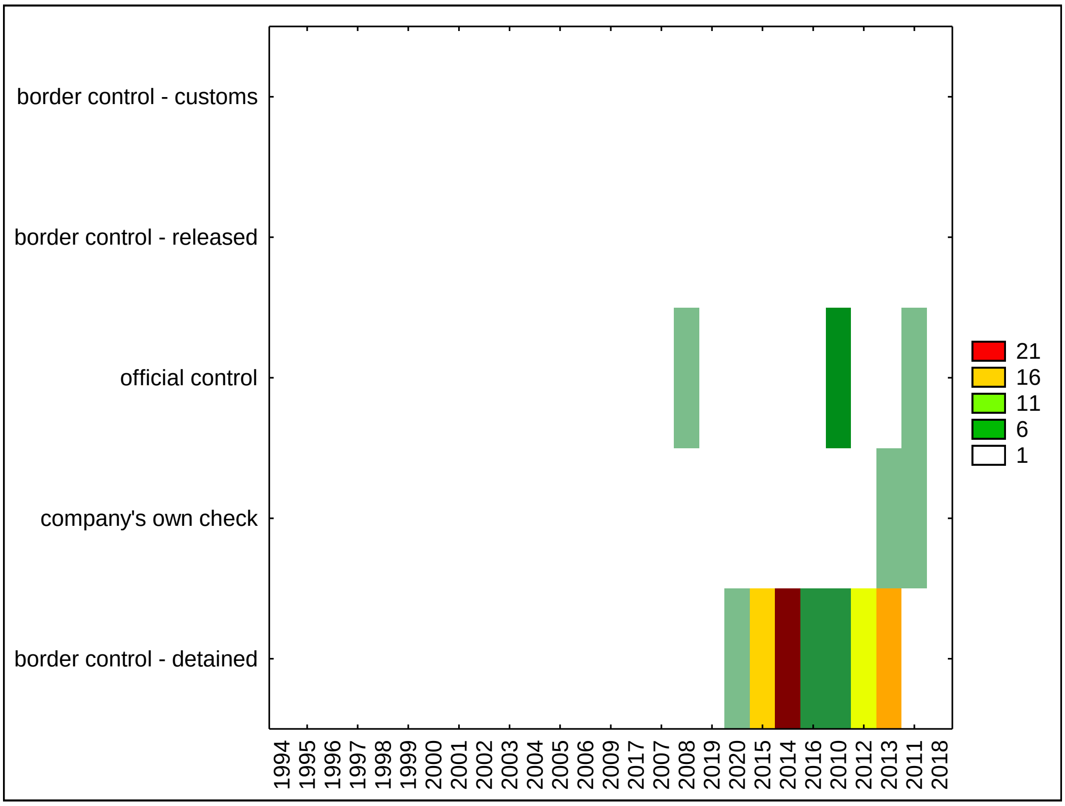

Figure S7e. Results of two-way joining cluster analysis for dichlorvos (notification basis)

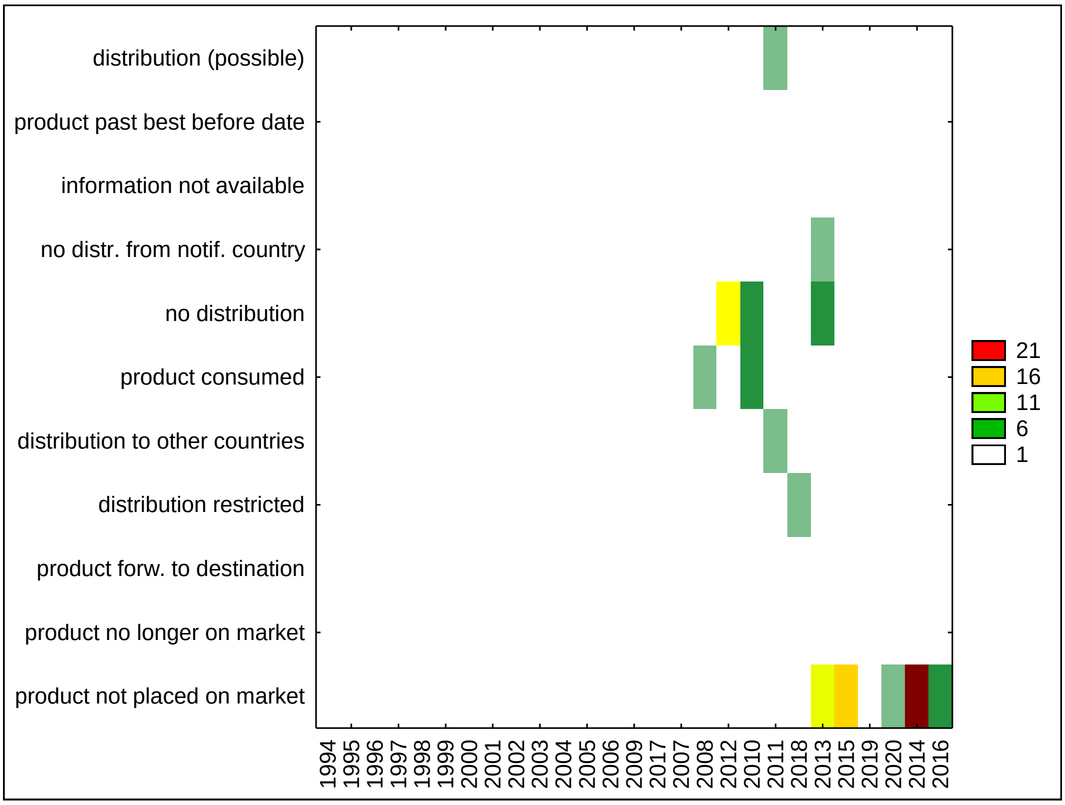

Figure S7f. Results of two-way joining cluster analysis for dichlorvos (distribution status)

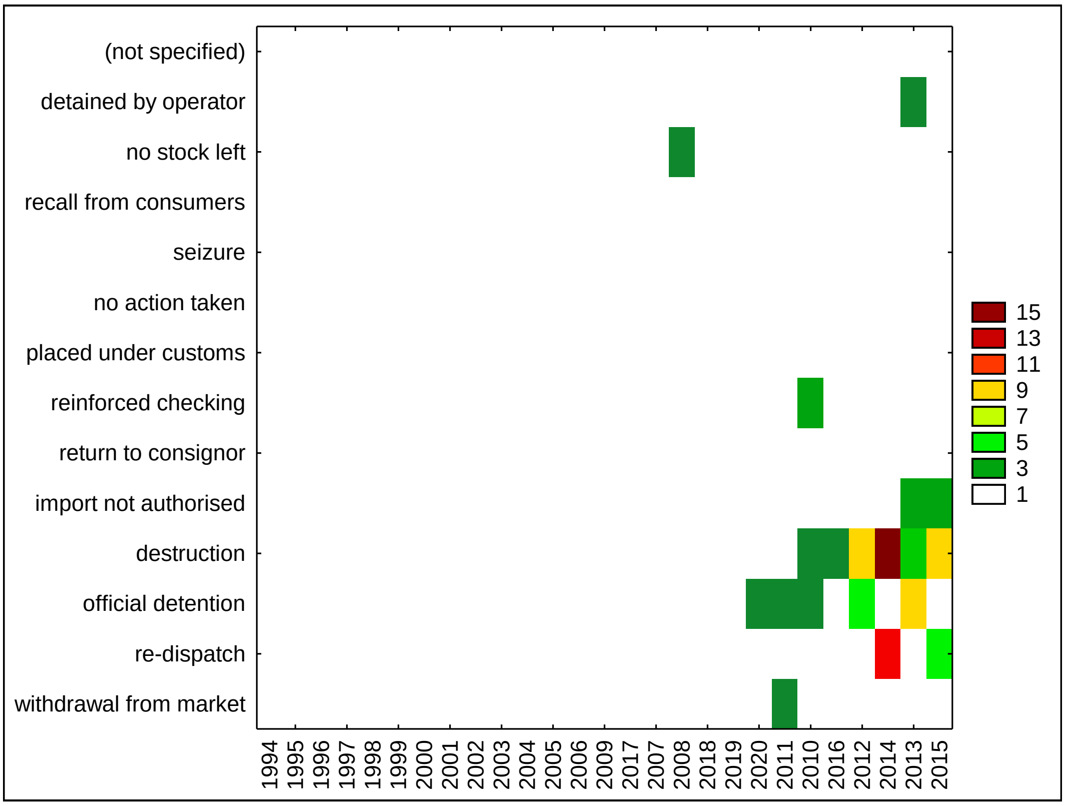

Figure S7g. Results of two-way joining cluster analysis for dichlorvos (action taken)

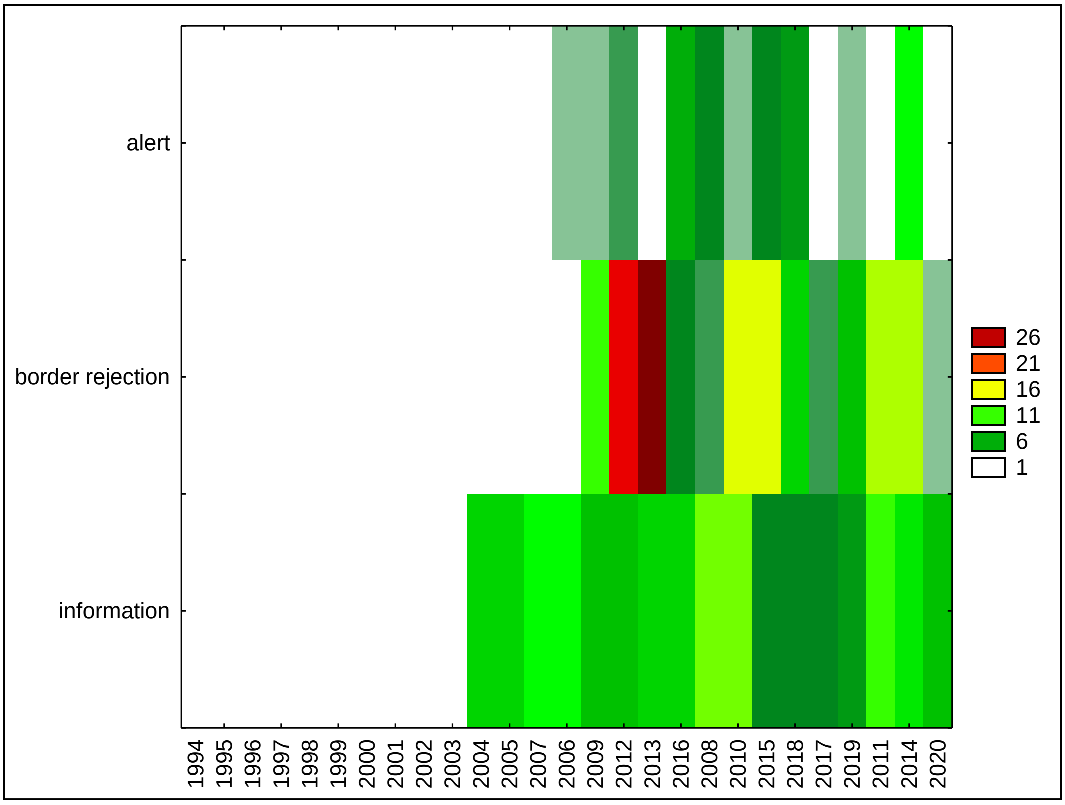

Figure S8a. Results of two-way joining cluster analysis for dimethoate (notification type)

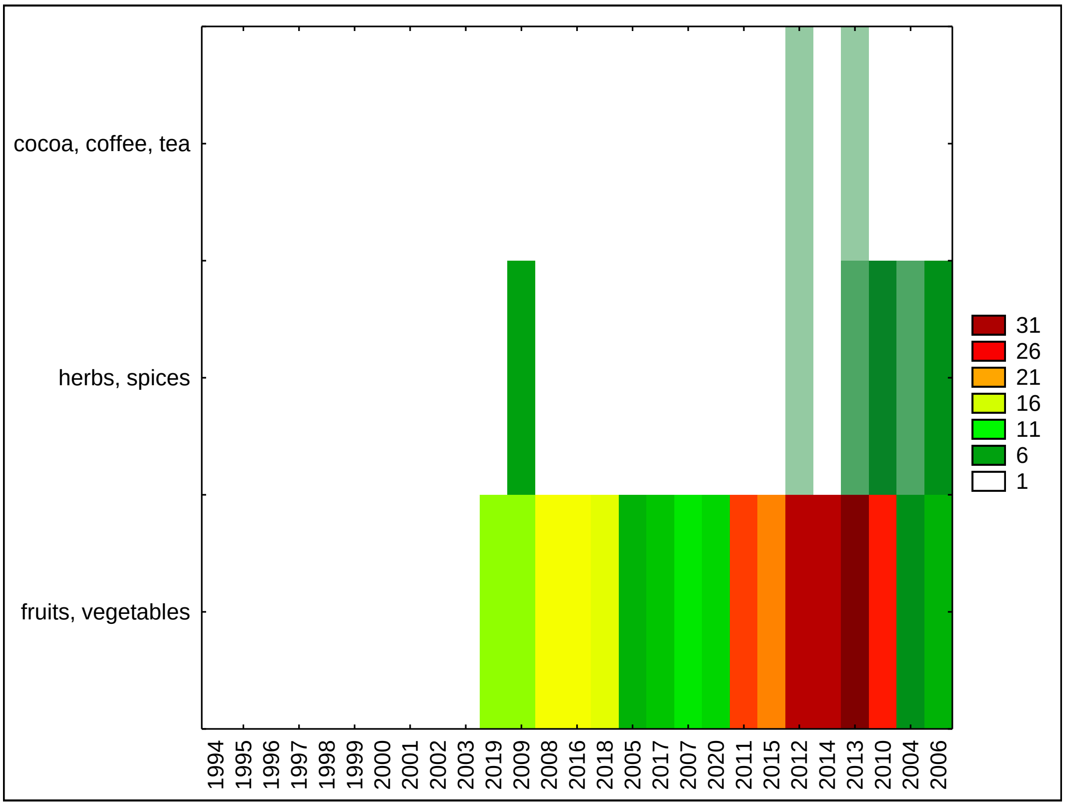

Figure S8b. Results of two-way joining cluster analysis for dimethoate (product category)

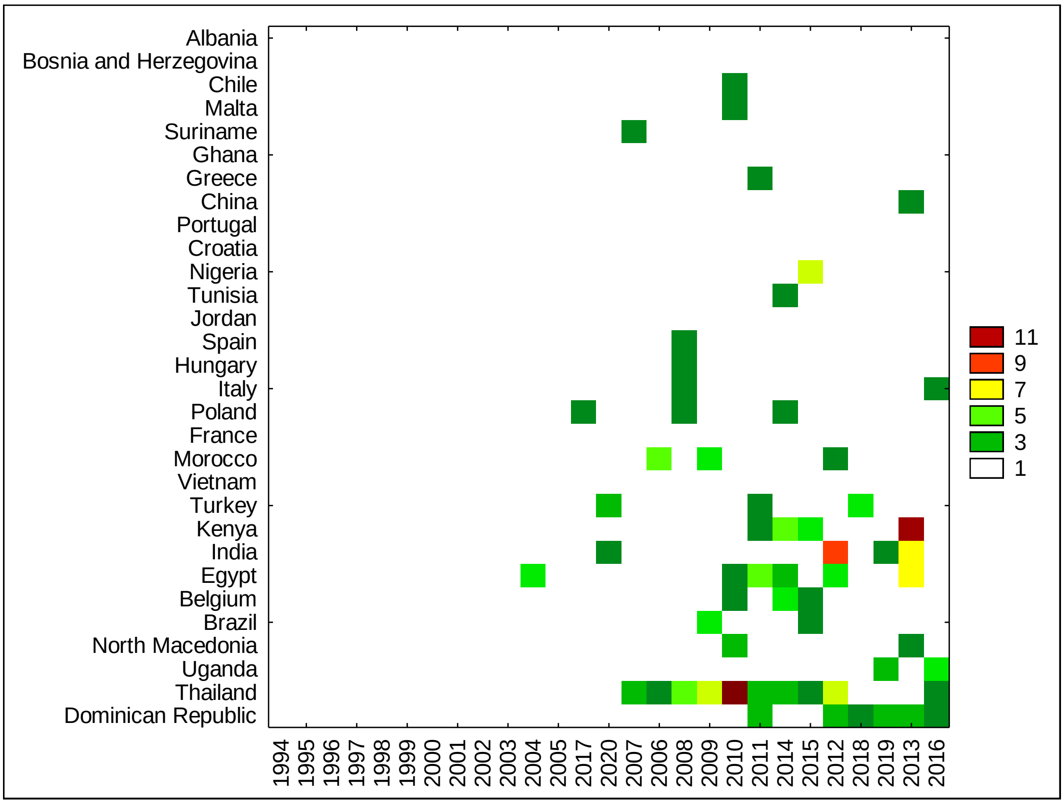

Figure S8c. Results of two-way joining cluster analysis for dimethoate (origin country)

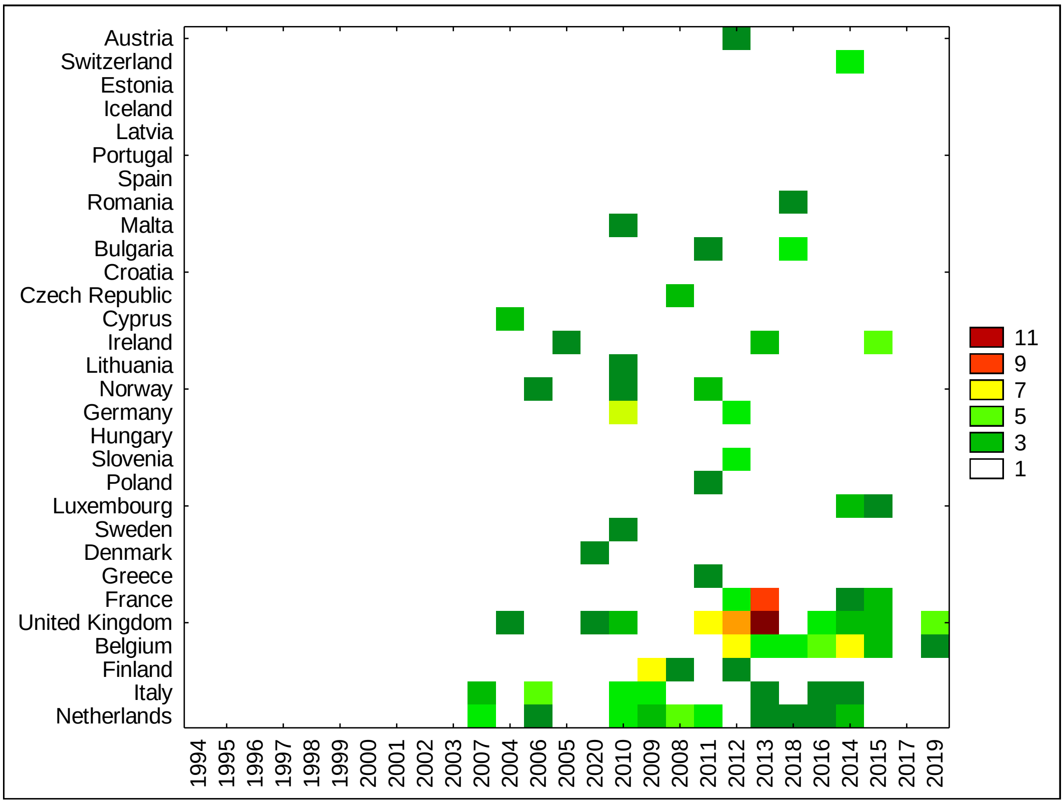

Figure S8d. Results of two-way joining cluster analysis for dimethoate (notifying country)

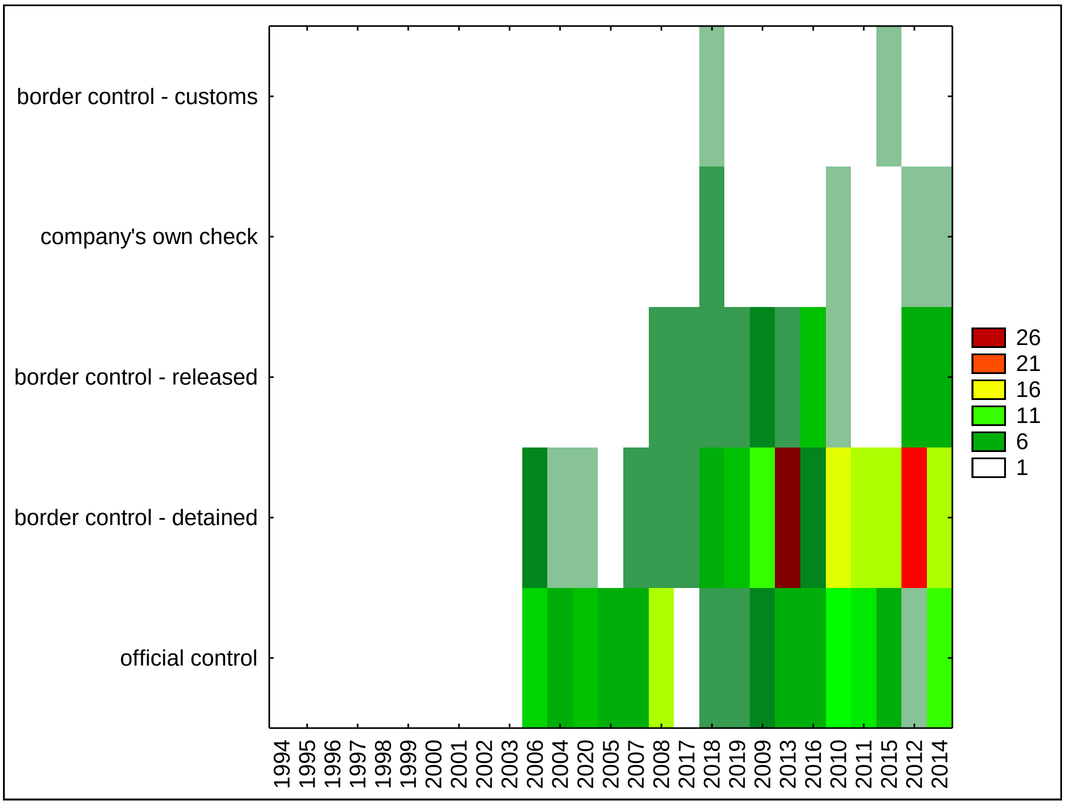

Figure S8e. Results of two-way joining cluster analysis for dimethoate (notification basis)

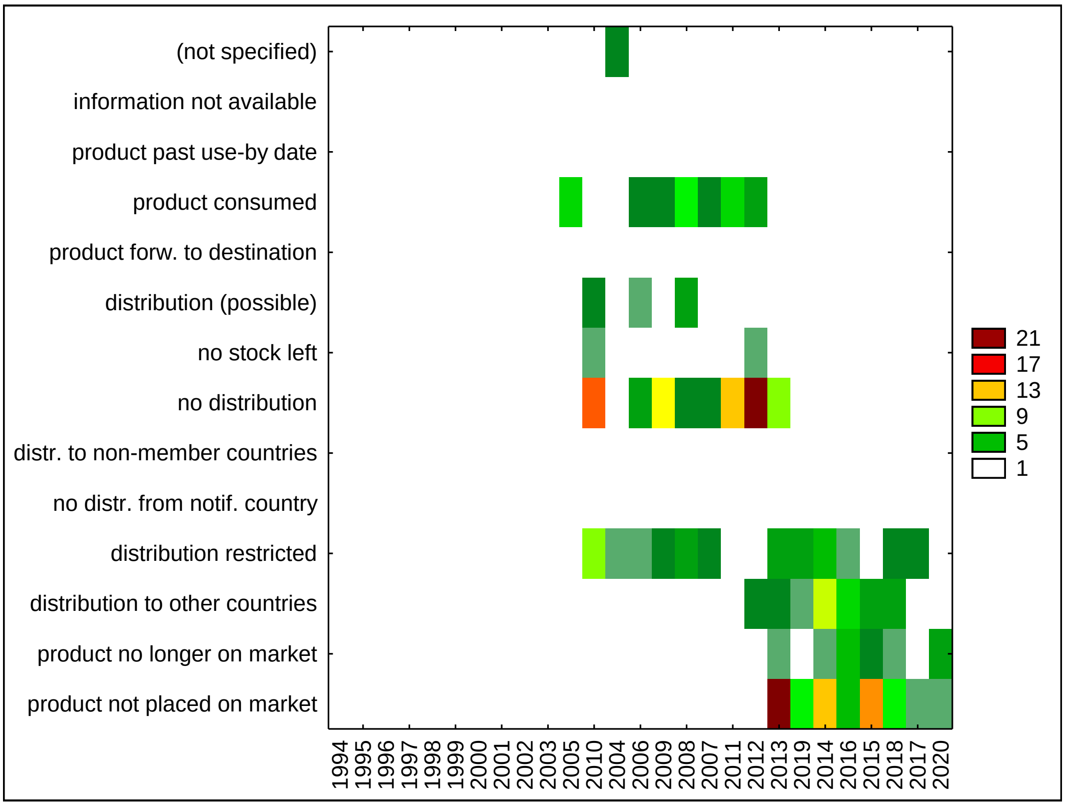

Figure S8f. Results of two-way joining cluster analysis for dimethoate (distribution status)

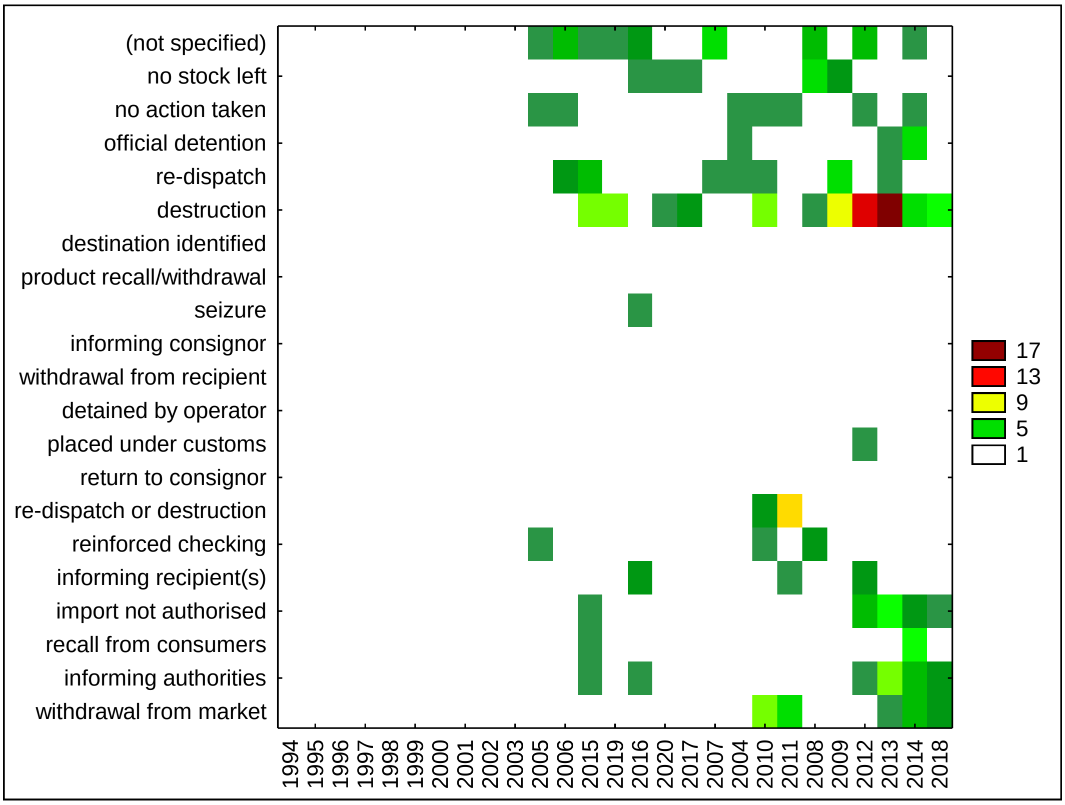

Figure S8g. Results of two-way joining cluster analysis for dimethoate (action taken)

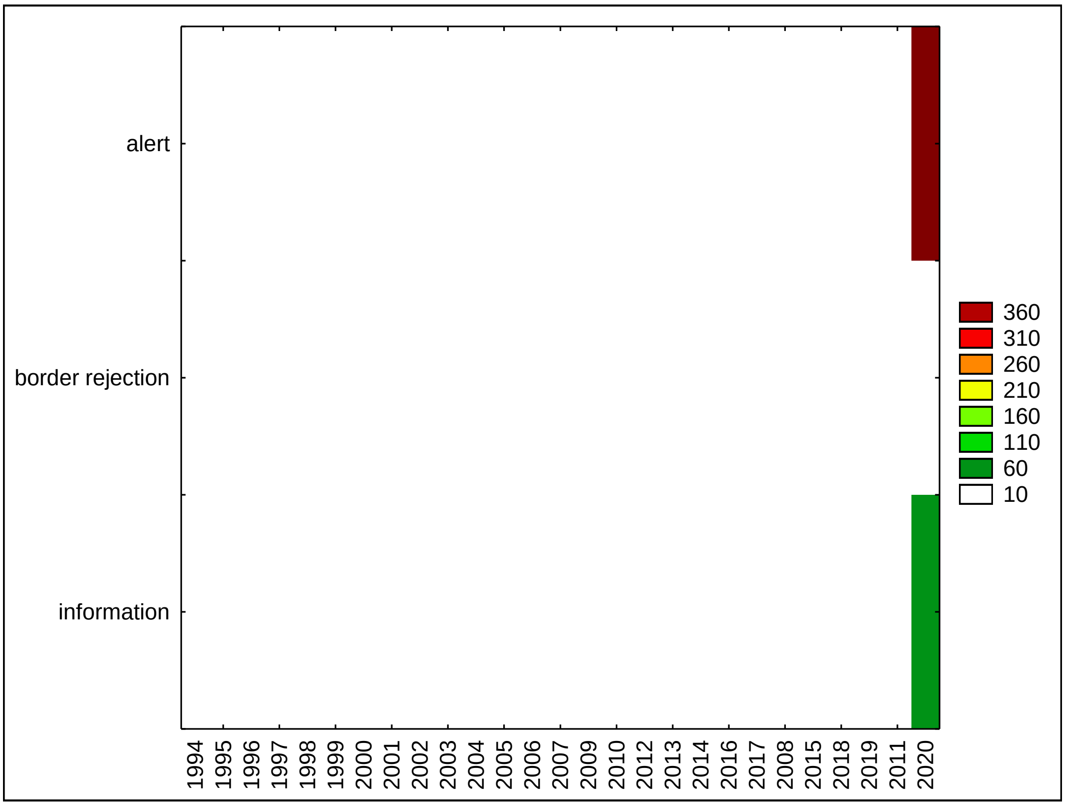

Figure S9a. Results of two-way joining cluster analysis for ethylene oxide (notification type)

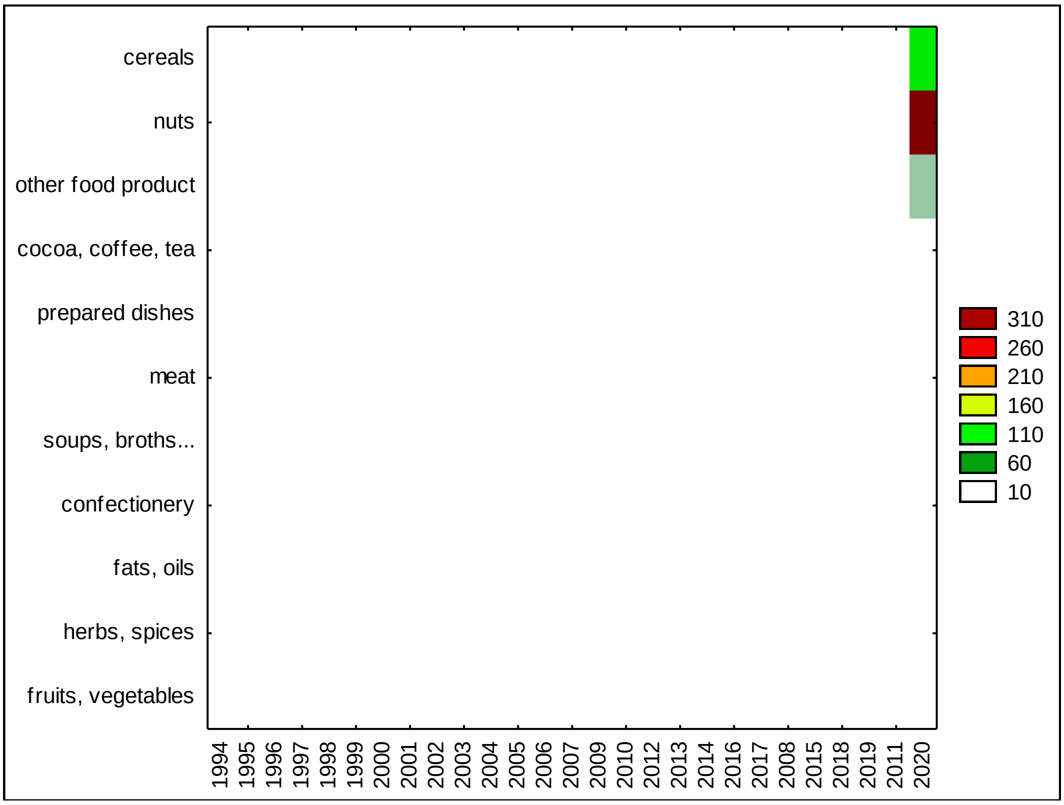

Figure S9b. Results of two-way joining cluster analysis for ethylene oxide (product category)

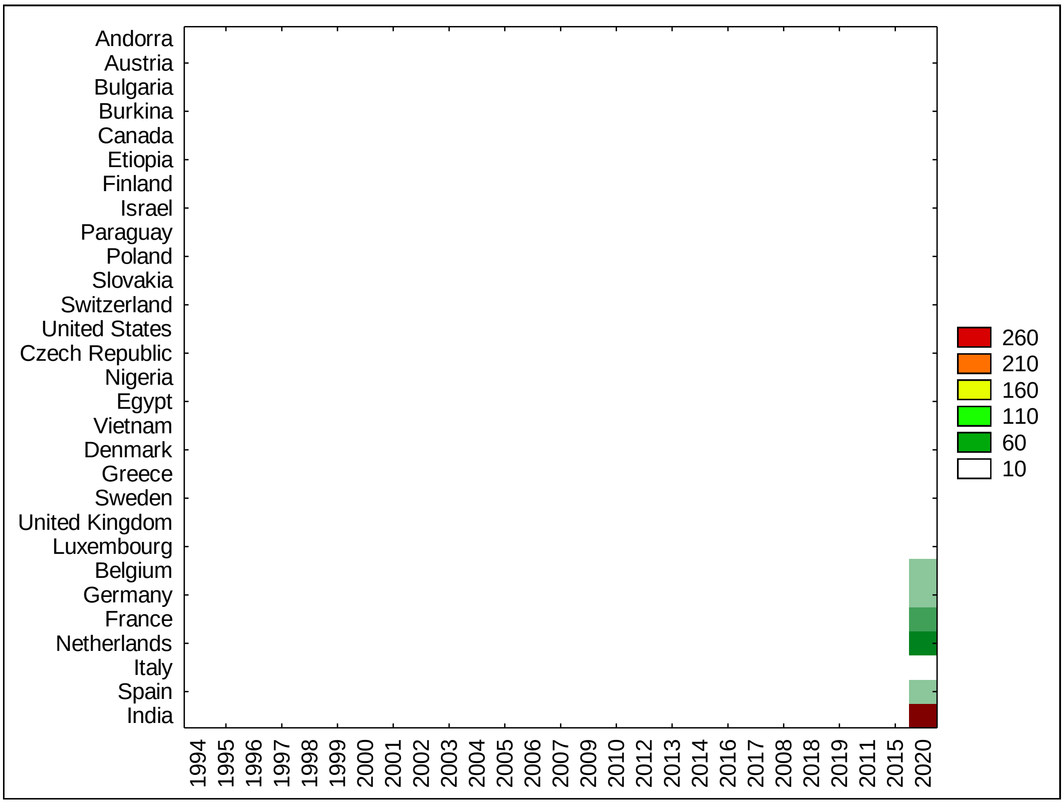

Figure S9c. Results of two-way joining cluster analysis for ethylene oxide (origin country)

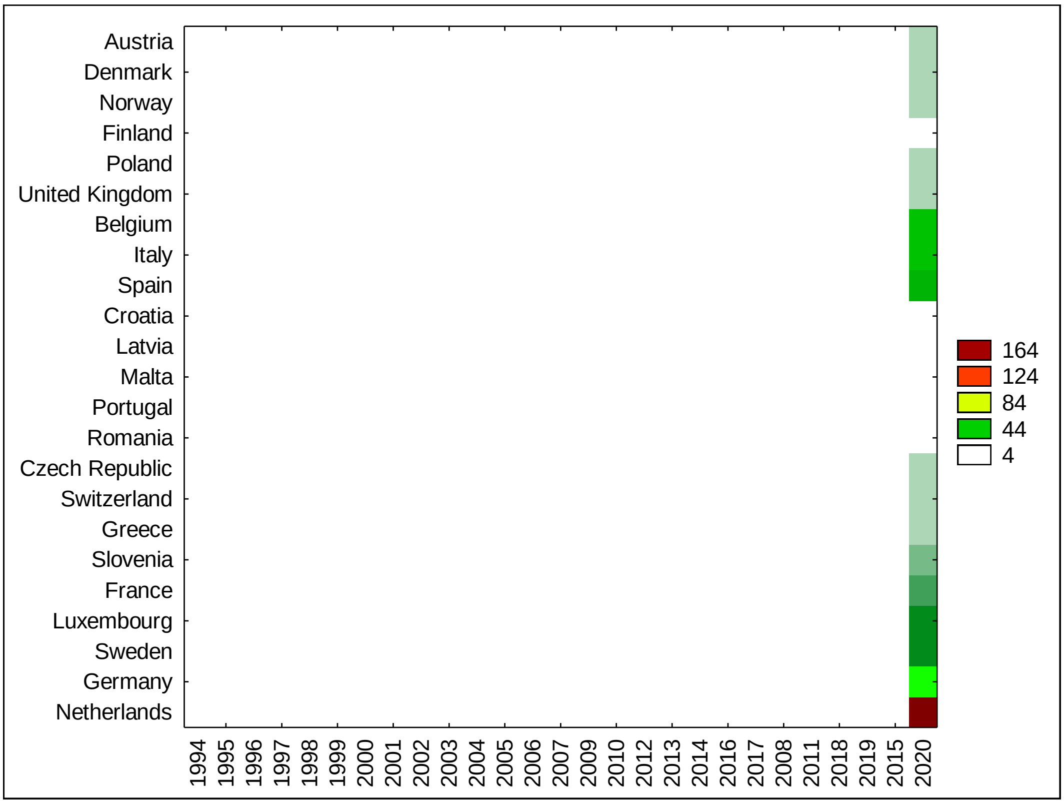

Figure S9d. Results of two-way joining cluster analysis for ethylene oxide (notifying country)

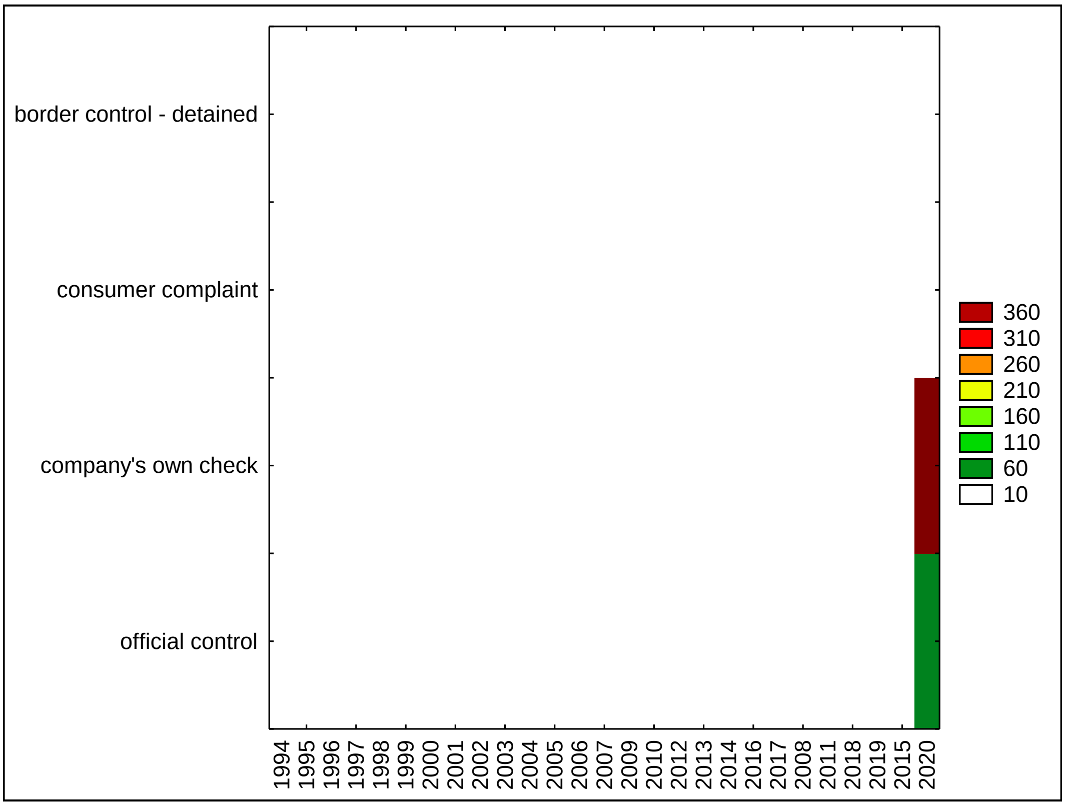

Figure S9e. Results of two-way joining cluster analysis for ethylene oxide (notification basis)

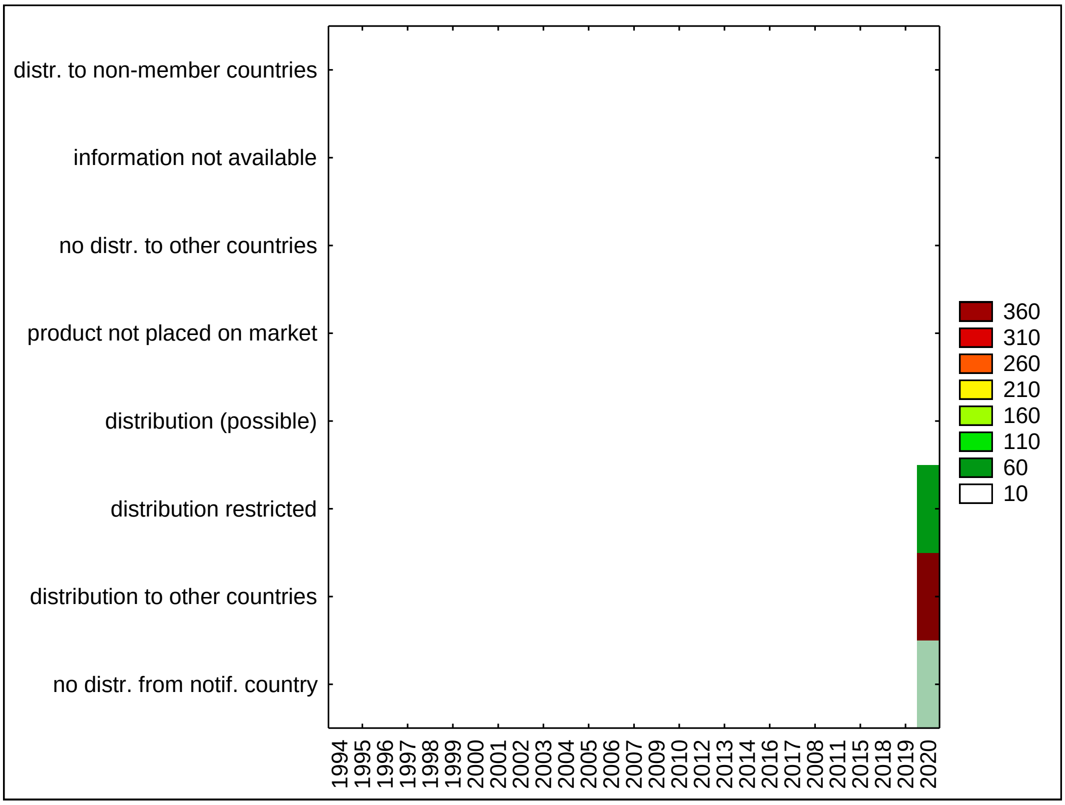

Figure S9f. Results of two-way joining cluster analysis for ethylene oxide (distribution status)

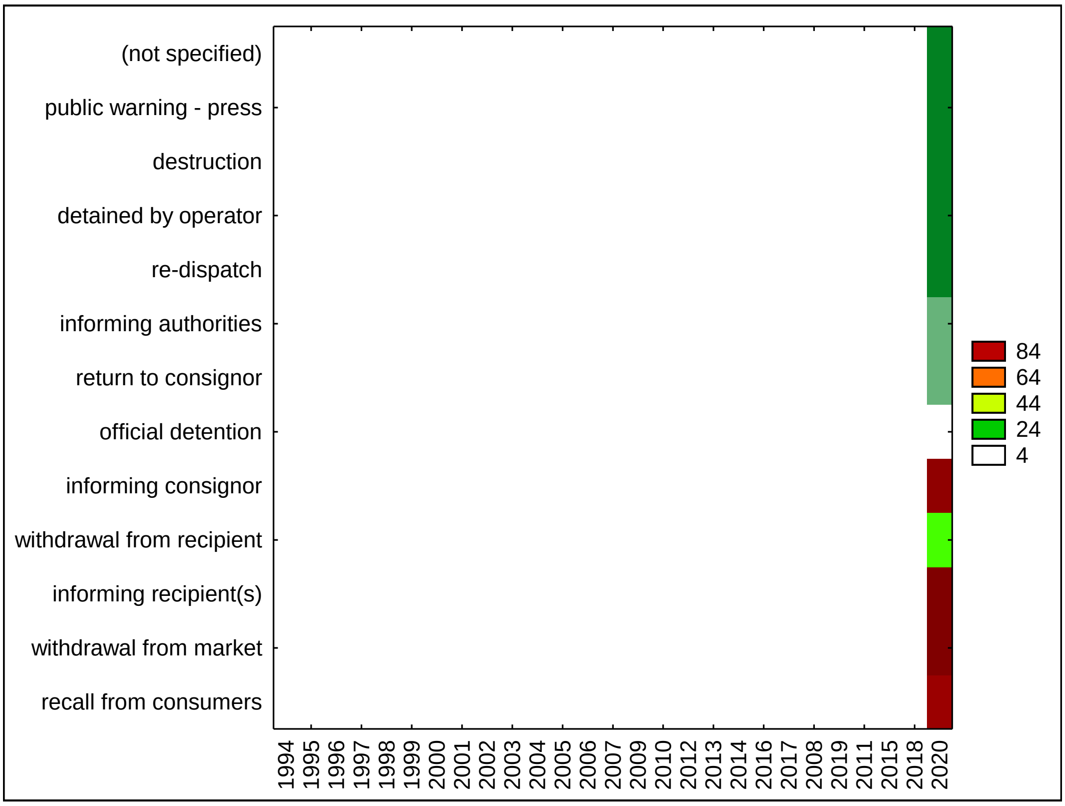

Figure S9g. Results of two-way joining cluster analysis for ethylene oxide (action taken)

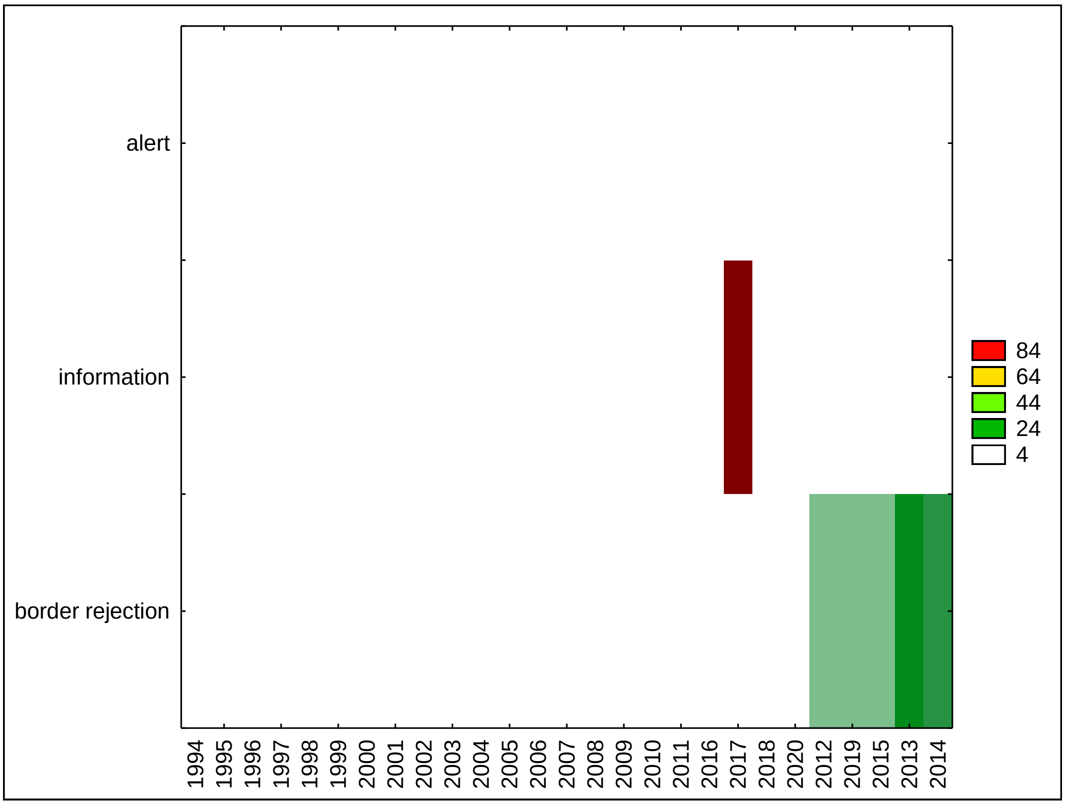

Figure S10a. Results of two-way joining cluster analysis for fipronil (notification type)

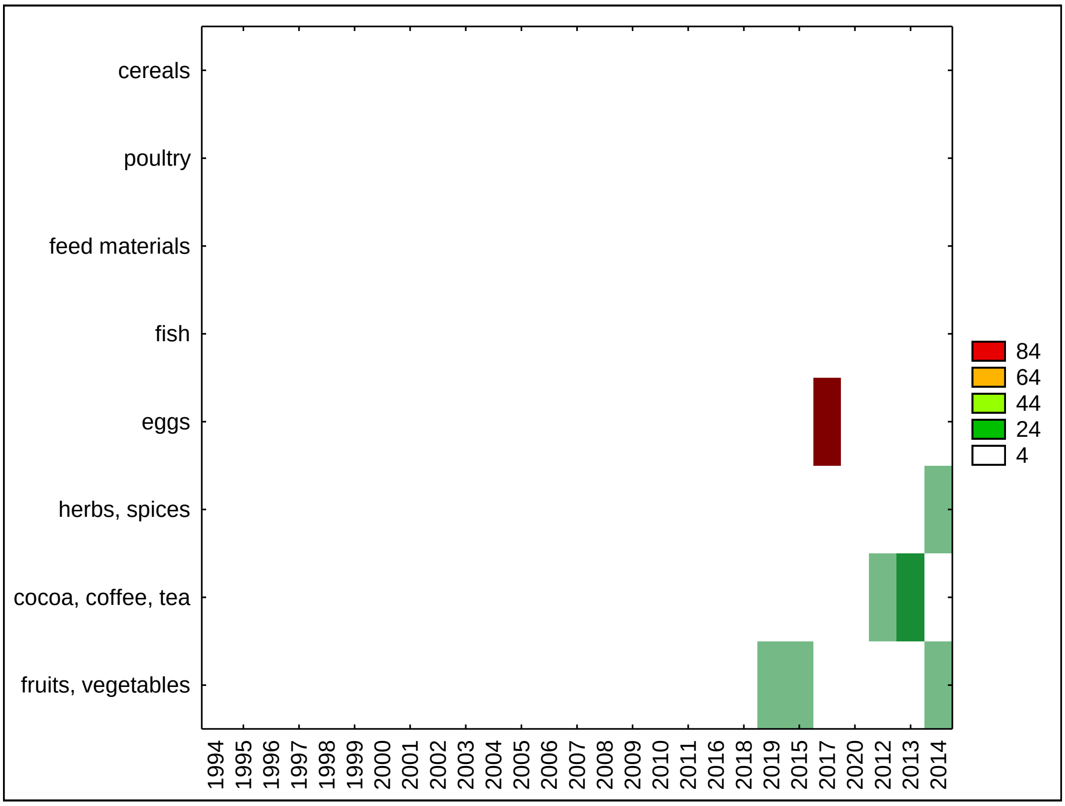

Figure S10b. Results of two-way joining cluster analysis for fipronil (product category)

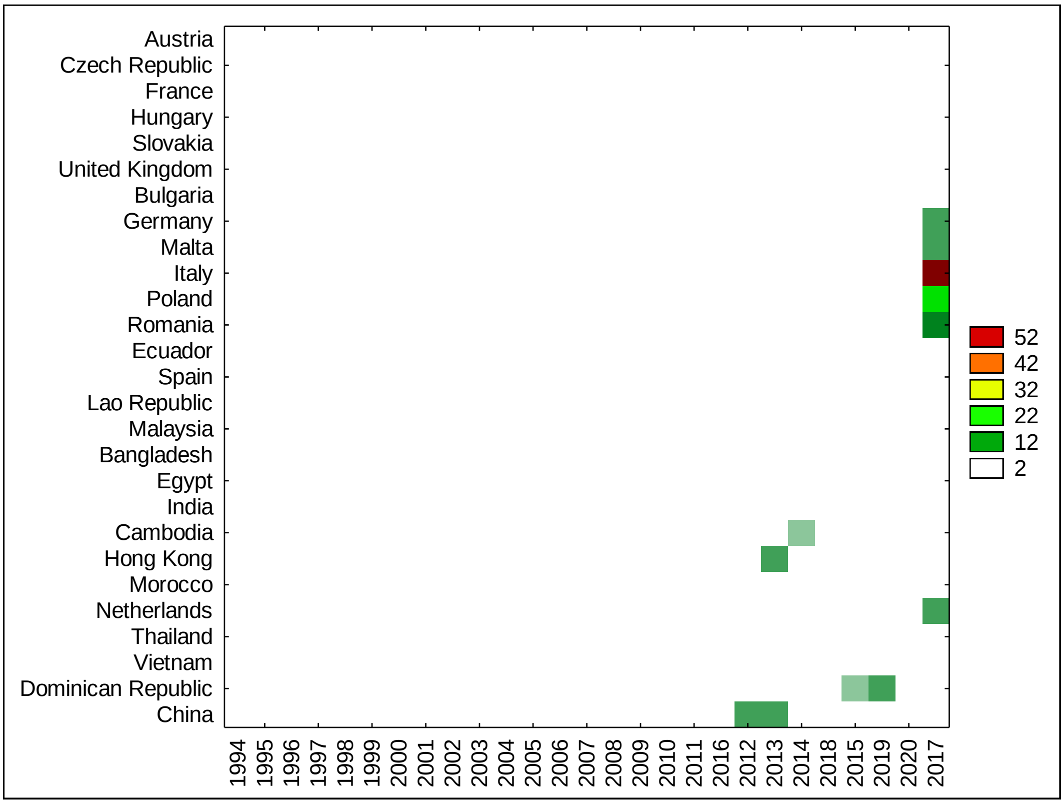

Figure S10c. Results of two-way joining cluster analysis for fipronil (origin country)

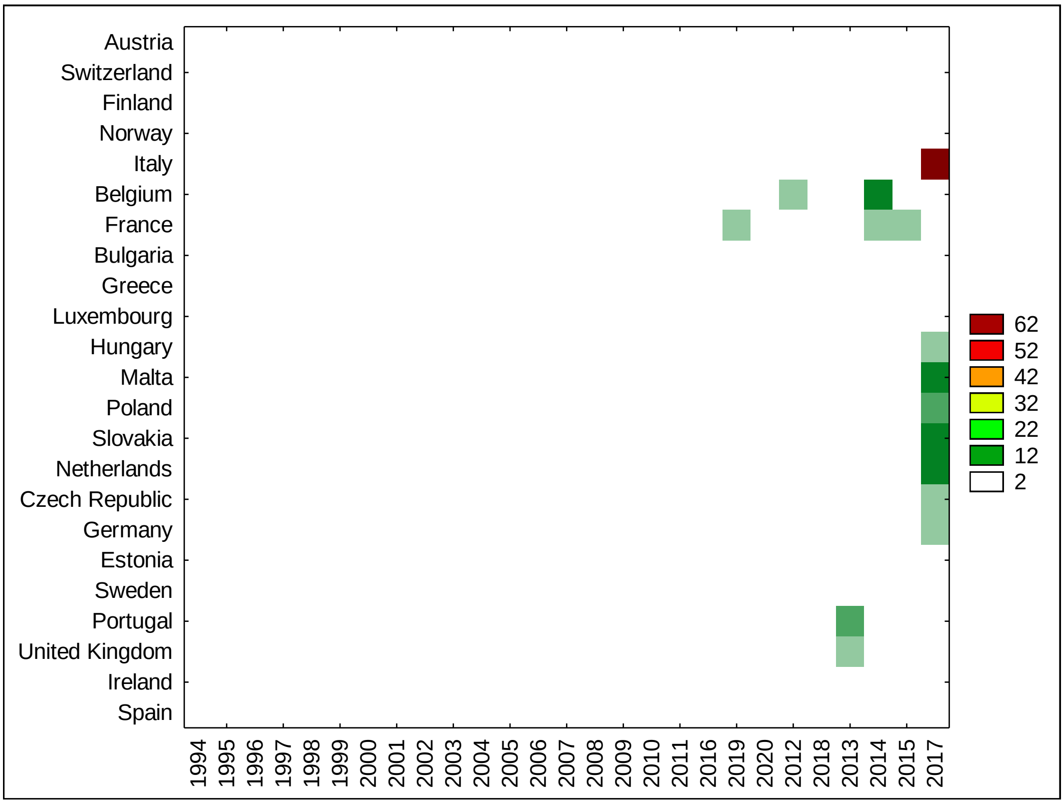

Figure S10d. Results of two-way joining cluster analysis for fipronil (notifying country)

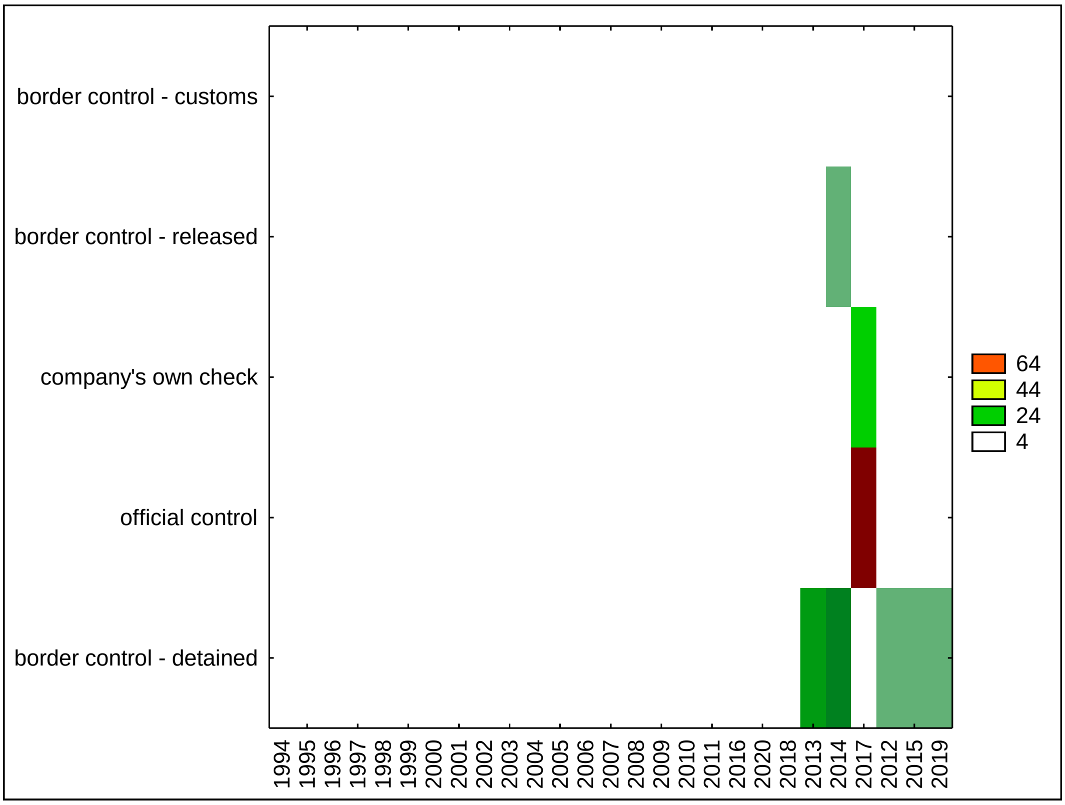

Figure S10e. Results of two-way joining cluster analysis for fipronil (notification basis)

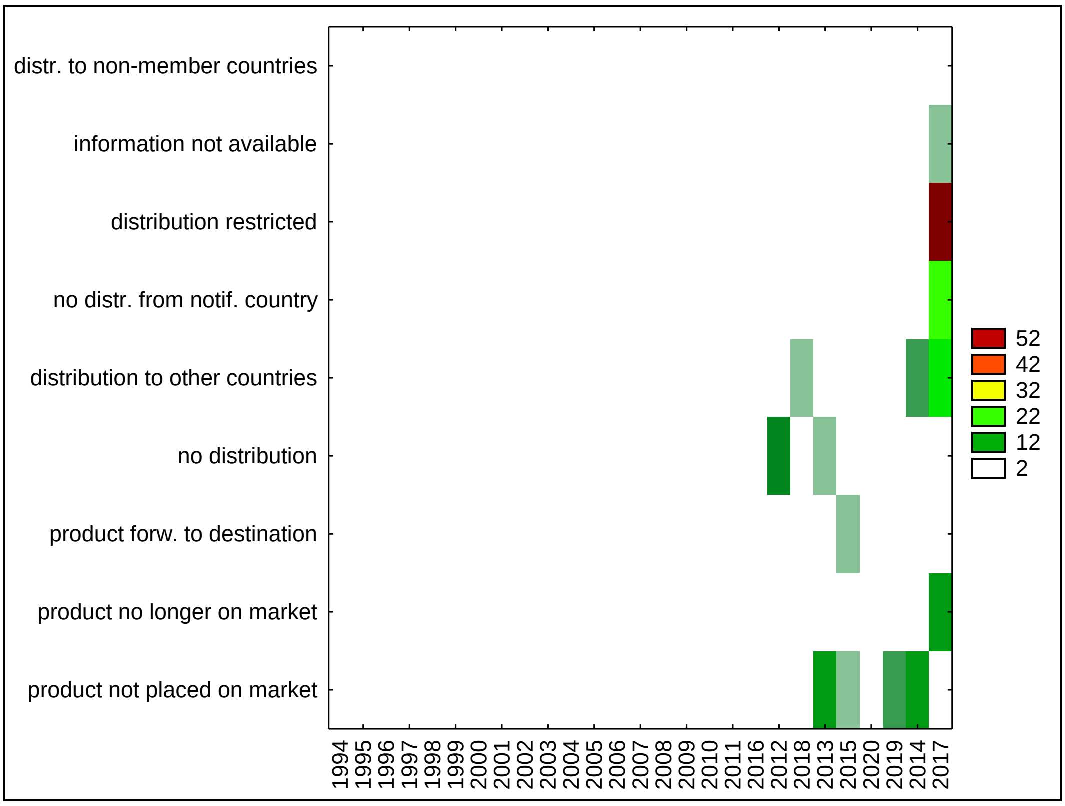

Figure S10f. Results of two-way joining cluster analysis for fipronil (distribution status)

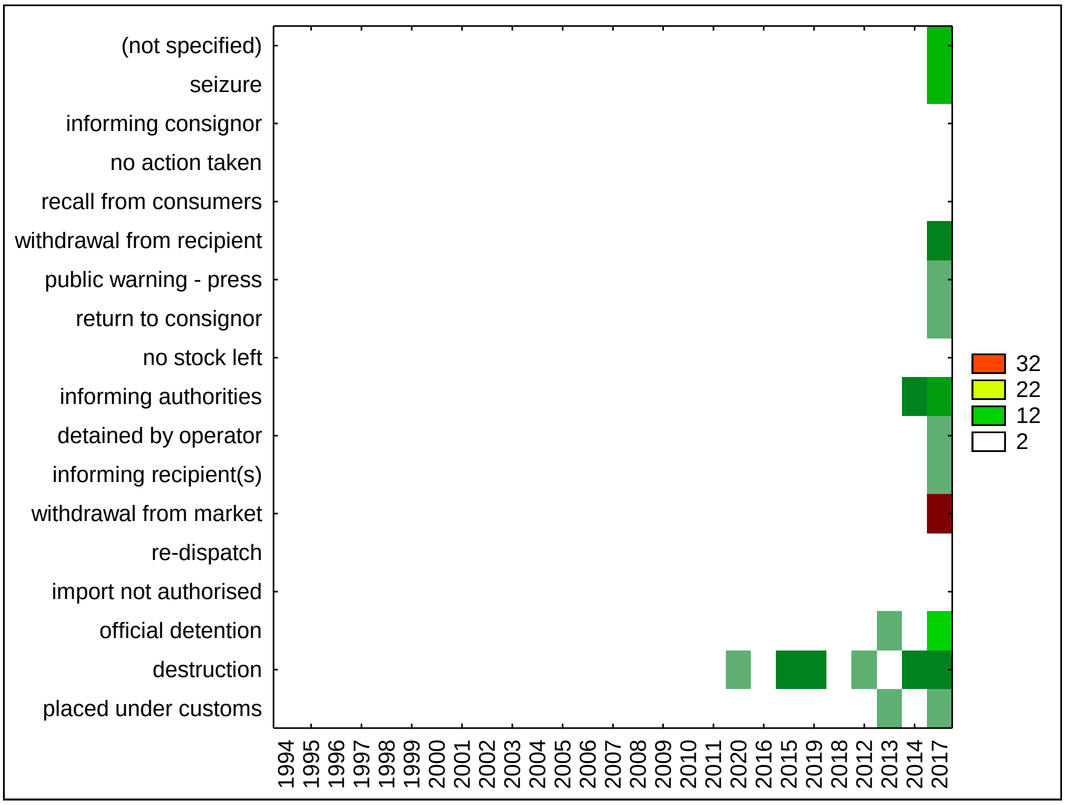

Figure S10g. Results of two-way joining cluster analysis for fipronil (action taken)

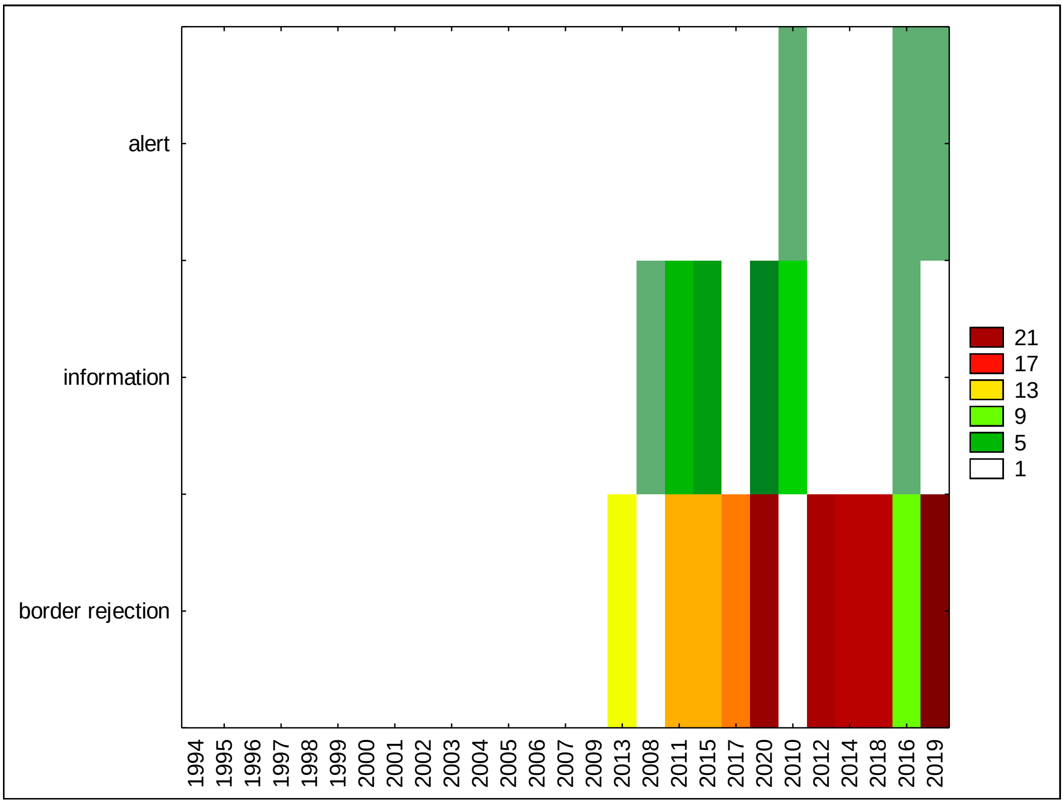

Figure S11a. Results of two-way joining cluster analysis for formetanate (notification type)

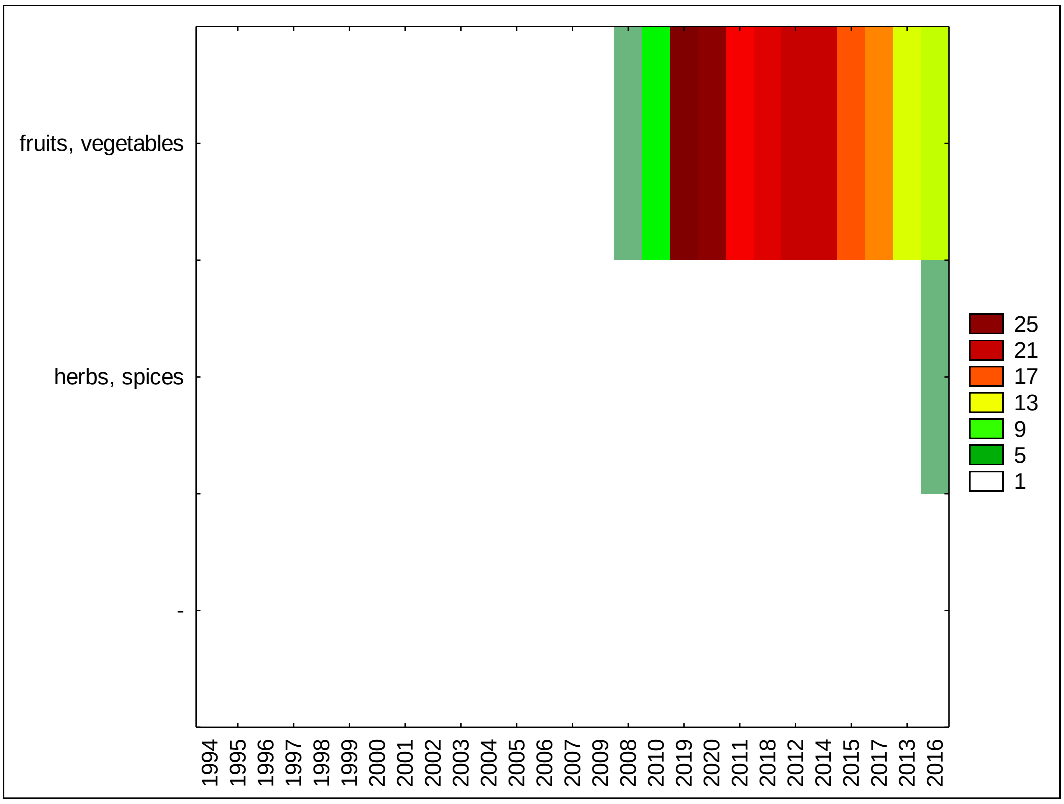

Figure S11b. Results of two-way joining cluster analysis for formetanate (product category)

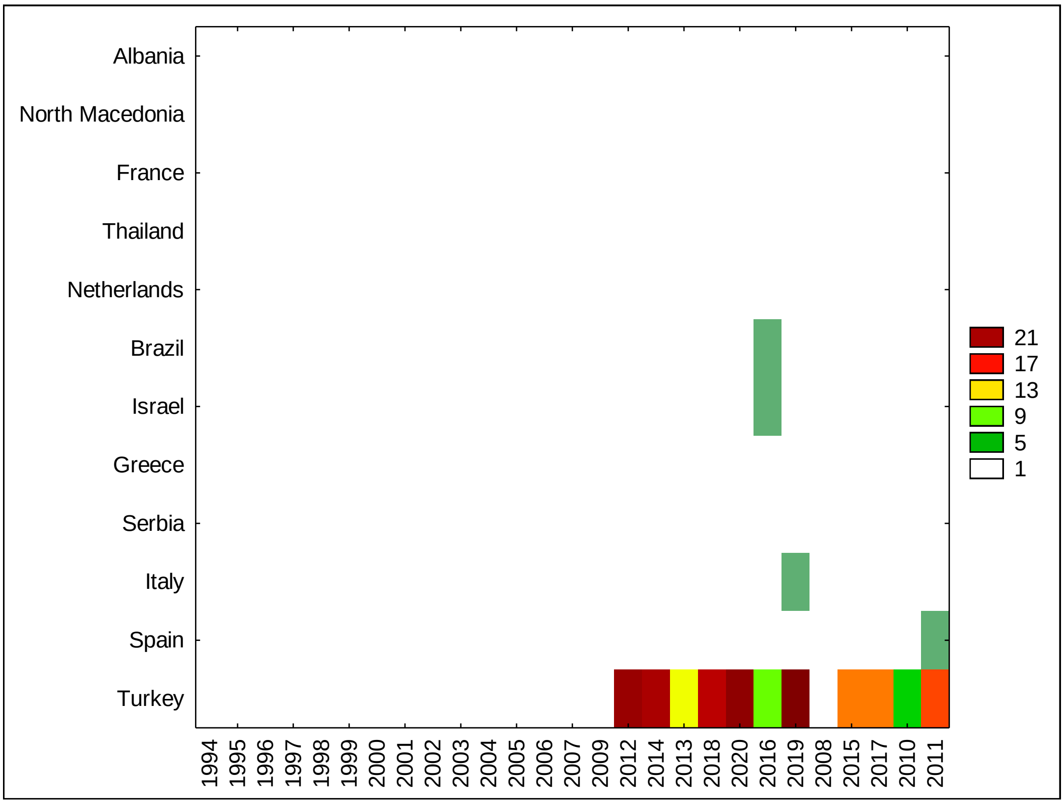

Figure S11c. Results of two-way joining cluster analysis for formetanate (origin country)

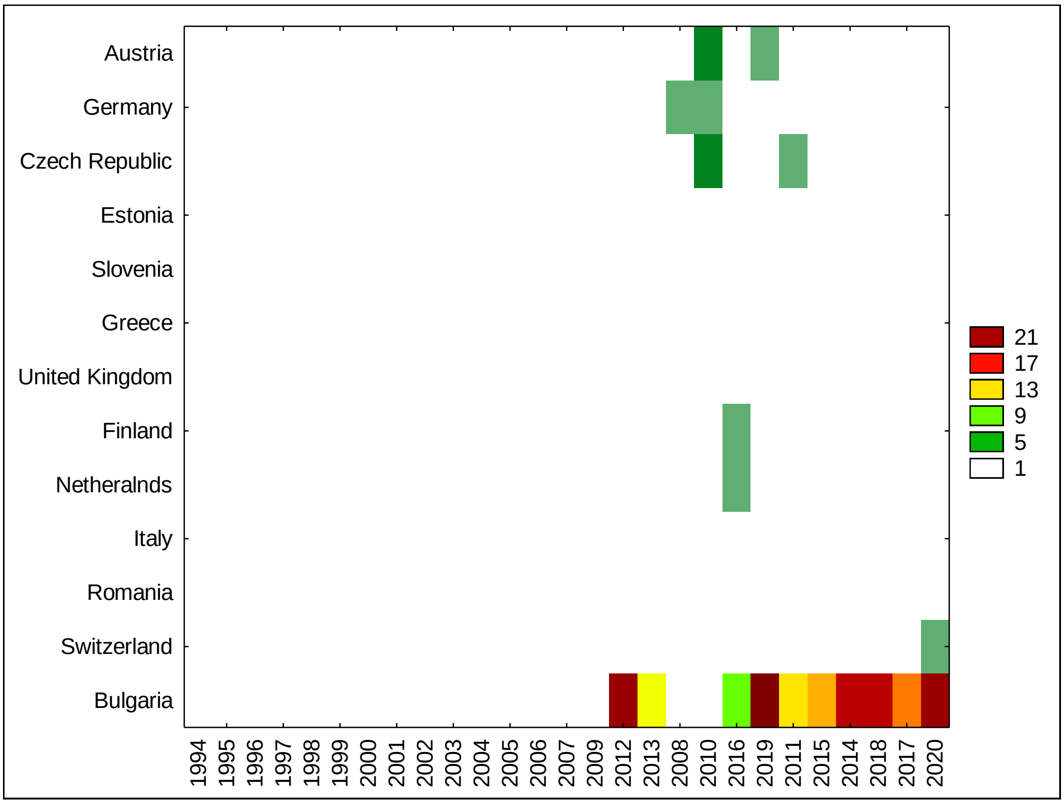

Figure S11d. Results of two-way joining cluster analysis for formetanate (notifying country)

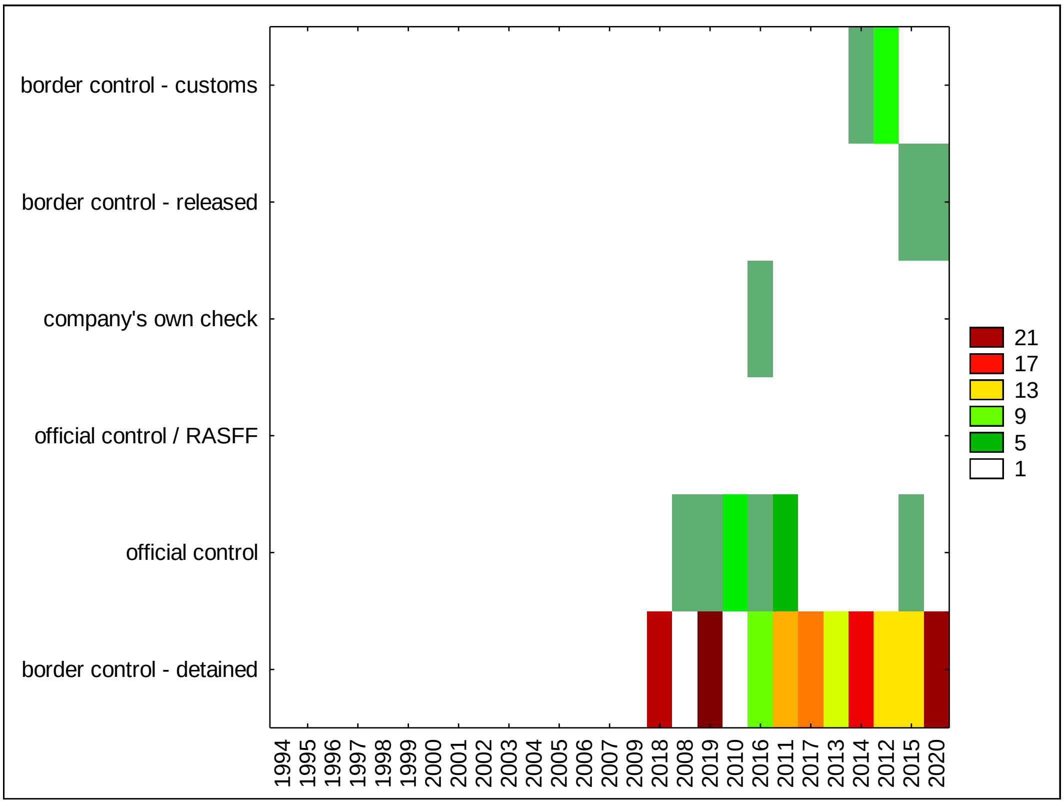

Figure S11e. Results of two-way joining cluster analysis for formetanate (notification basis)

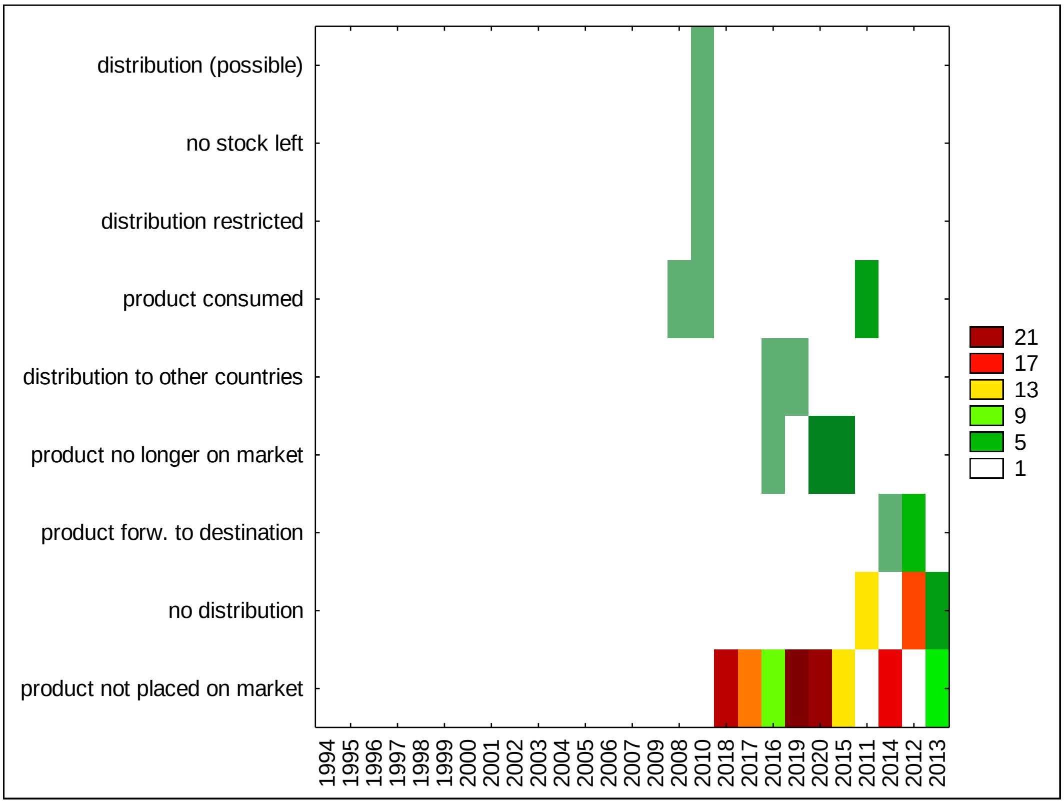

Figure S11f. Results of two-way joining cluster analysis for formetanate (distribution status)

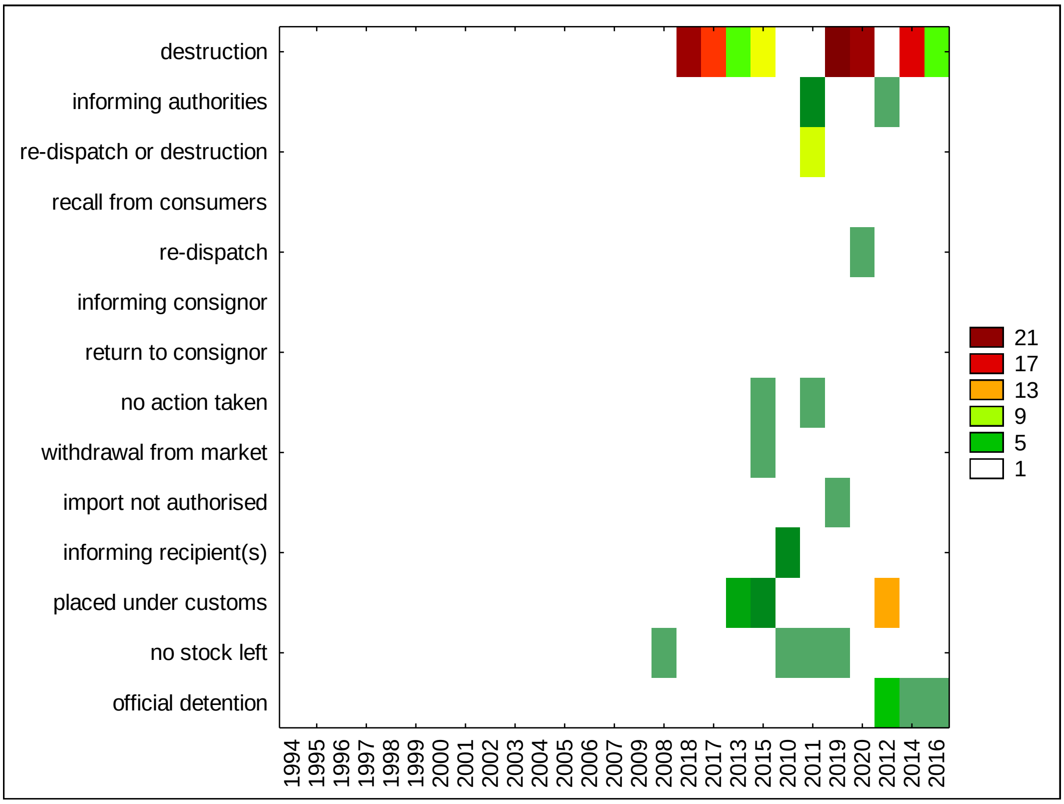

Figure S11g. Results of two-way joining cluster analysis for formetanate (action taken)

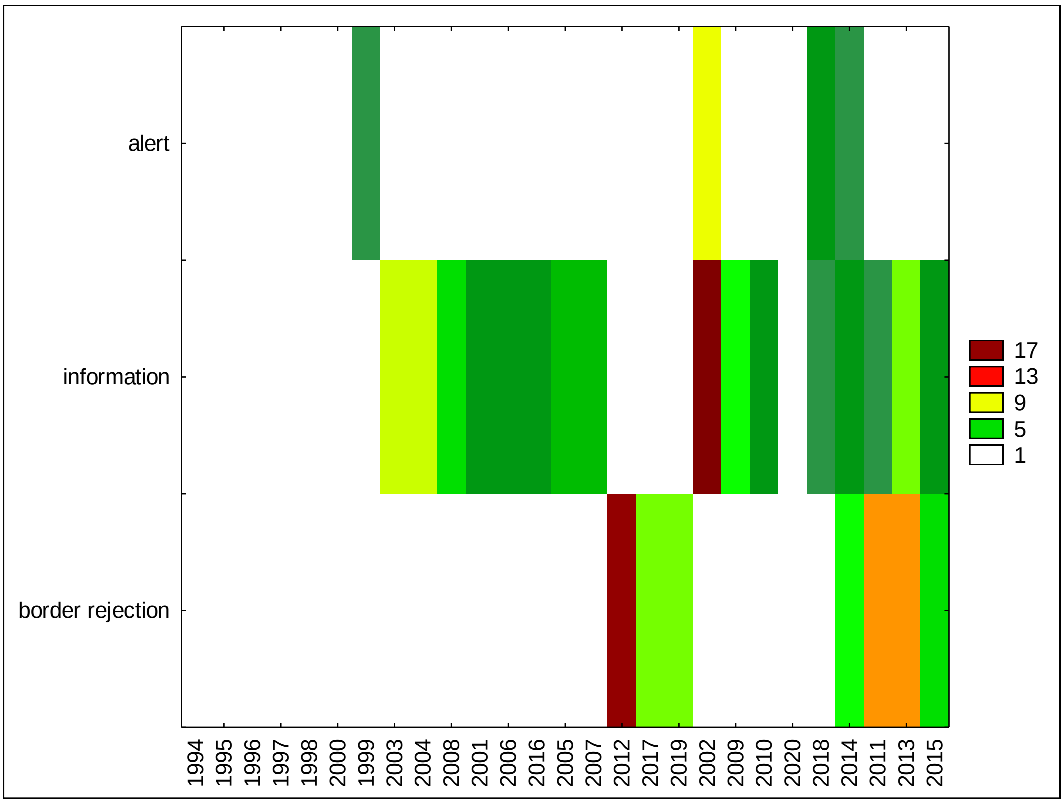

Figure S12a. Results of two-way joining cluster analysis for methamidophos (notification type)

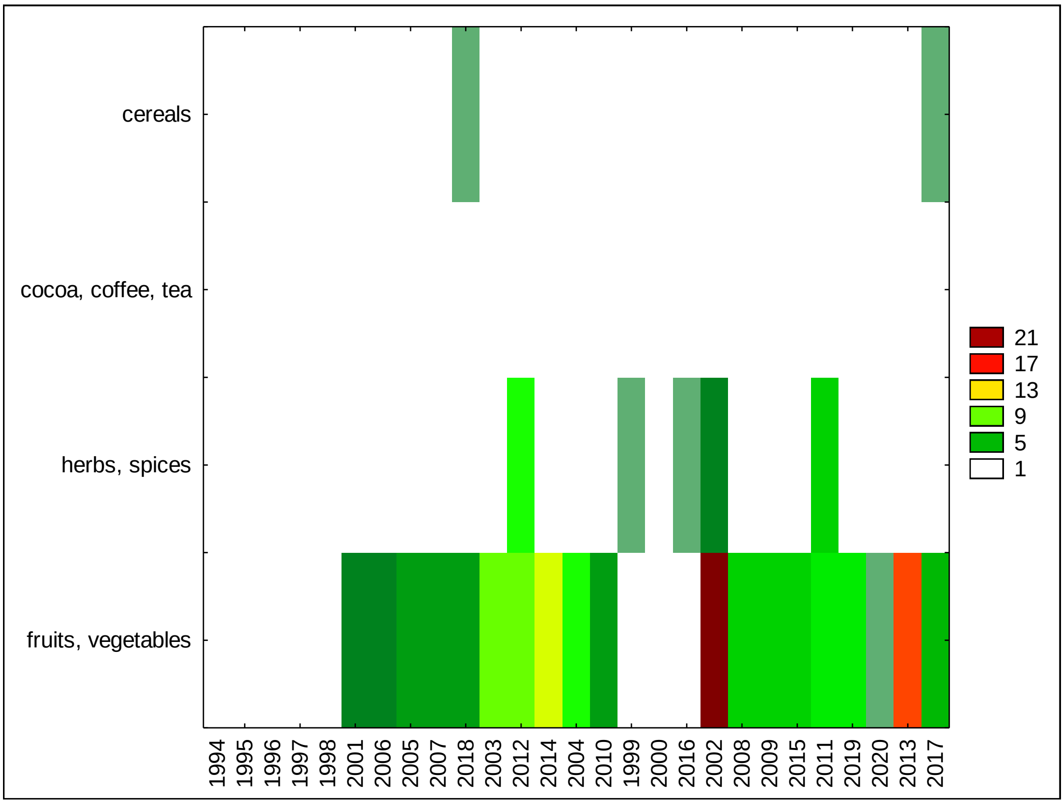

Figure S12b. Results of two-way joining cluster analysis for methamidophos (product category)

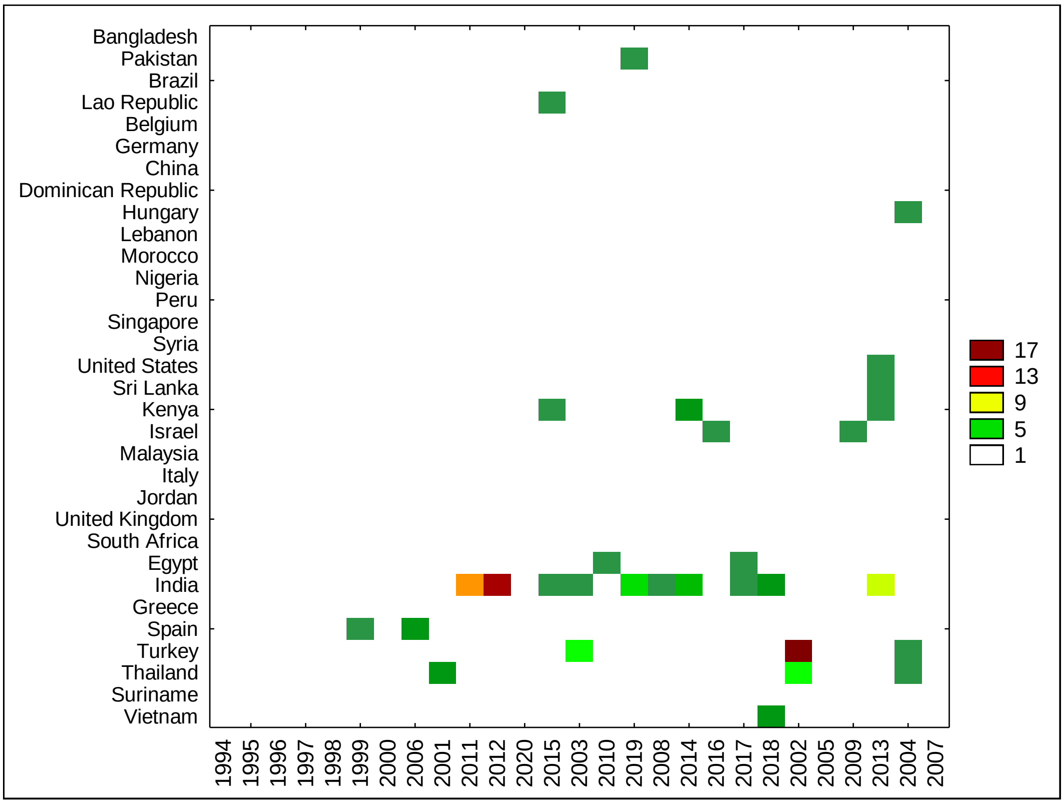

Figure S12c. Results of two-way joining cluster analysis for methamidophos (origin country)

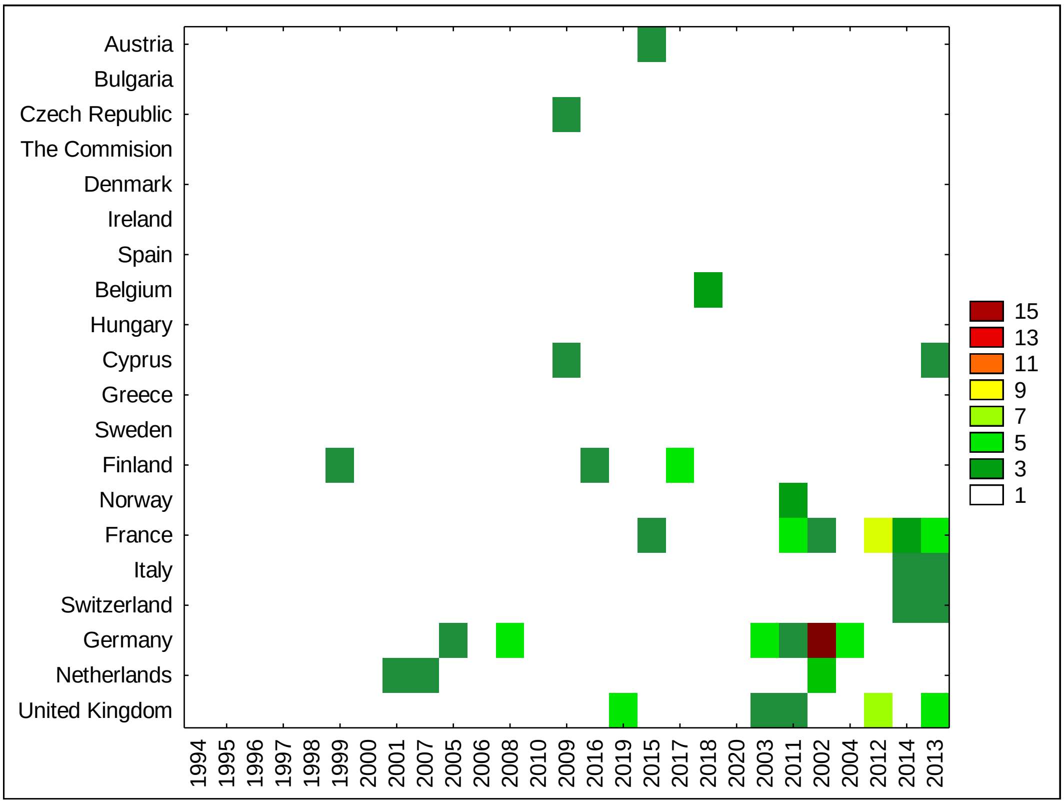

Figure S12d. Results of two-way joining cluster analysis for methamidophos (notifying country)

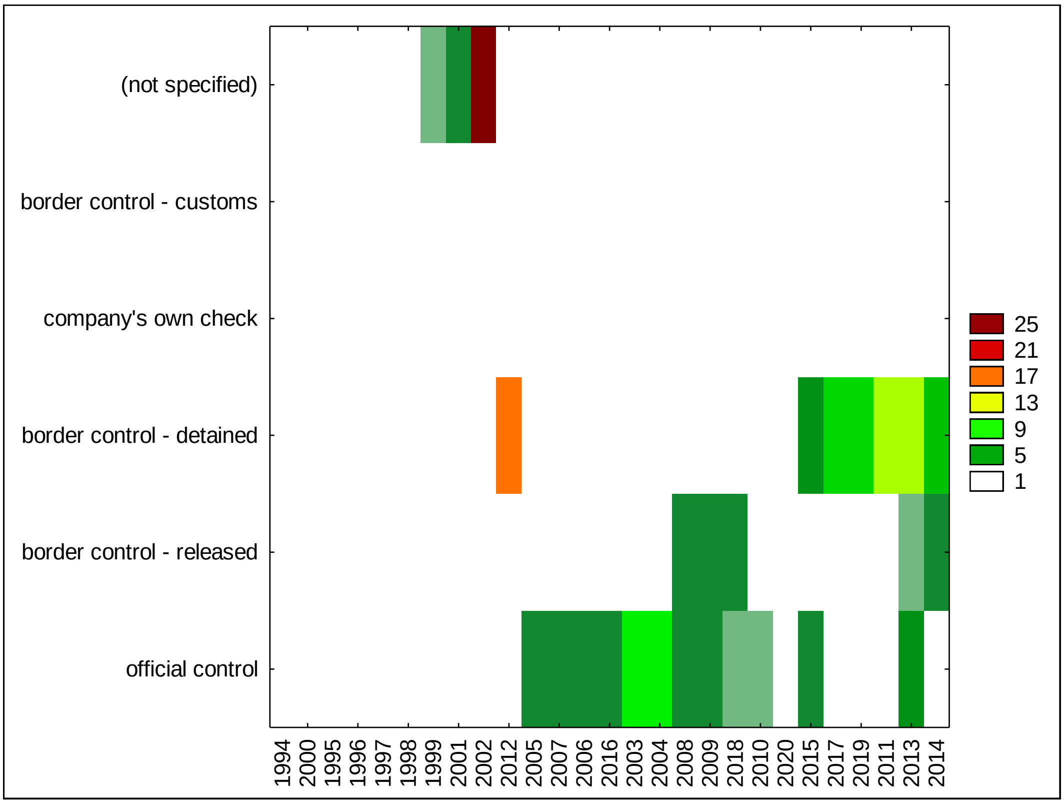

Figure S12e. Results of two-way joining cluster analysis for methamidophos (notification basis)

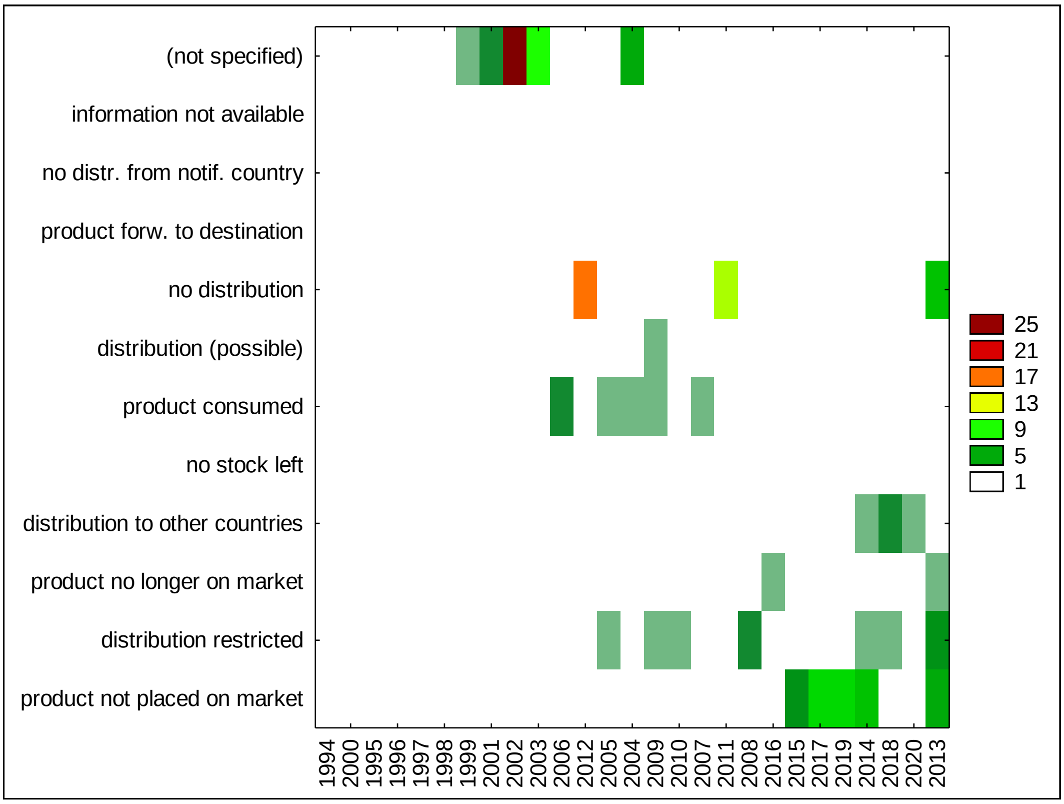

Figure S12f. Results of two-way joining cluster analysis for methamidophos (distribution status)

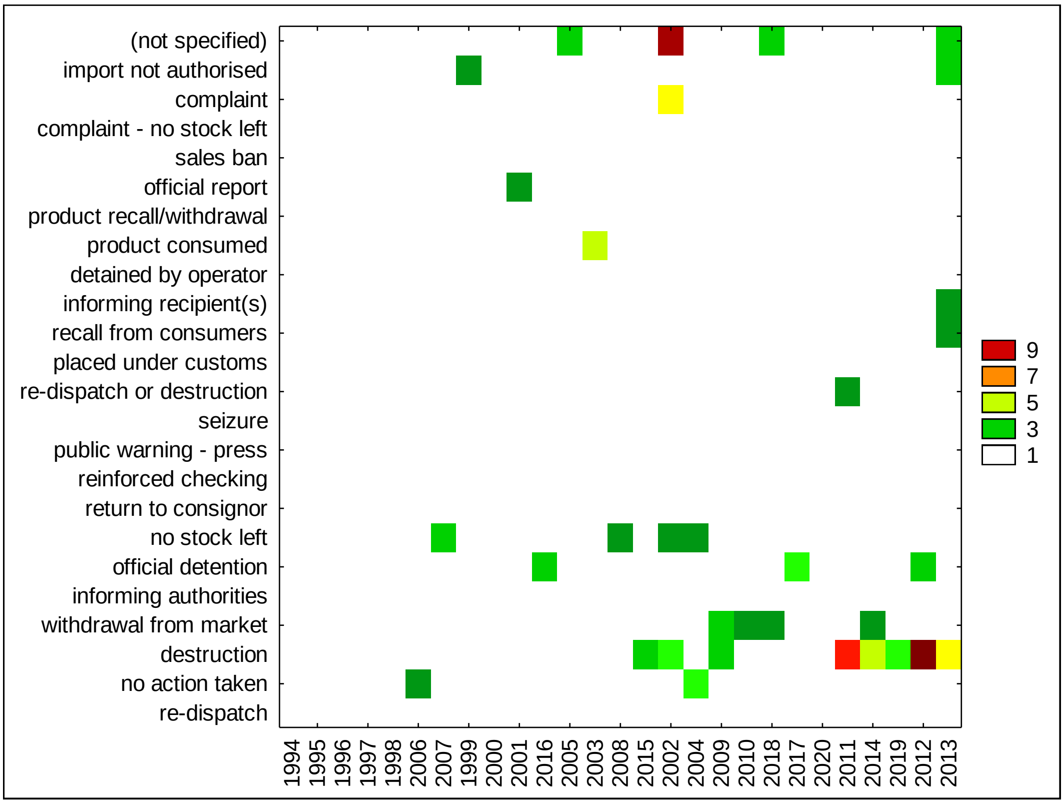

Figure S12g. Results of two-way joining cluster analysis for methamidophos (action taken)

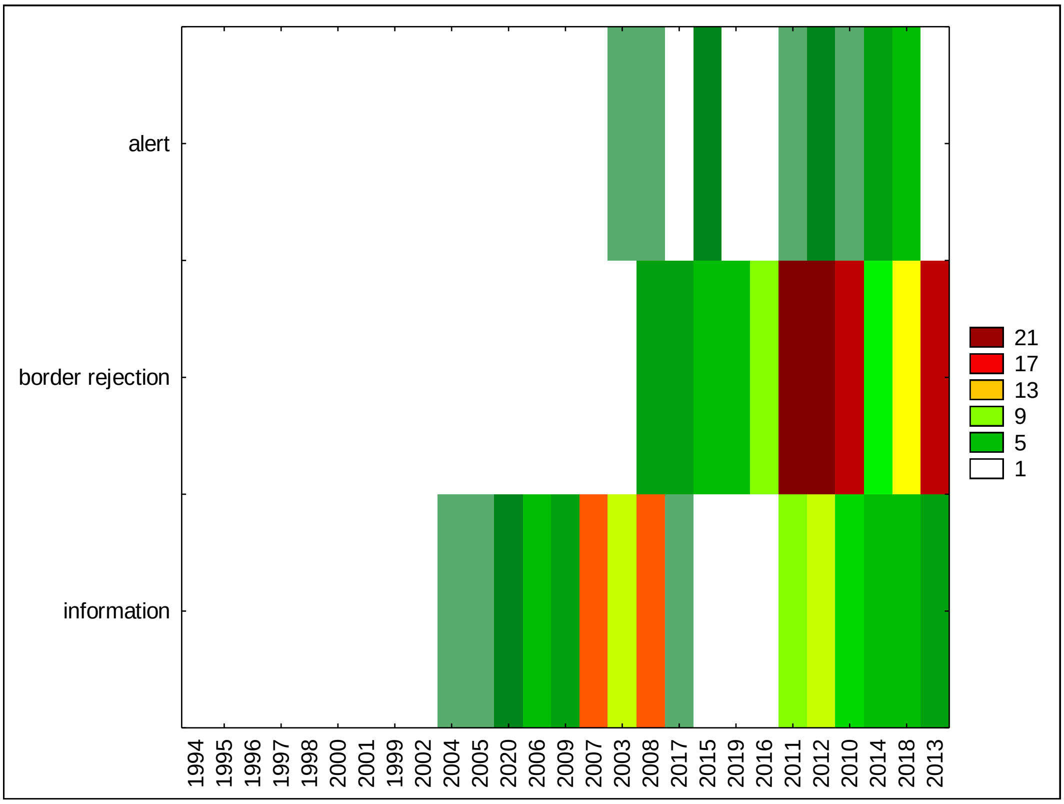

Figure S13a. Results of two-way joining cluster analysis for methomyl (notification type)

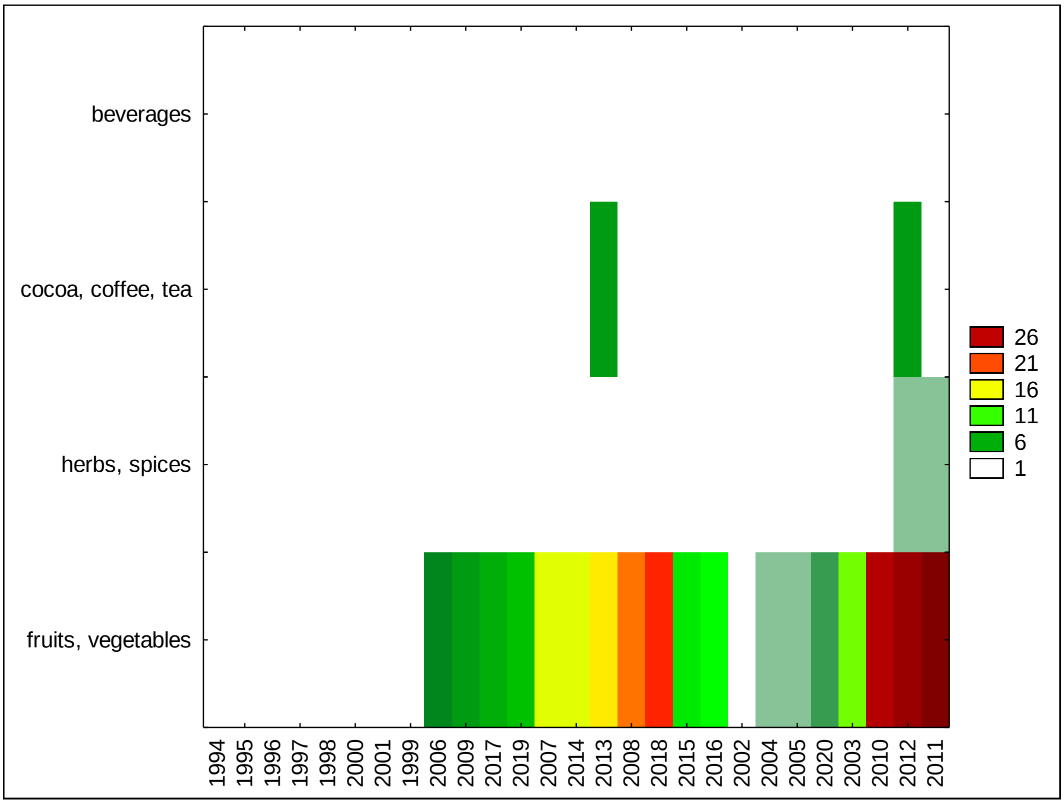

Figure S13b. Results of two-way joining cluster analysis for methomyl (product category)

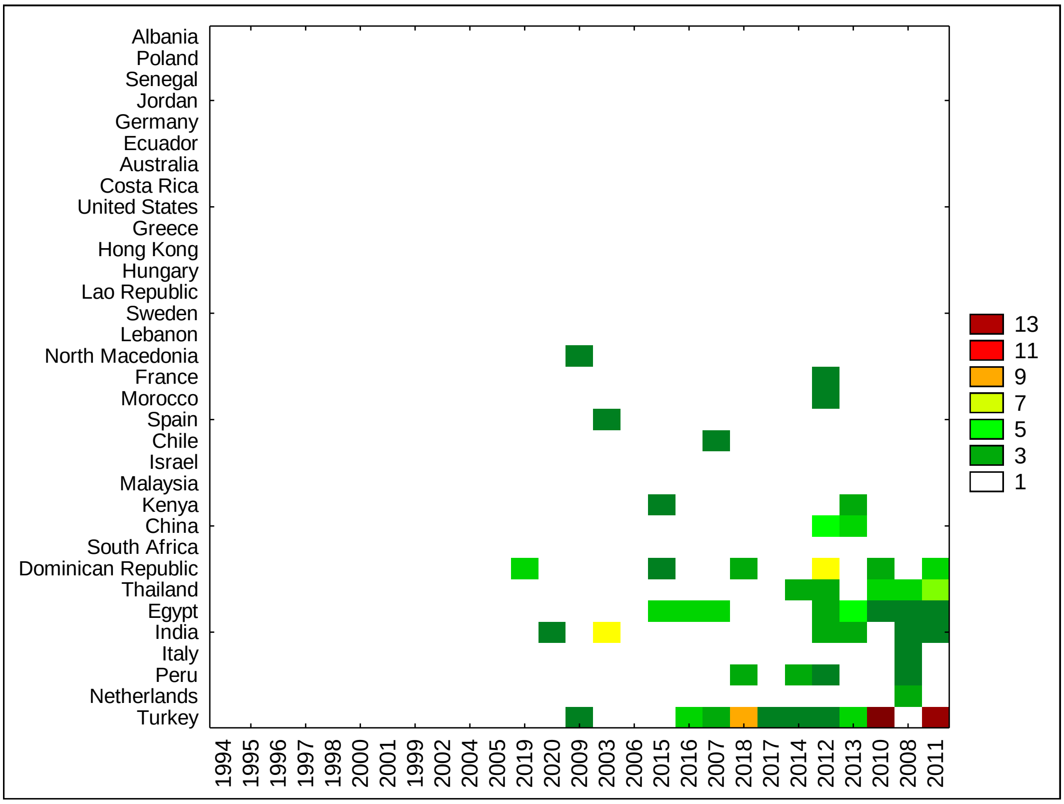

Figure S13c. Results of two-way joining cluster analysis for methomyl (origin country)

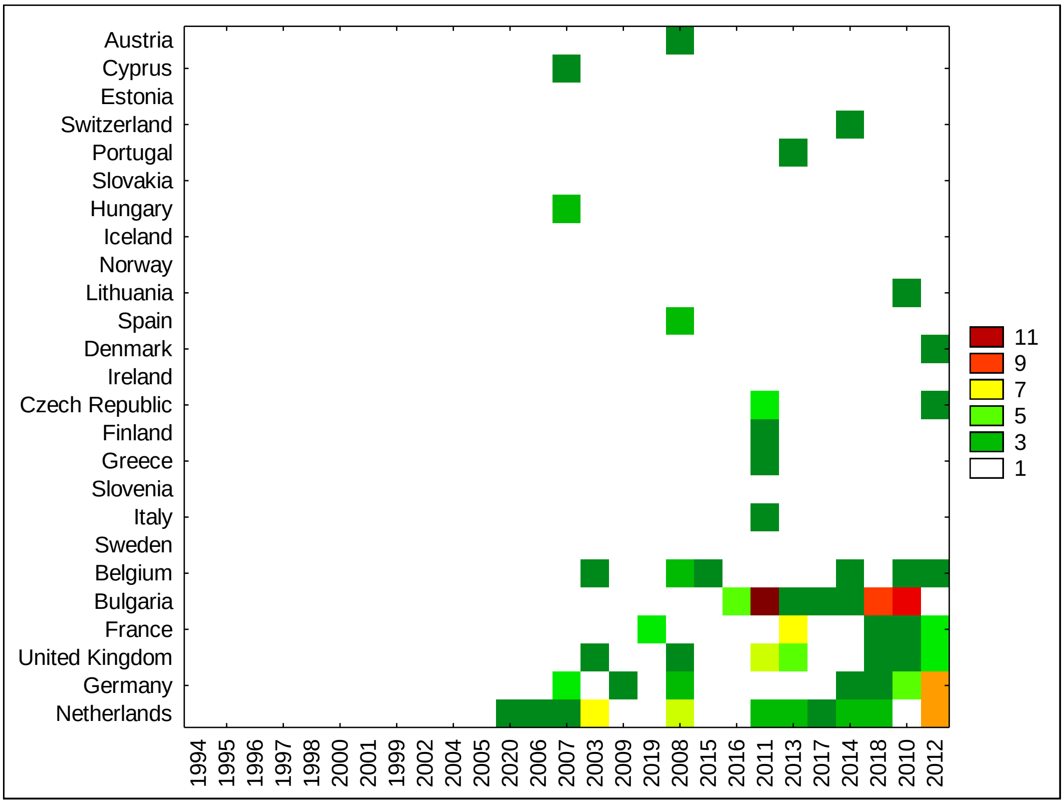

Figure S13d. Results of two-way joining cluster analysis for methomyl (notifying country)

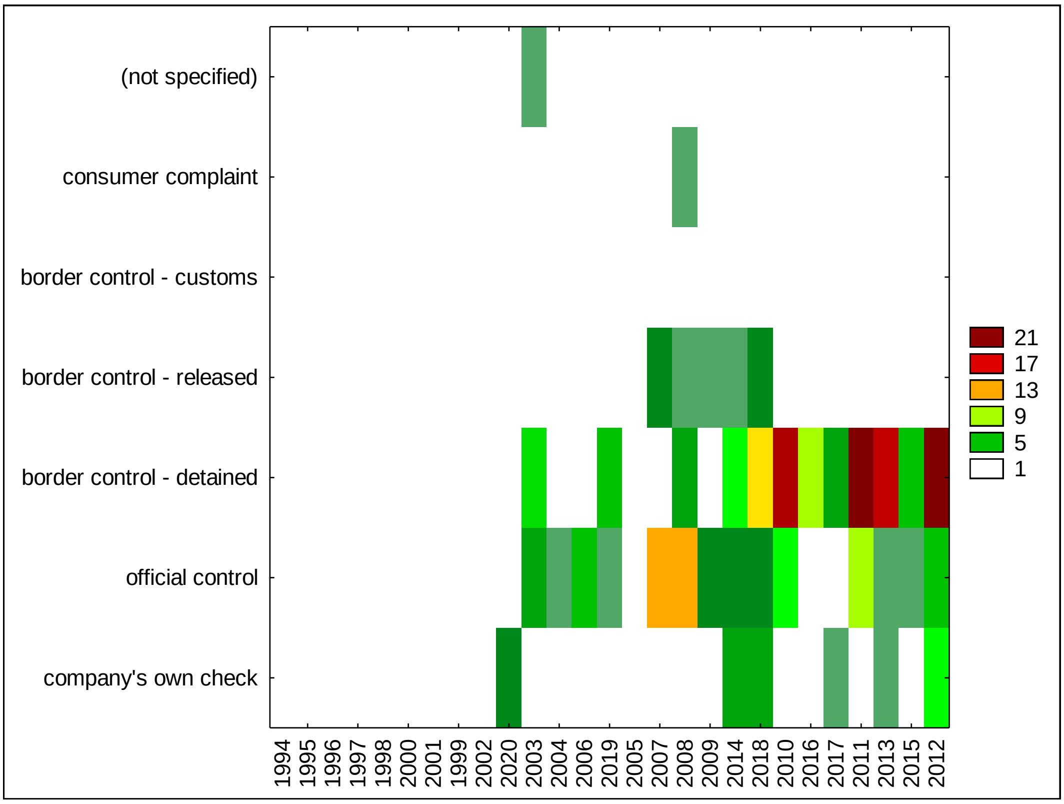

Figure S13e. Results of two-way joining cluster analysis for methomyl (notification basis)

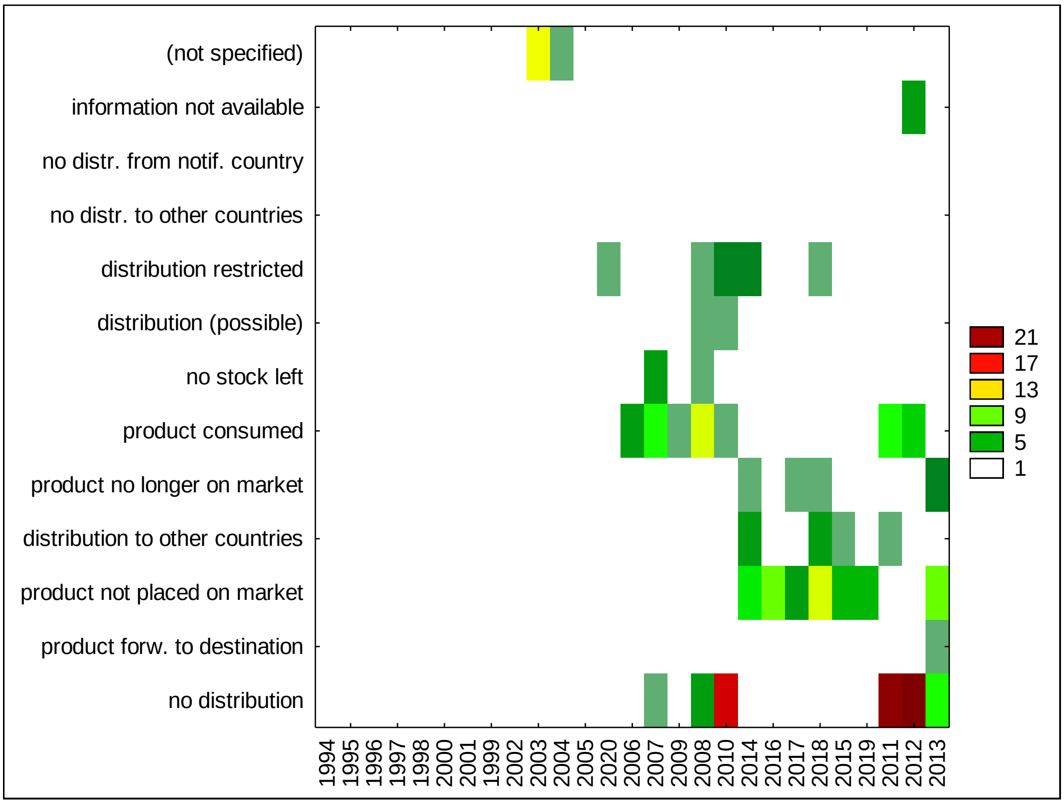

Figure S13f. Results of two-way joining cluster analysis for methomyl (distribution status)

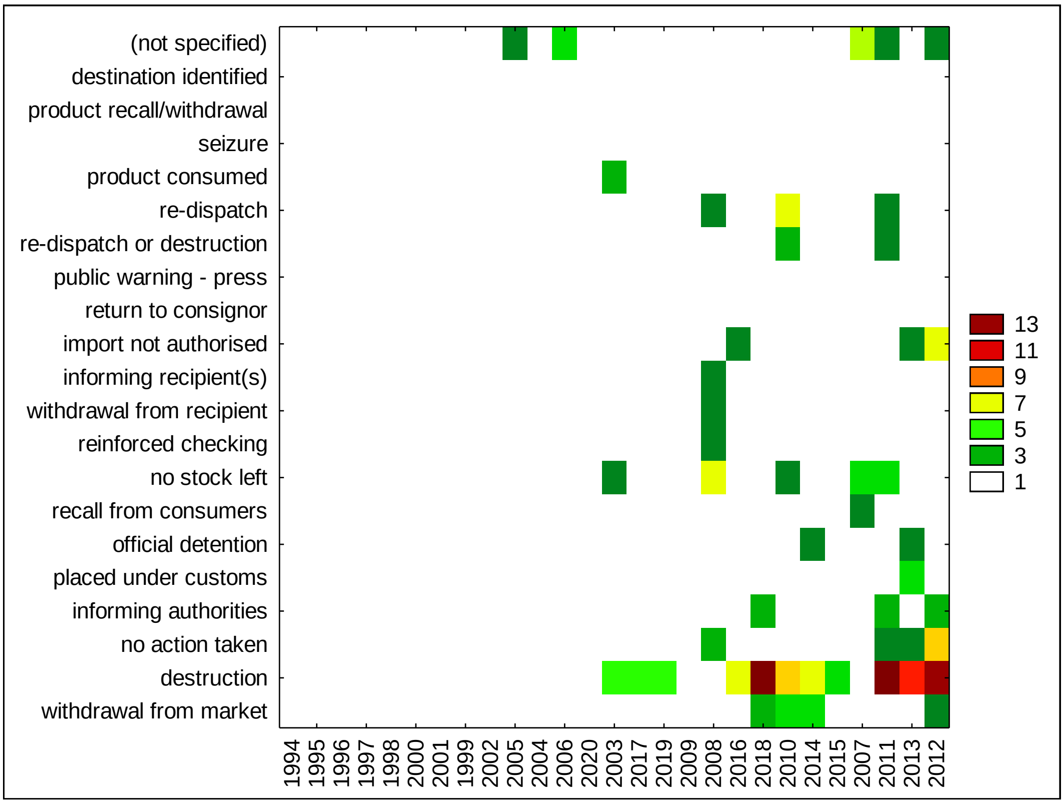

Figure S13g. Results of two-way joining cluster analysis for methomyl (action taken)

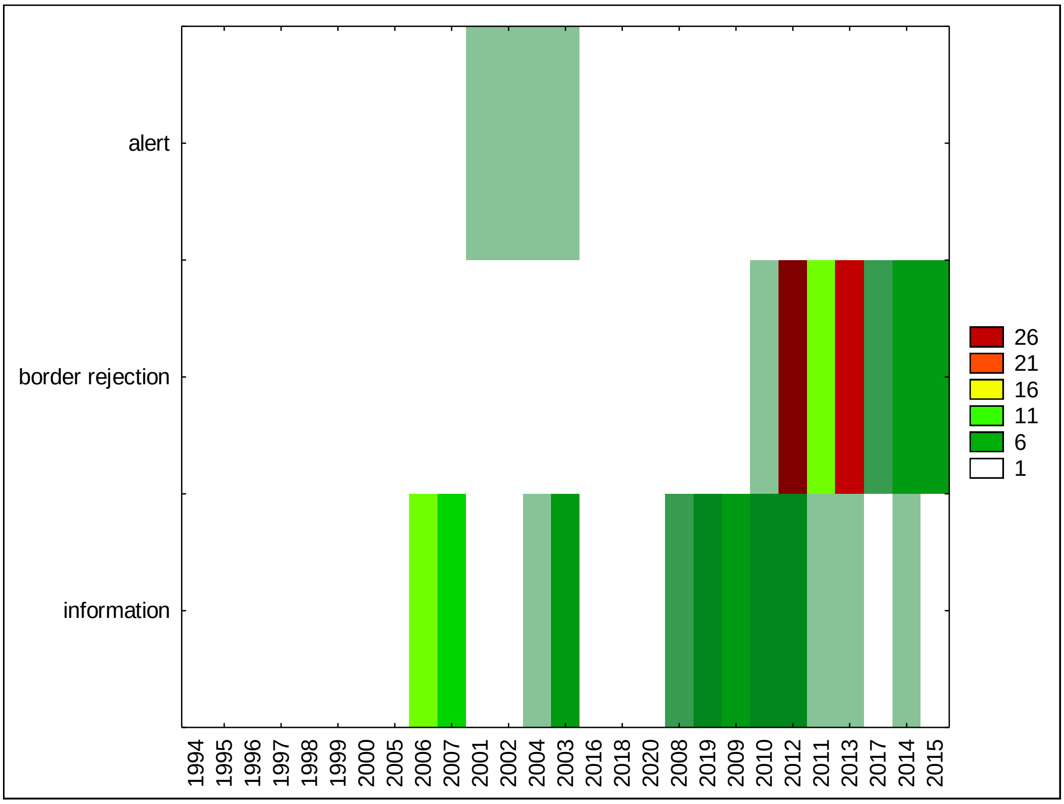

Figure S14a. Results of two-way joining cluster analysis for monocrotophos (notification type)

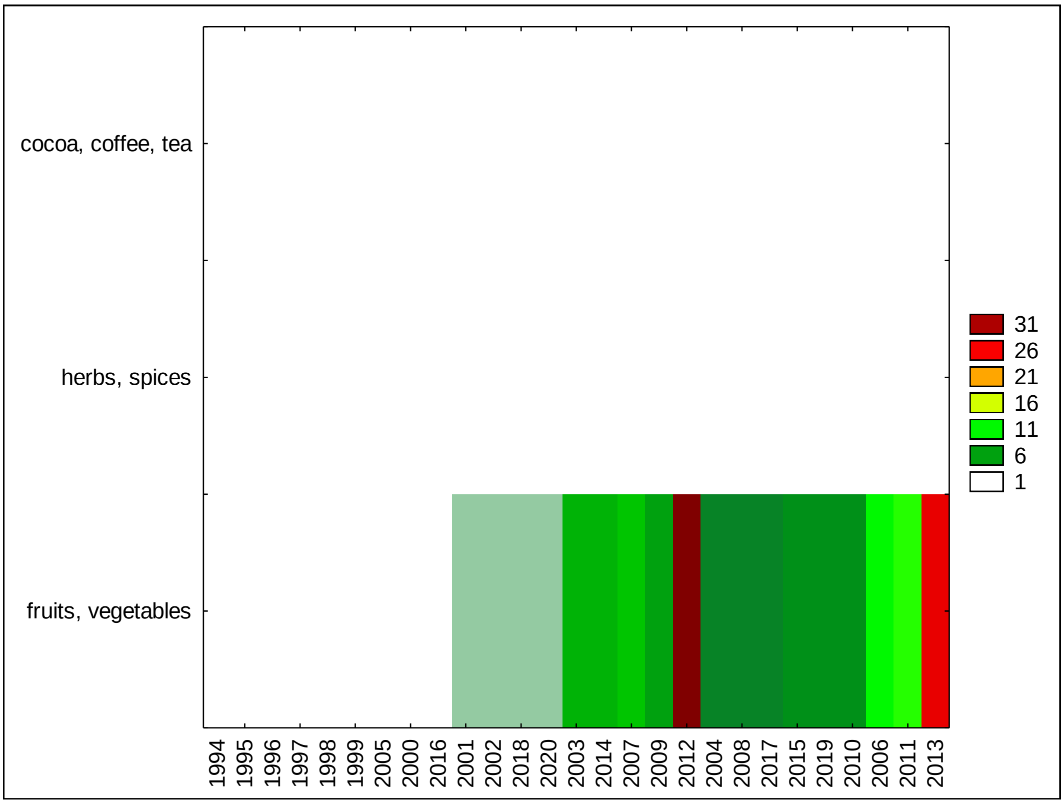

Figure S14b. Results of two-way joining cluster analysis for monocrotophos (product category)

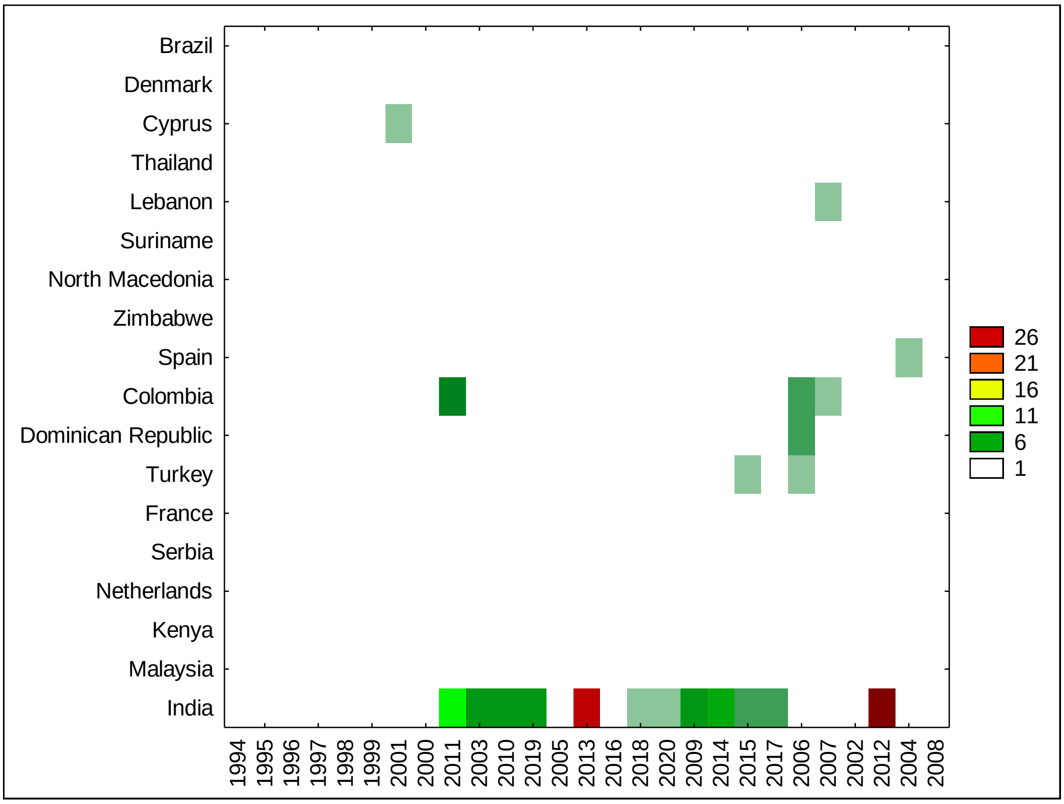

Figure S14c. Results of two-way joining cluster analysis for monocrotophos (origin country)

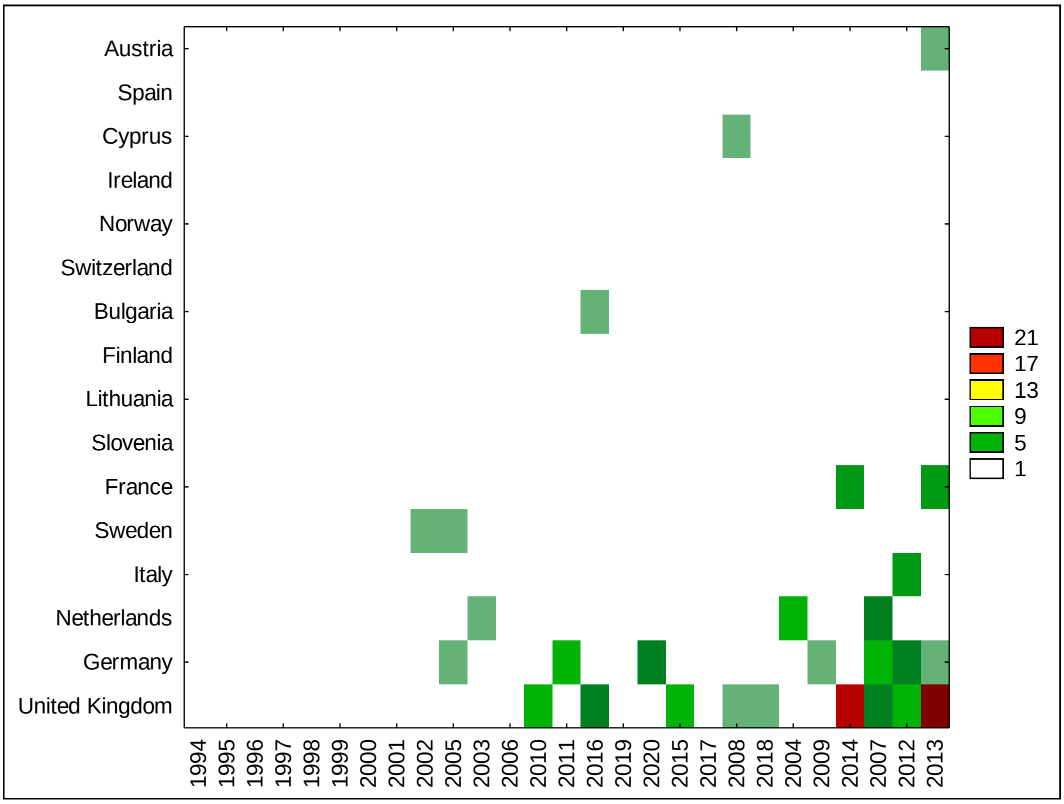

Figure S14d. Results of two-way joining cluster analysis for monocrotophos (notifying country)

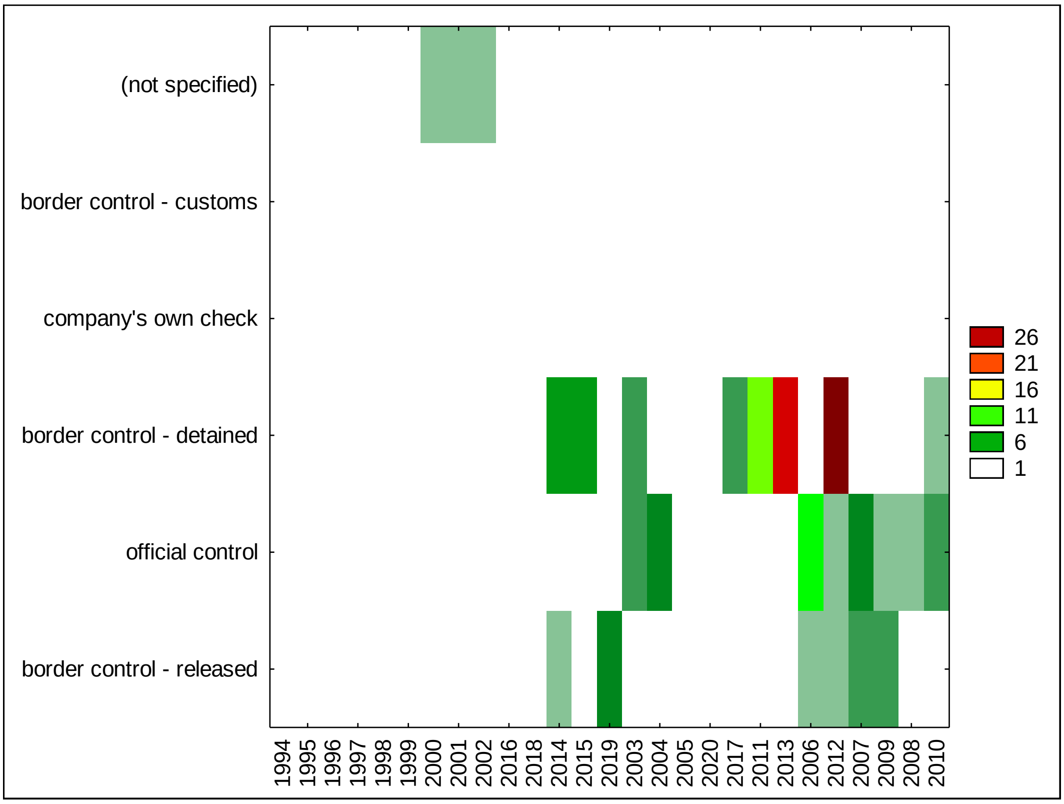

Figure S14e. Results of two-way joining cluster analysis for monocrotophos (notification basis)

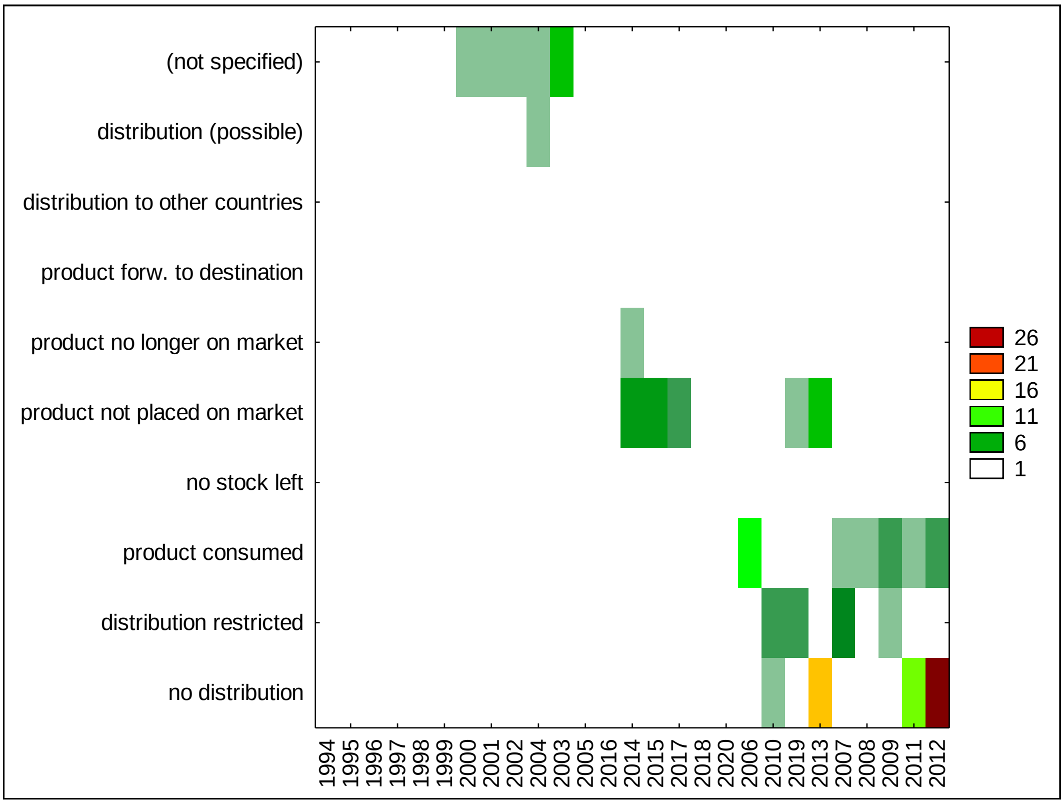

Figure S14f. Results of two-way joining cluster analysis for monocrotophos (distribution status)

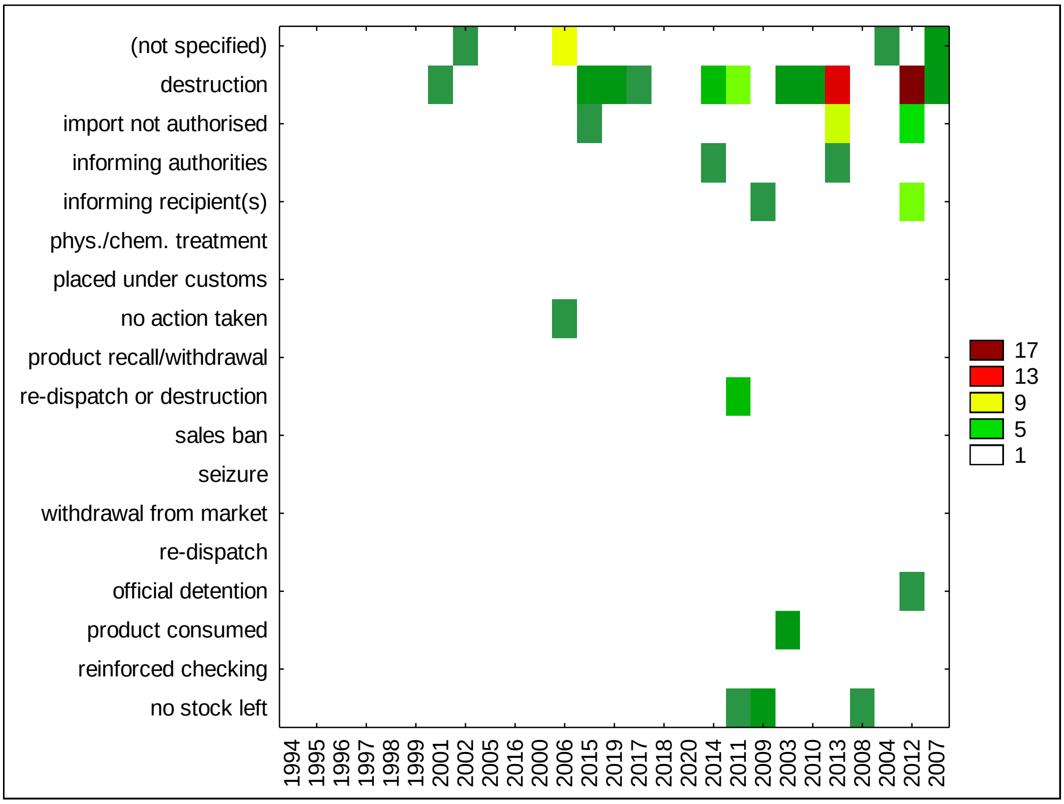

Figure S14g. Results of two-way joining cluster analysis for monocrotophos (action taken)

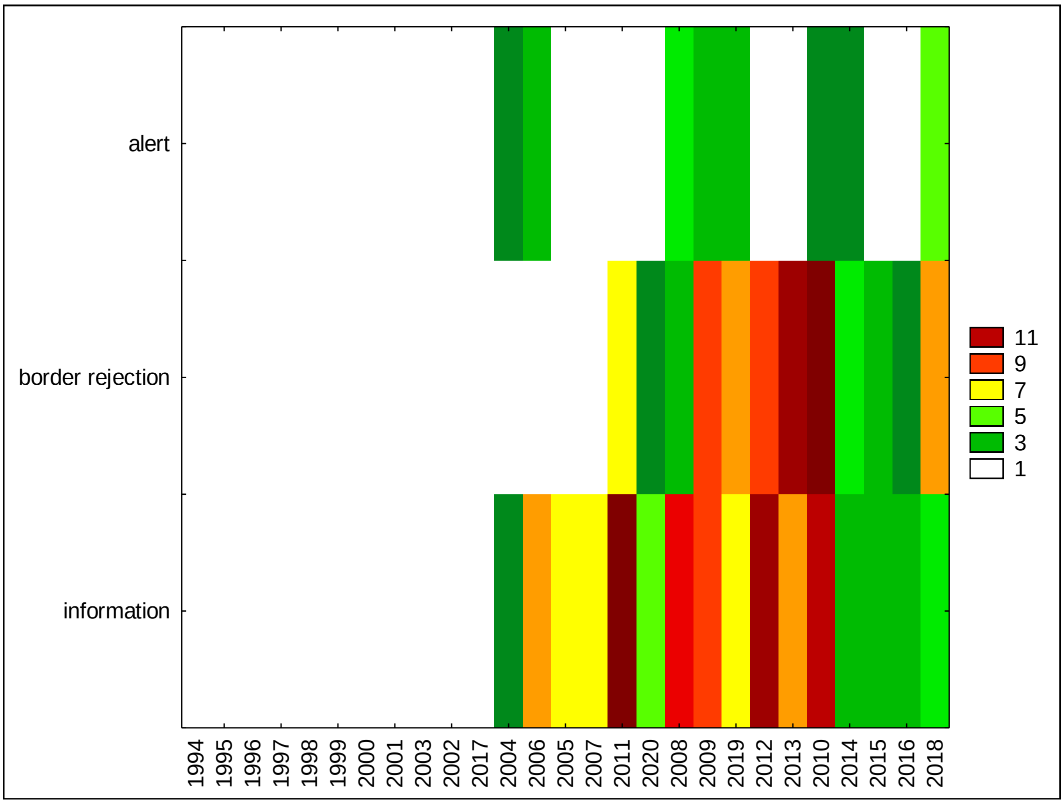

Figure S15a. Results of two-way joining cluster analysis for omethoate (notification type)

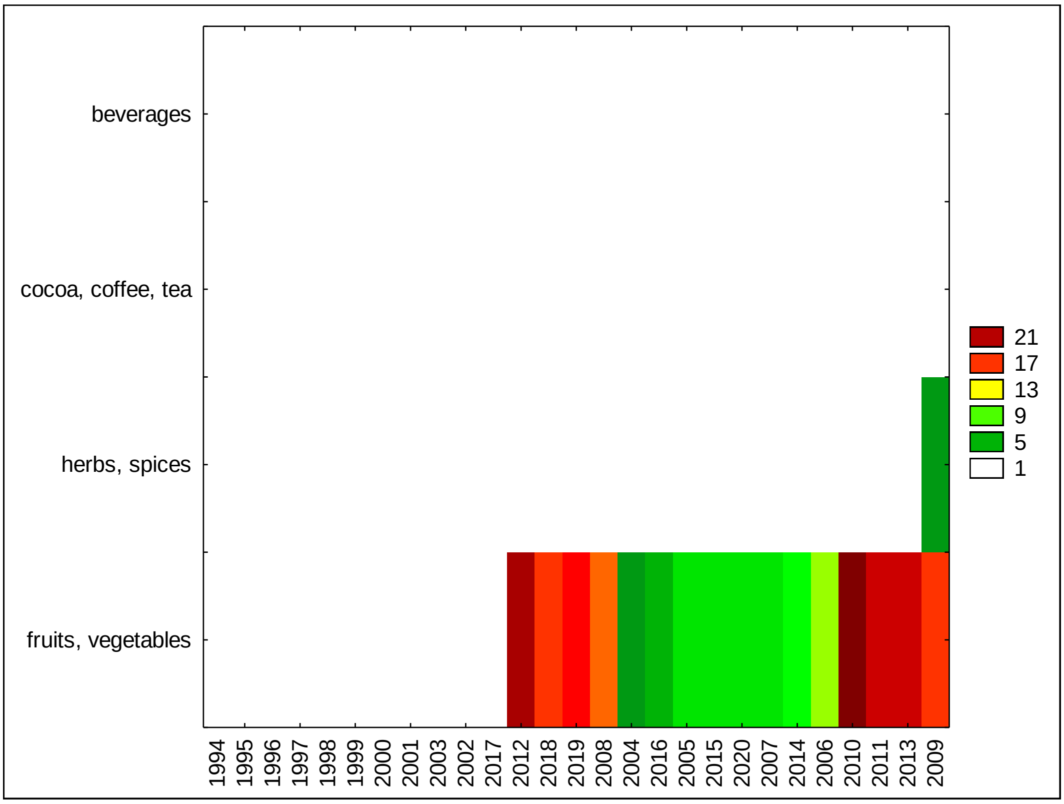

Figure S15b. Results of two-way joining cluster analysis for omethoate (product category)

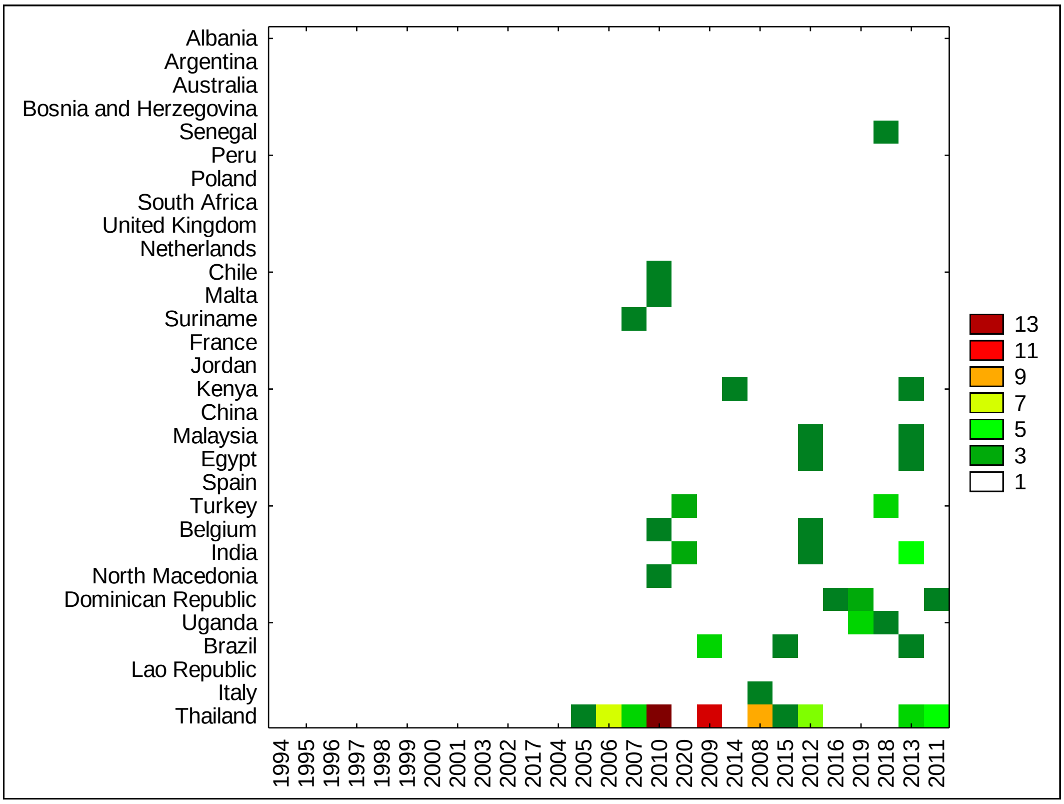

Figure S15c. Results of two-way joining cluster analysis for omethoate (origin country)

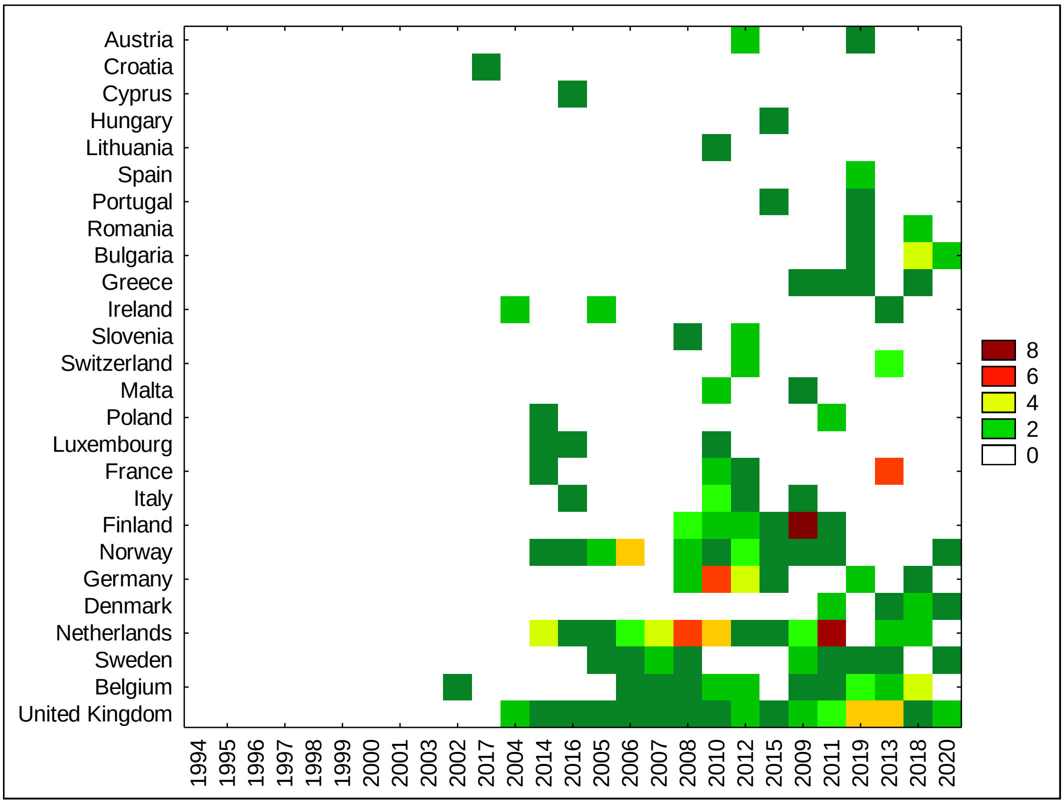

Figure S15d. Results of two-way joining cluster analysis for omethoate (notifying country)

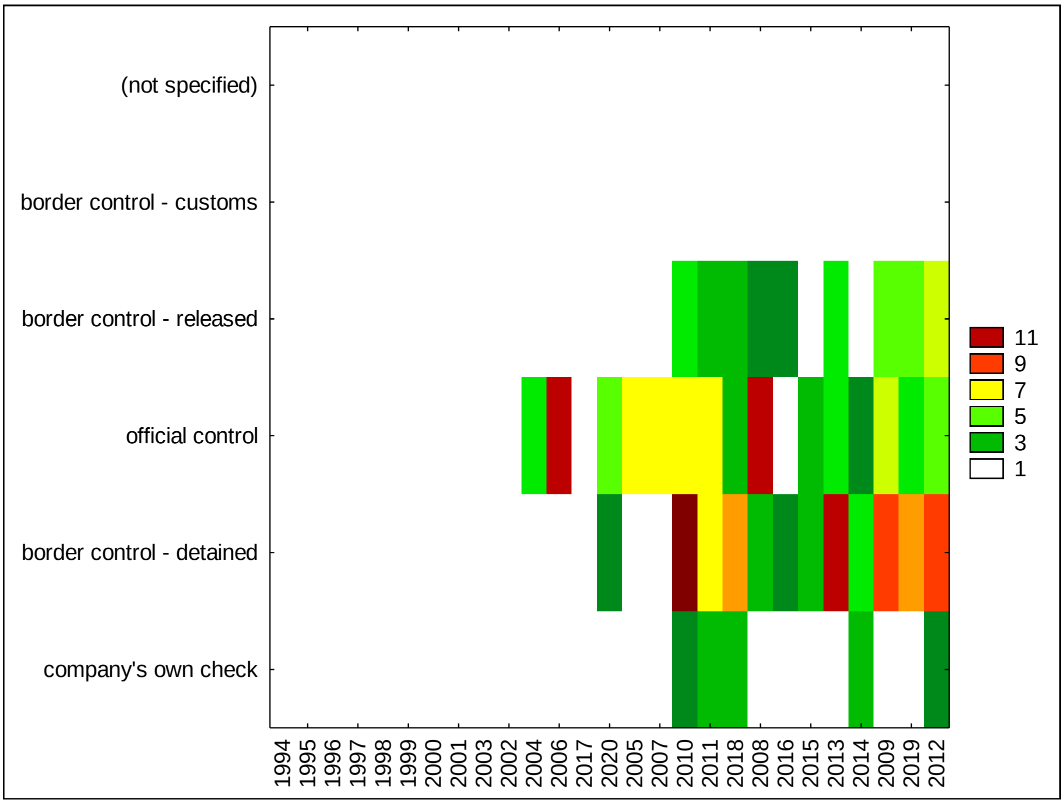

Figure S15e. Results of two-way joining cluster analysis for omethoate (notification basis)

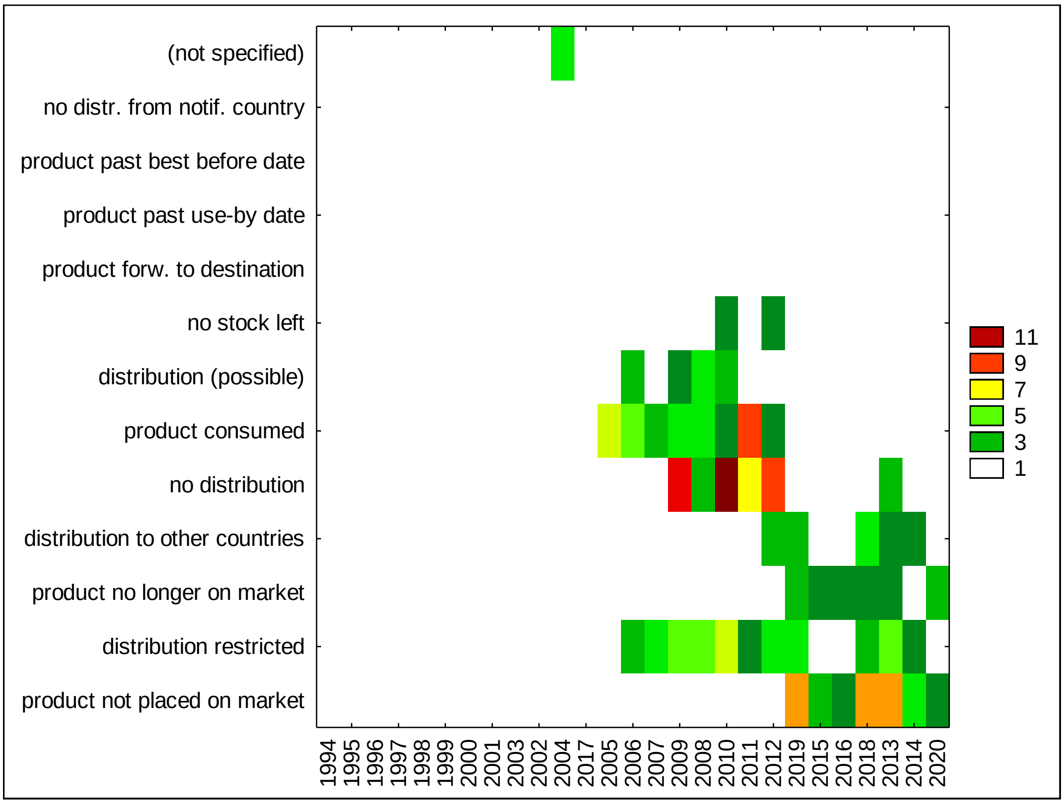

Figure S15f. Results of two-way joining cluster analysis for omethoate (distribution status)

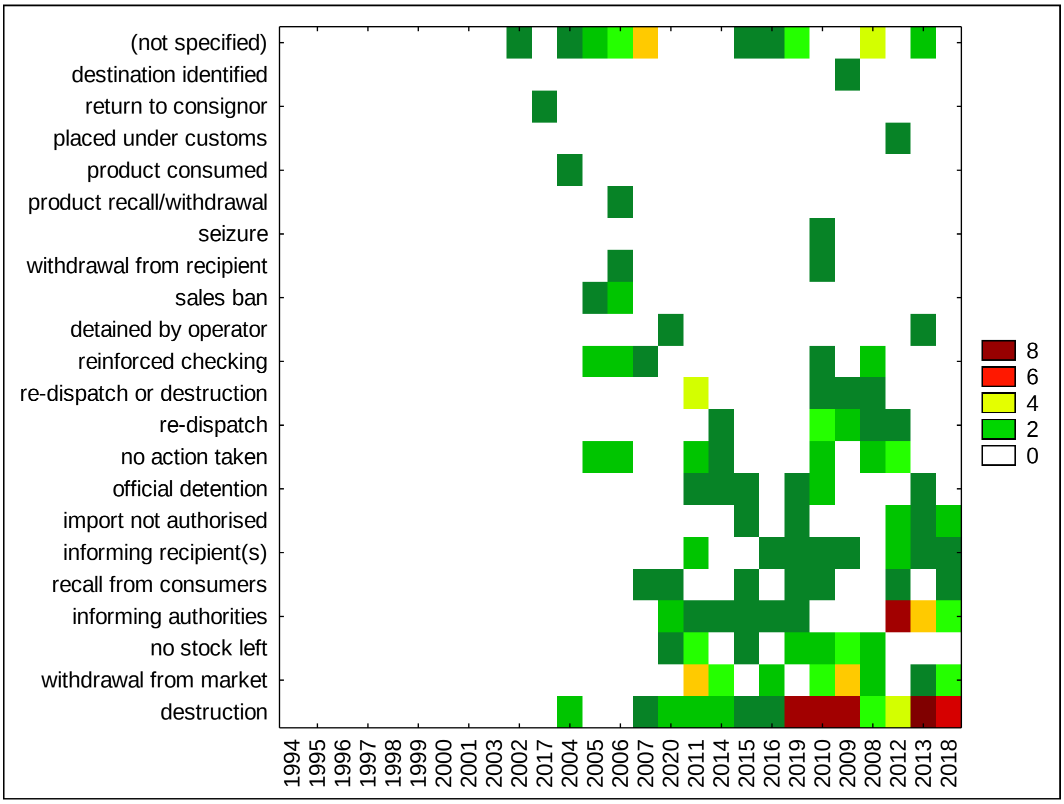

Figure S15g. Results of two-way joining cluster analysis for omethoate (action taken)

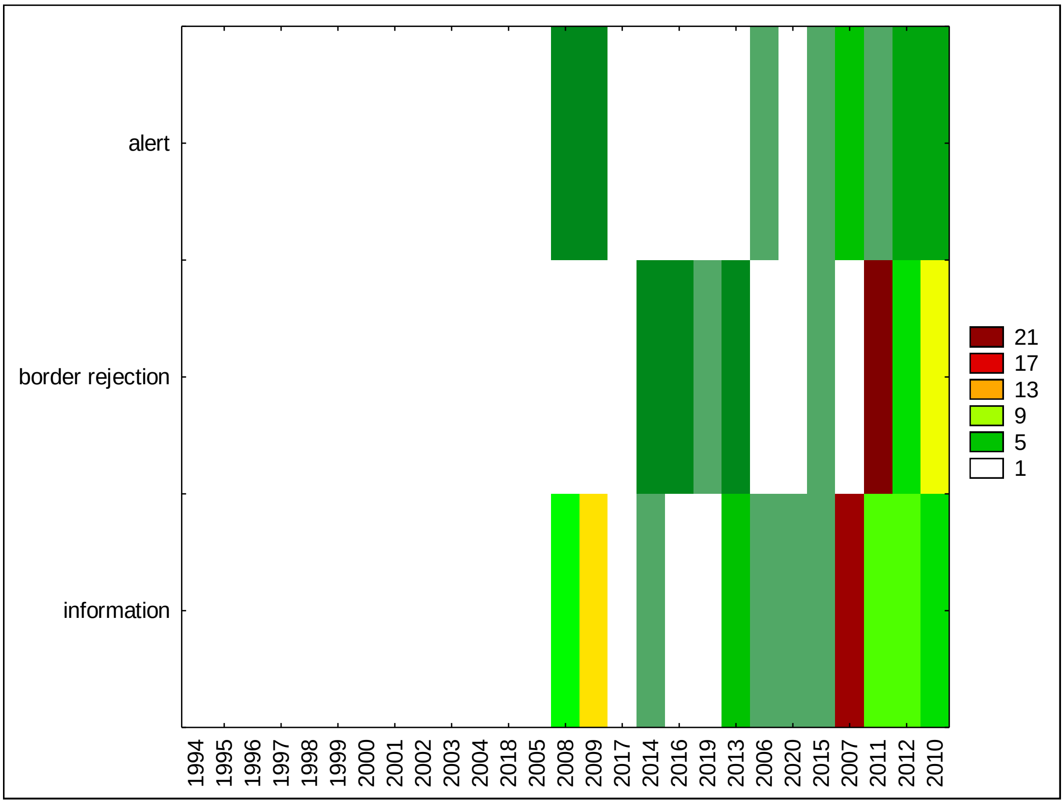

Figure S16a. Results of two-way joining cluster analysis for oxamyl (notification type)

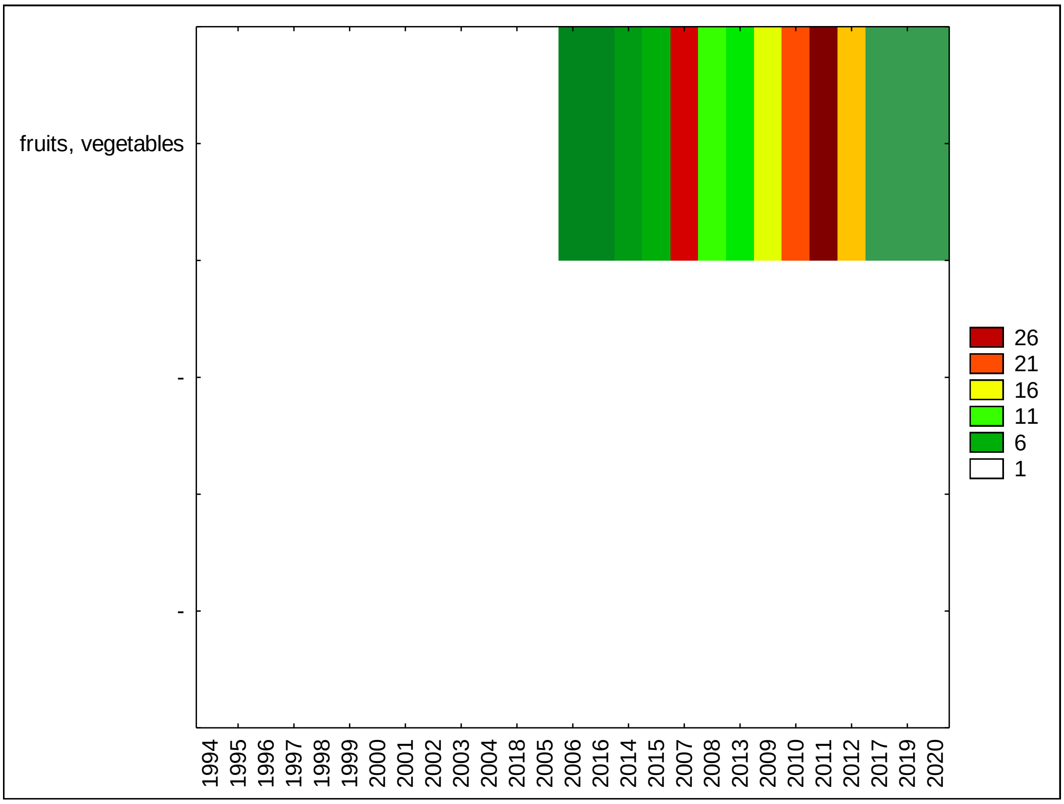

Figure S16b. Results of two-way joining cluster analysis for oxamyl (product category)

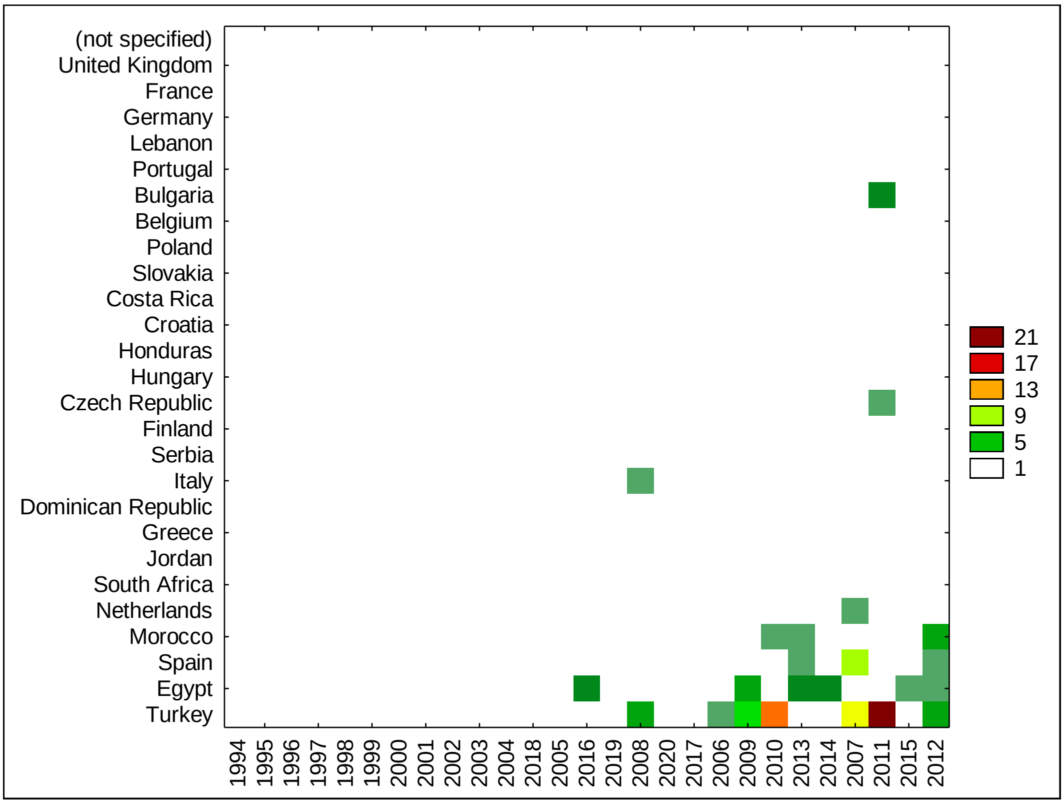

Figure S16c. Results of two-way joining cluster analysis for oxamyl (origin country)

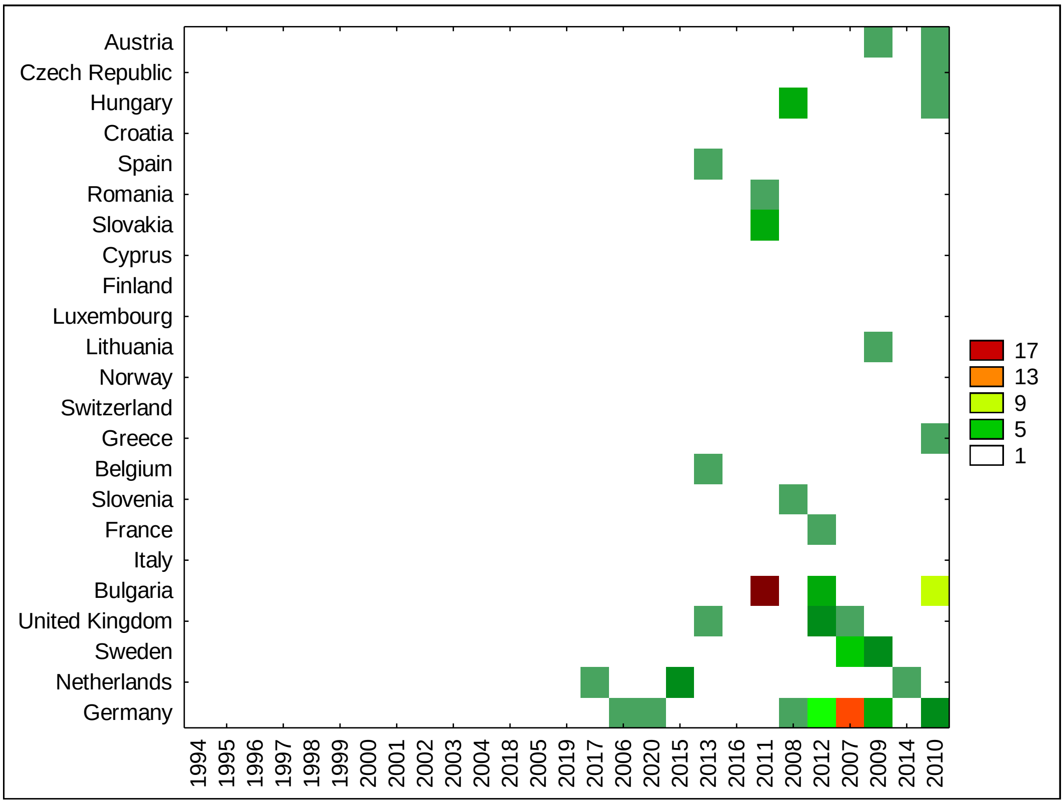

Figure S16d. Results of two-way joining cluster analysis for oxamyl (notifying country)

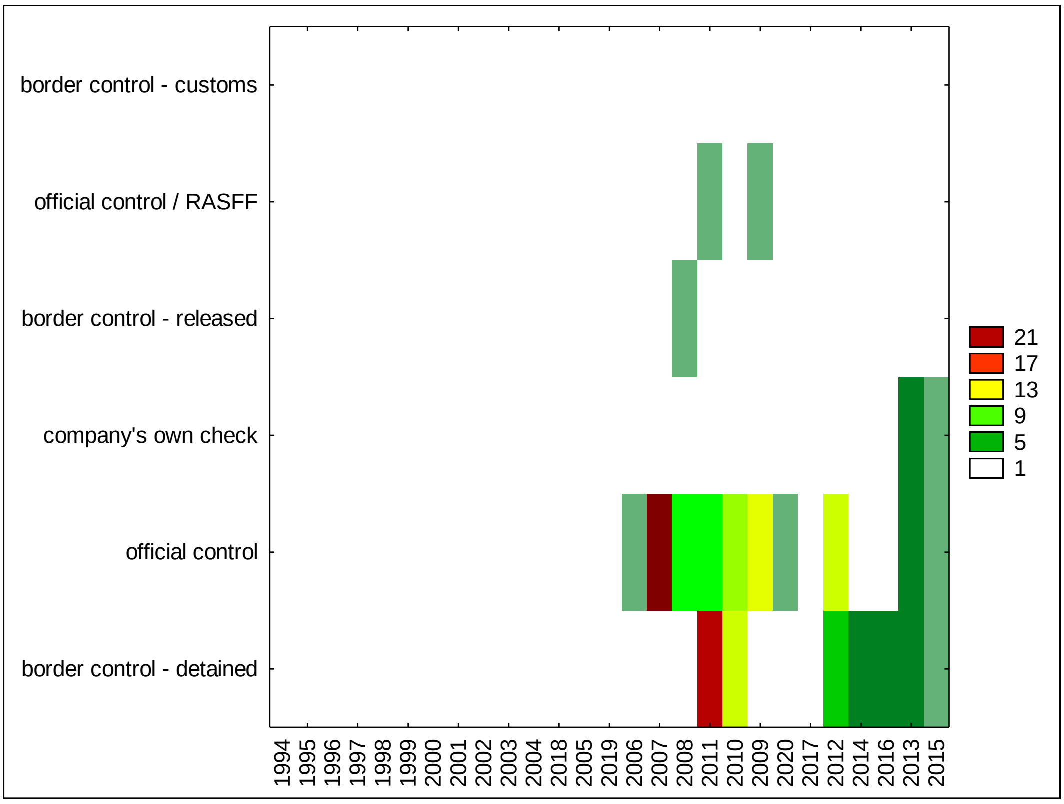

Figure S16e. Results of two-way joining cluster analysis for oxamyl (notification basis)

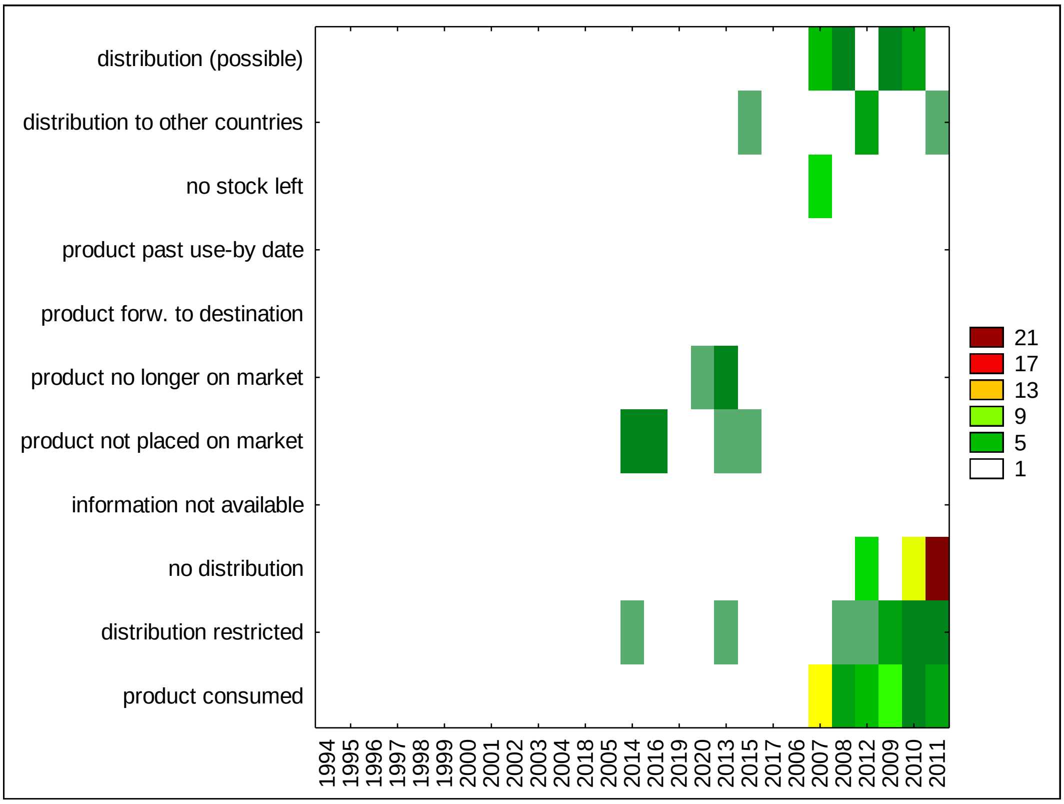

Figure S16f. Results of two-way joining cluster analysis for oxamyl (distribution status)

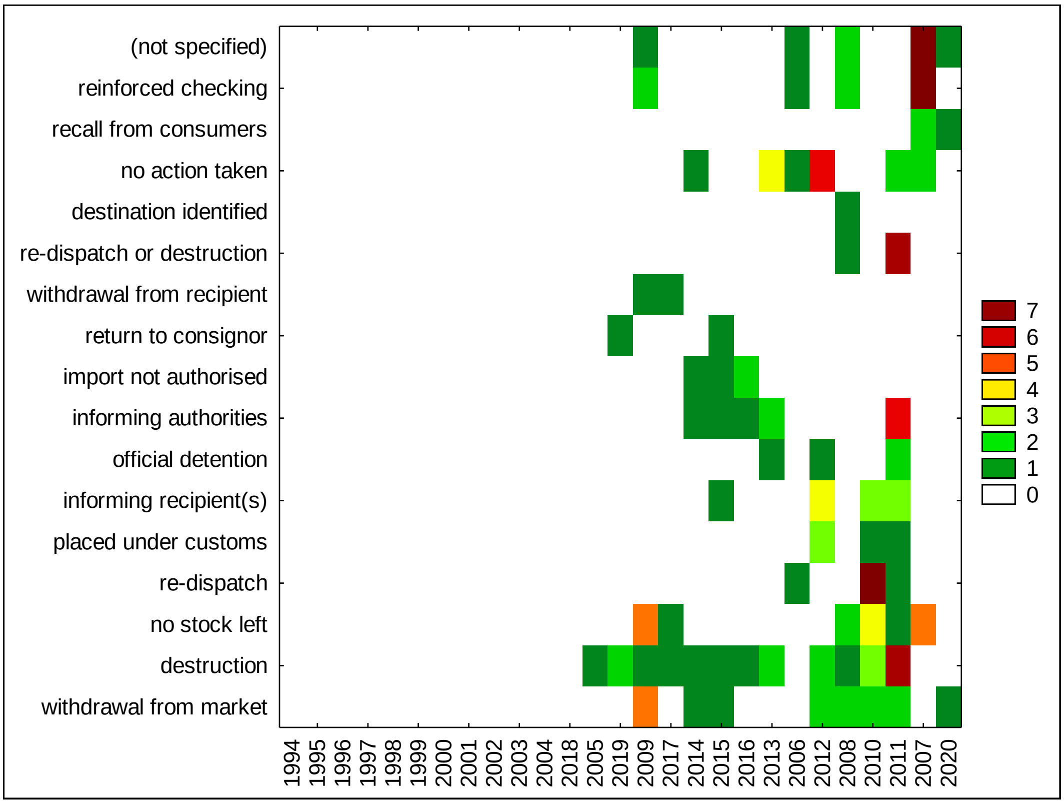

Figure S16g. Results of two-way joining cluster analysis for oxamyl (action taken)

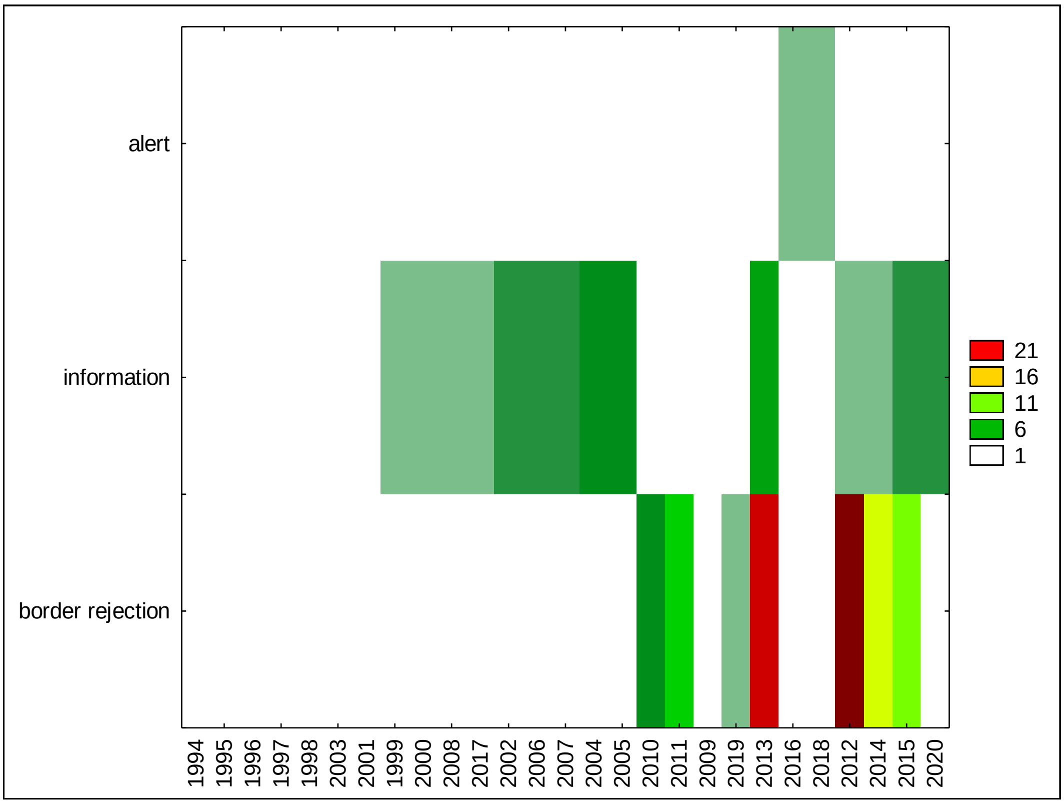

Figure S17a. Results of two-way joining cluster analysis for profenofos (notification type)

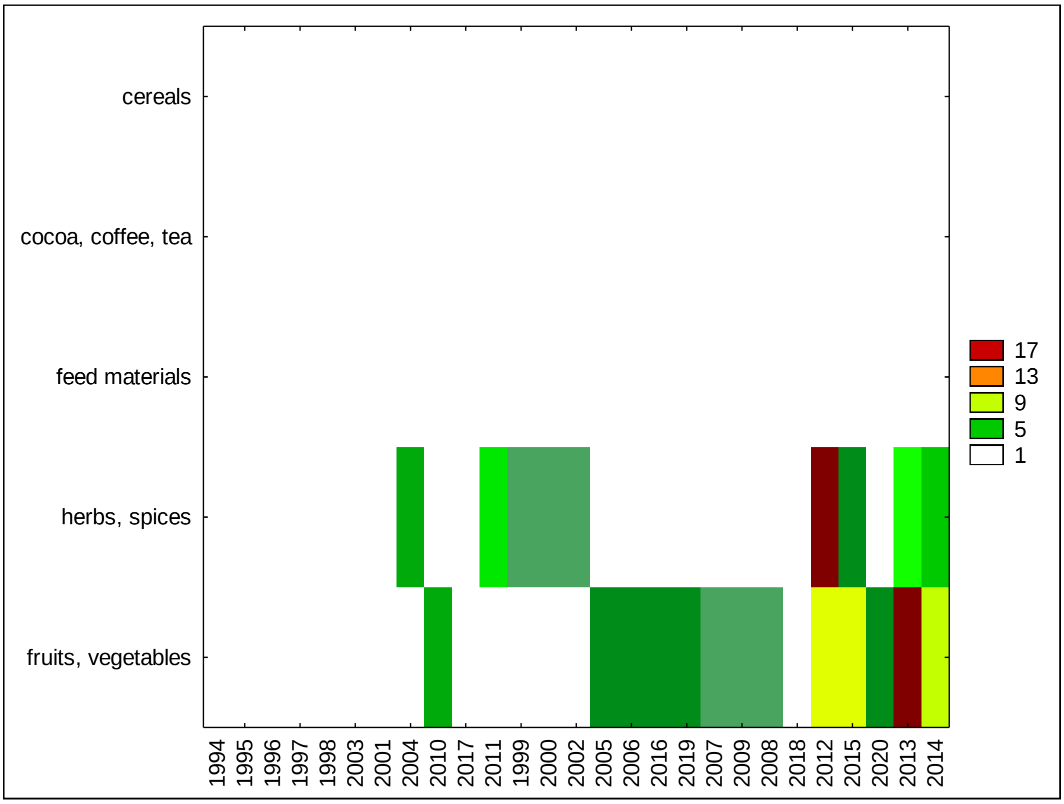

Figure S17b. Results of two-way joining cluster analysis for profenofos (product category)

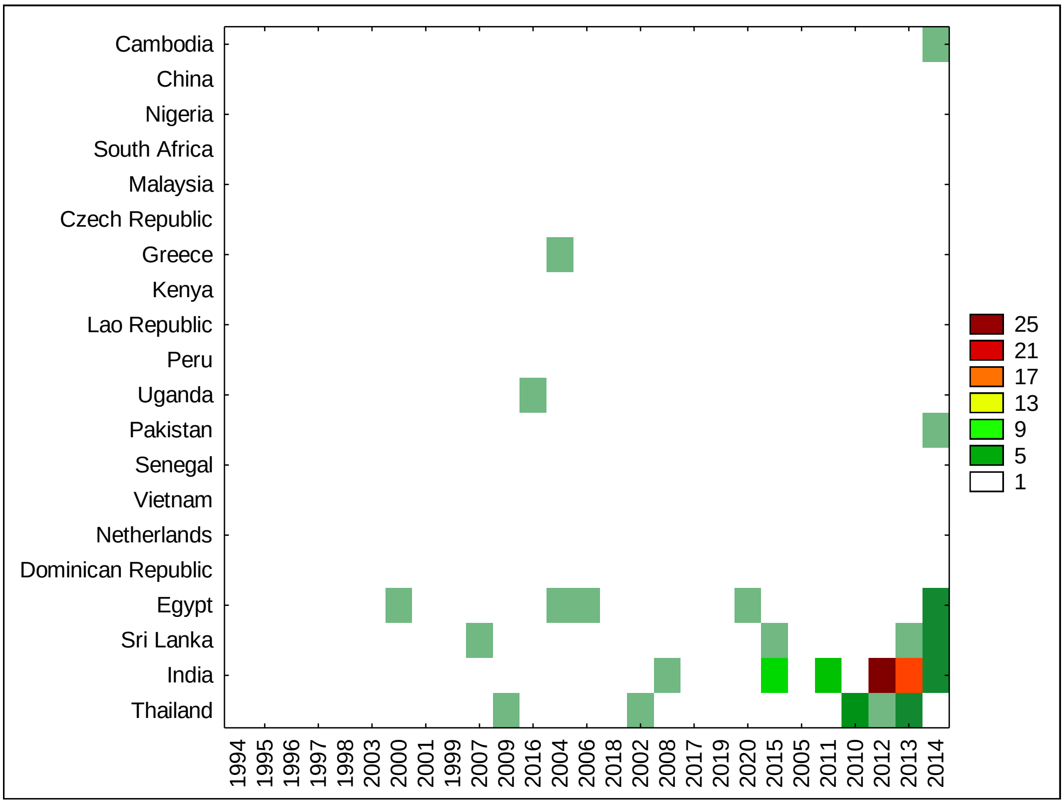

Figure S17c. Results of two-way joining cluster analysis for profenofos (origin country)

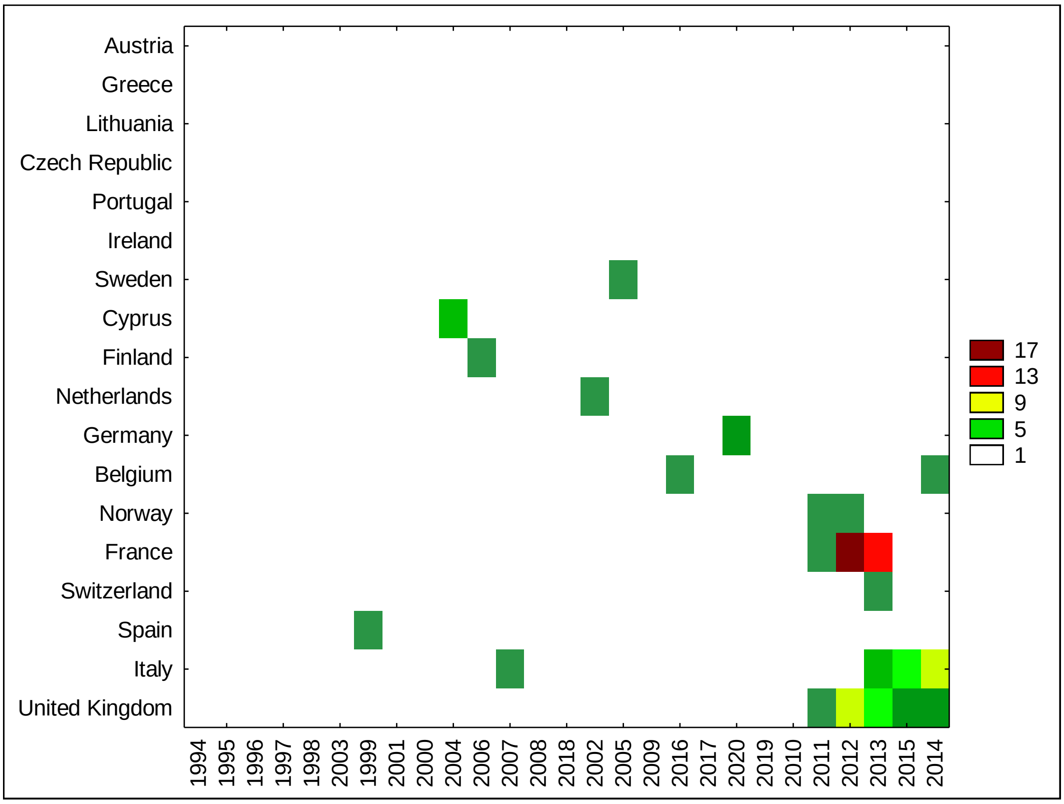

Figure S17d. Results of two-way joining cluster analysis for profenofos (notifying country)

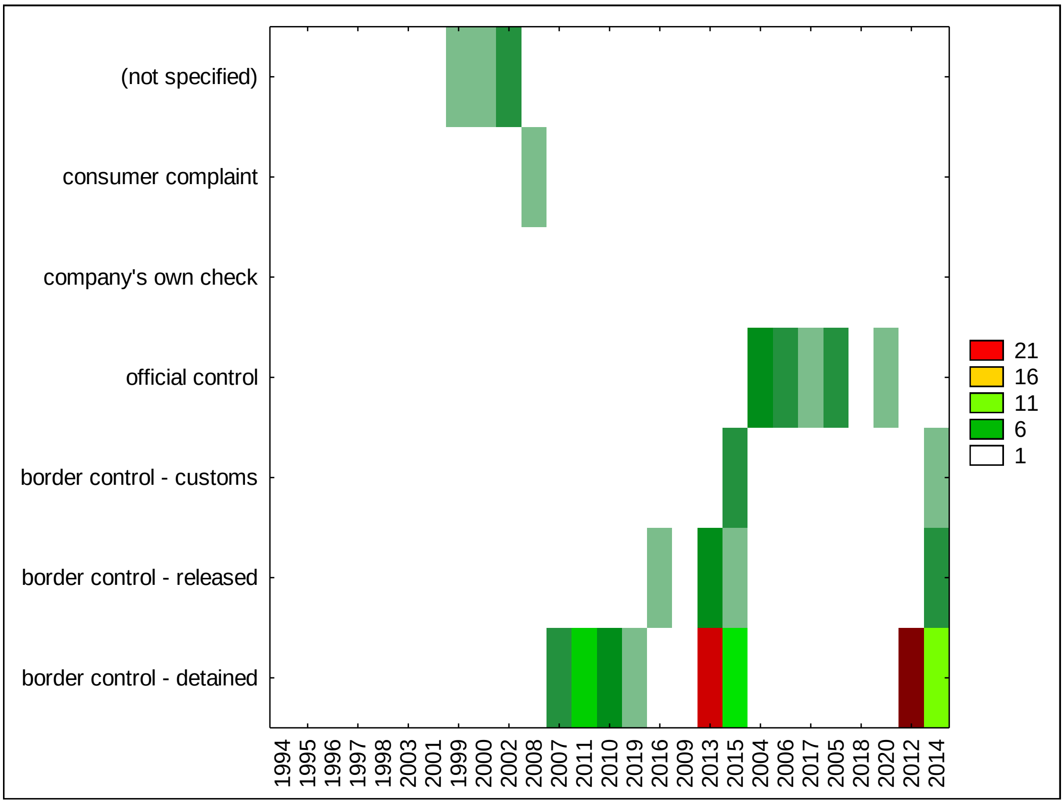

Figure S17e. Results of two-way joining cluster analysis for profenofos (notification basis)

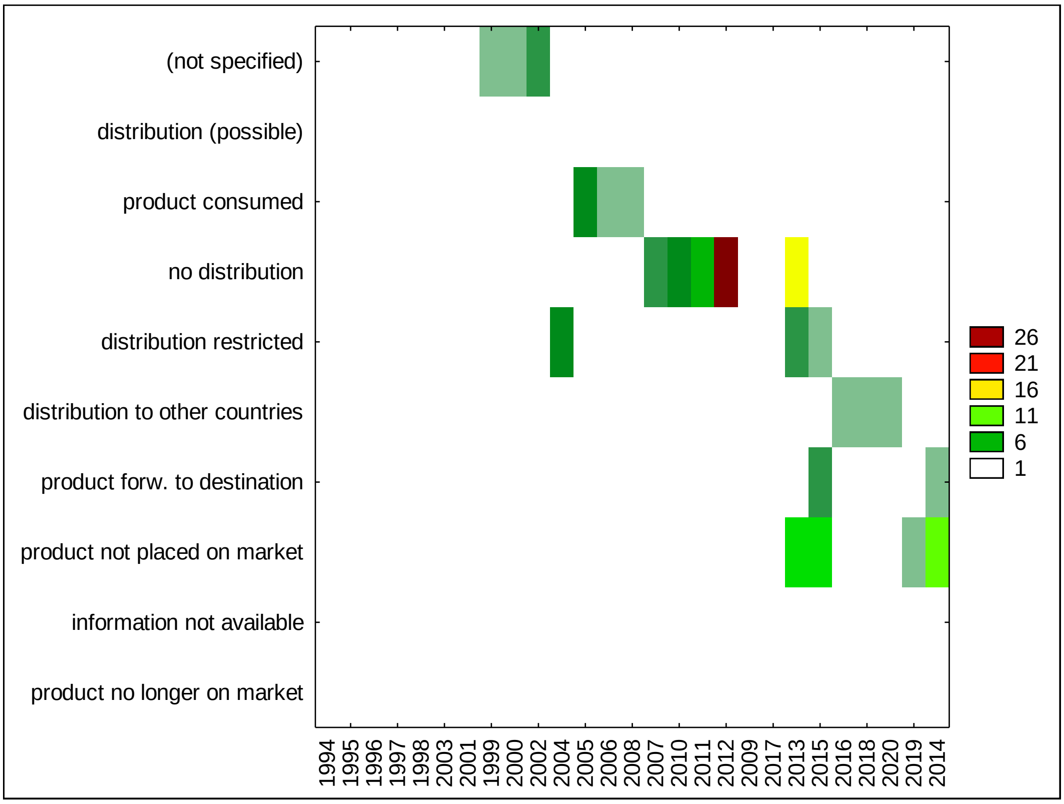

Figure S17f. Results of two-way joining cluster analysis for profenofos (distribution status)

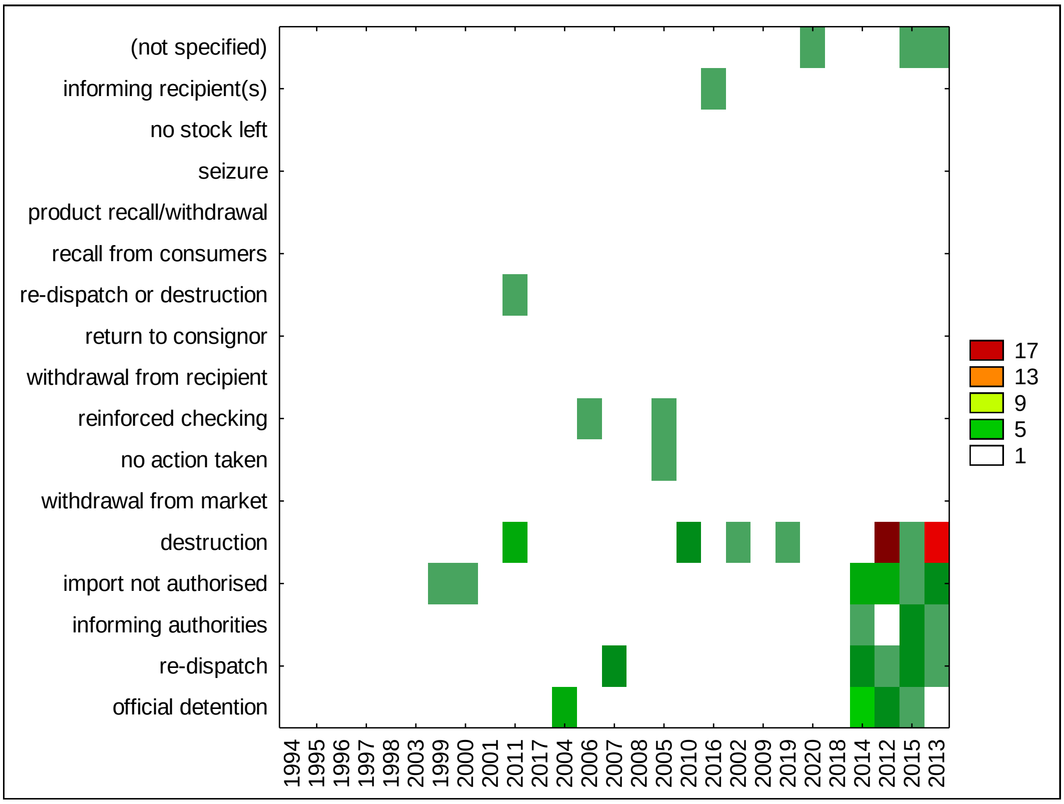

Figure S17g. Results of two-way joining cluster analysis for profenofos (action taken)

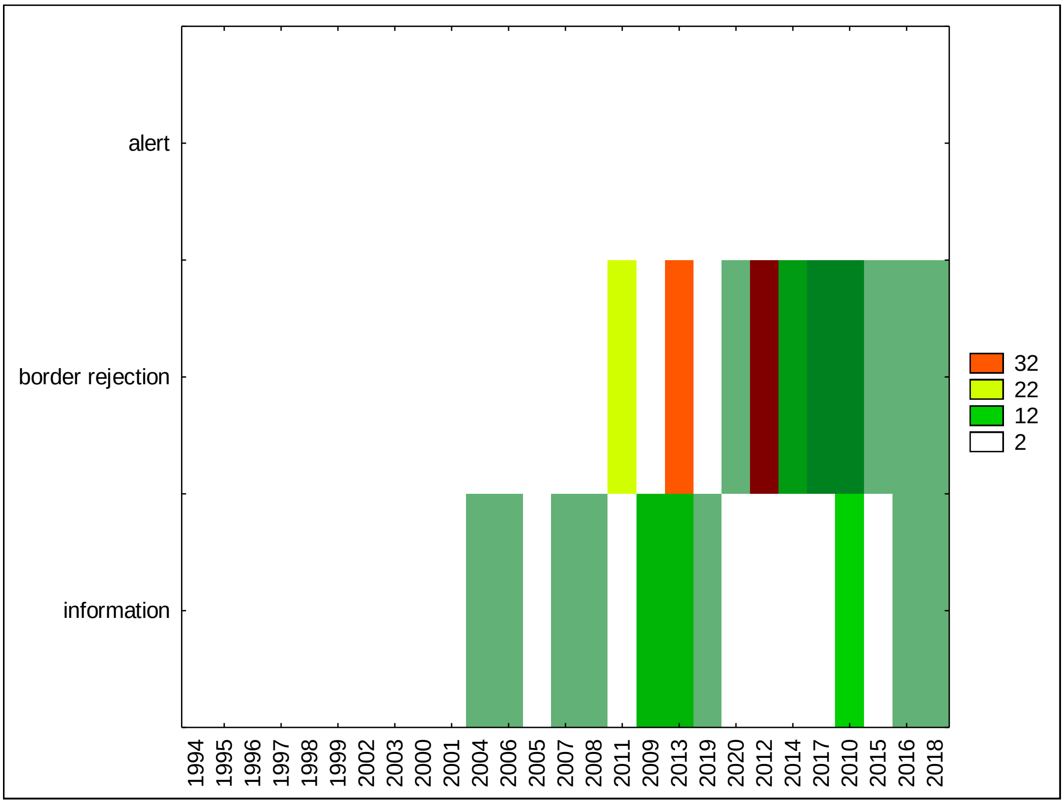

Figure S18a. Results of two-way joining cluster analysis for triazophos (notification type)

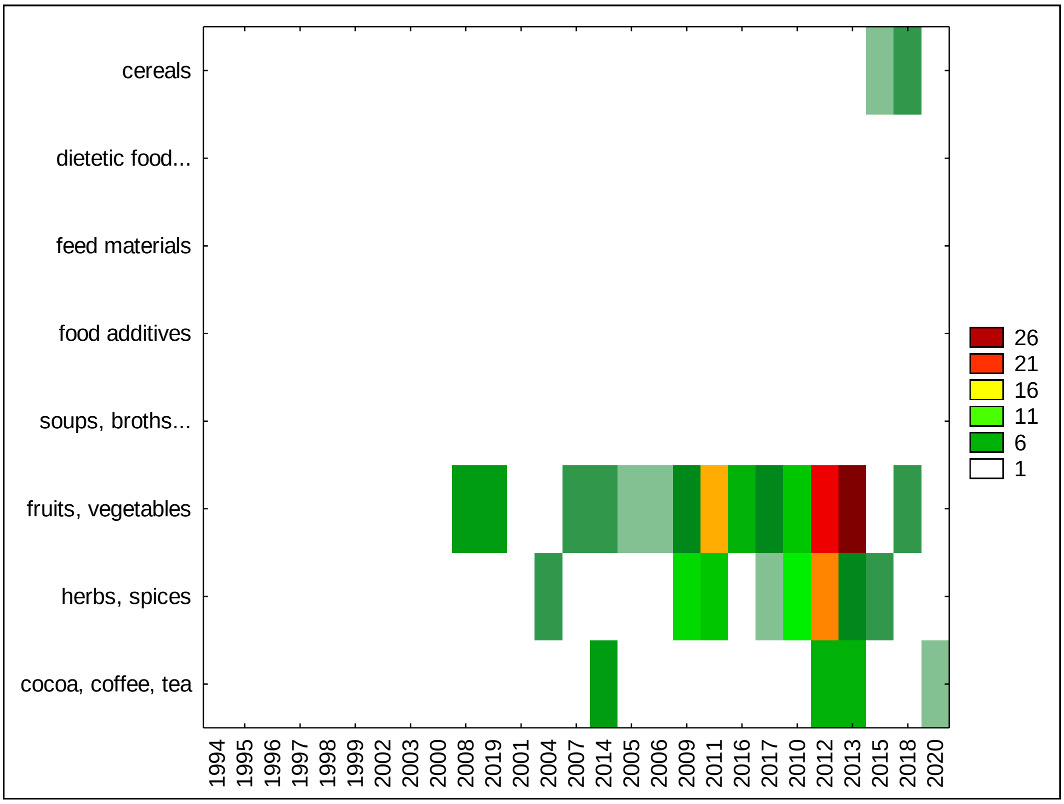

Figure S18b. Results of two-way joining cluster analysis for triazophos (product category)

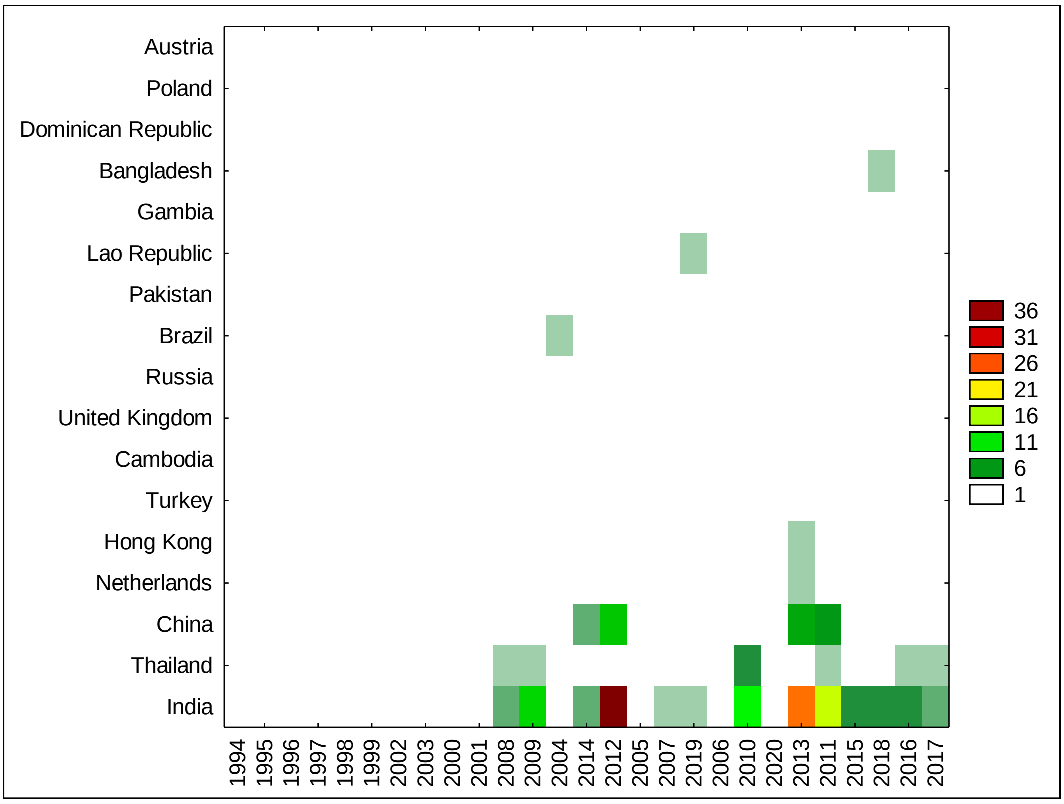

Figure S18c. Results of two-way joining cluster analysis for triazophos (origin country)

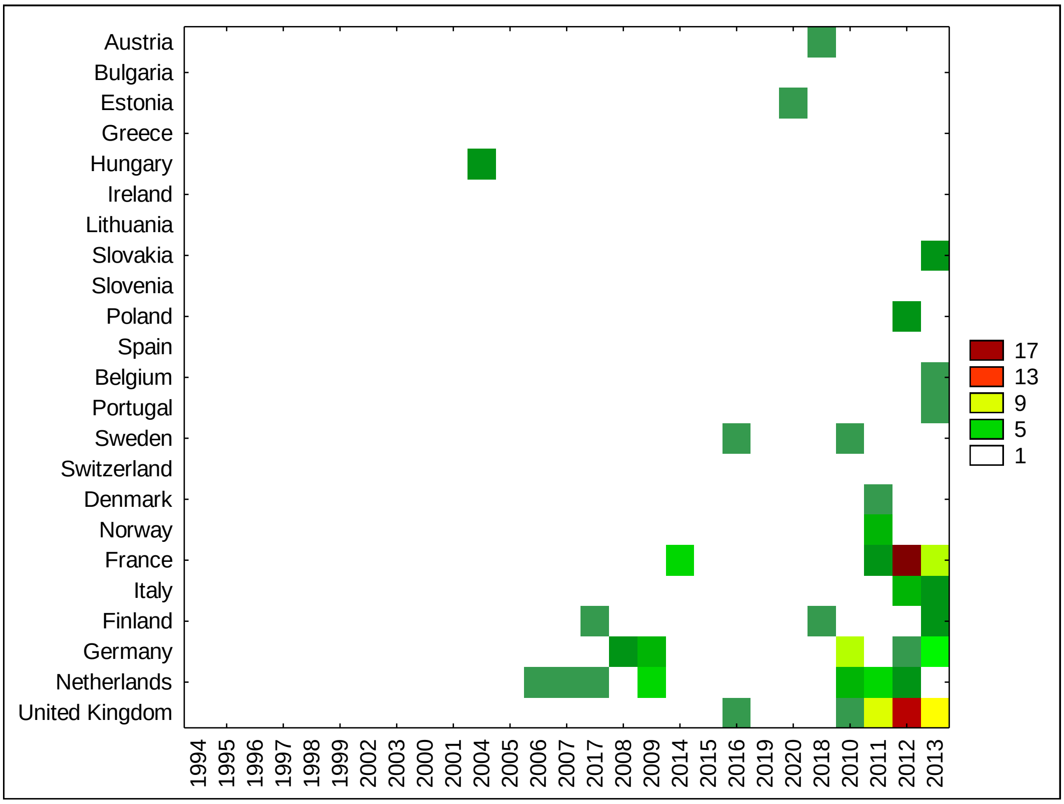

Figure S18d. Results of two-way joining cluster analysis for triazophos (notifying country)

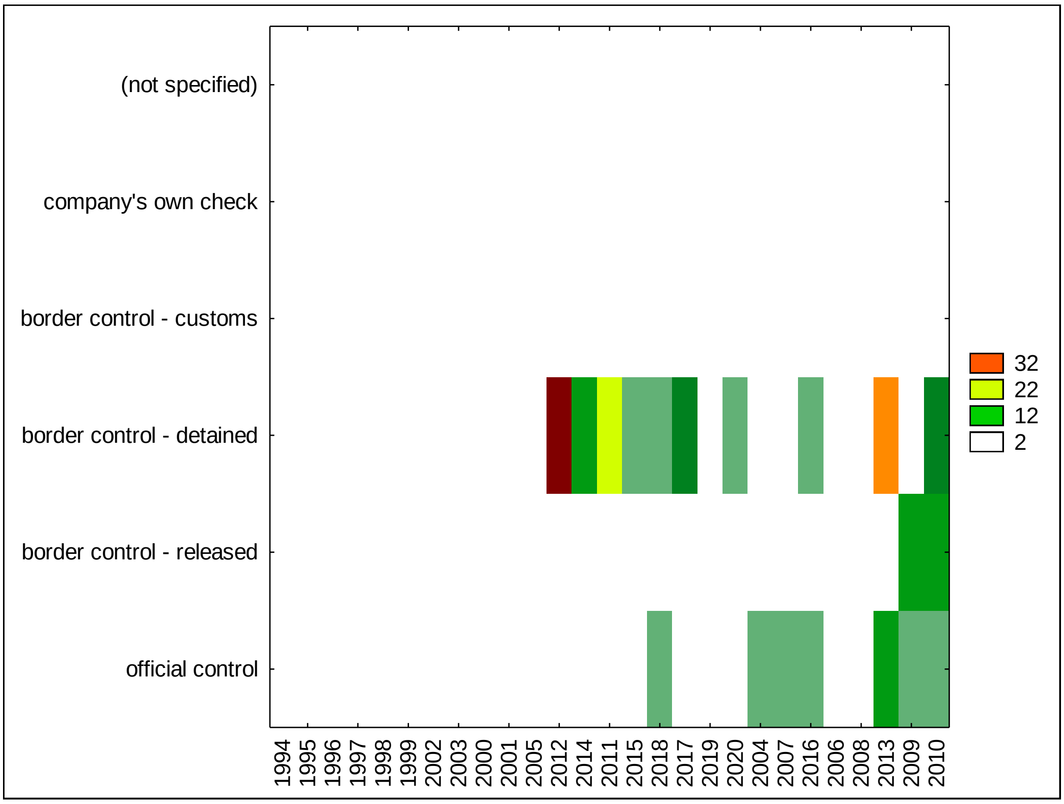

Figure S18e. Results of two-way joining cluster analysis for triazophos (notification basis)

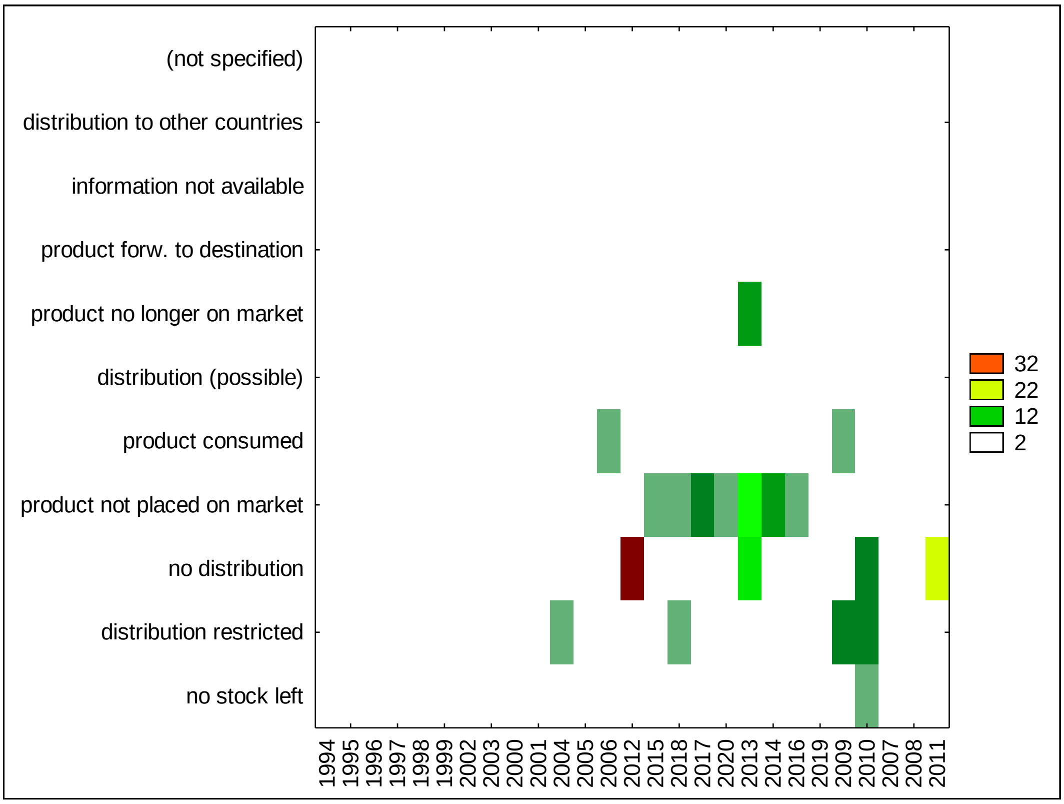

Figure S18f. Results of two-way joining cluster analysis for triazophos (distribution status)

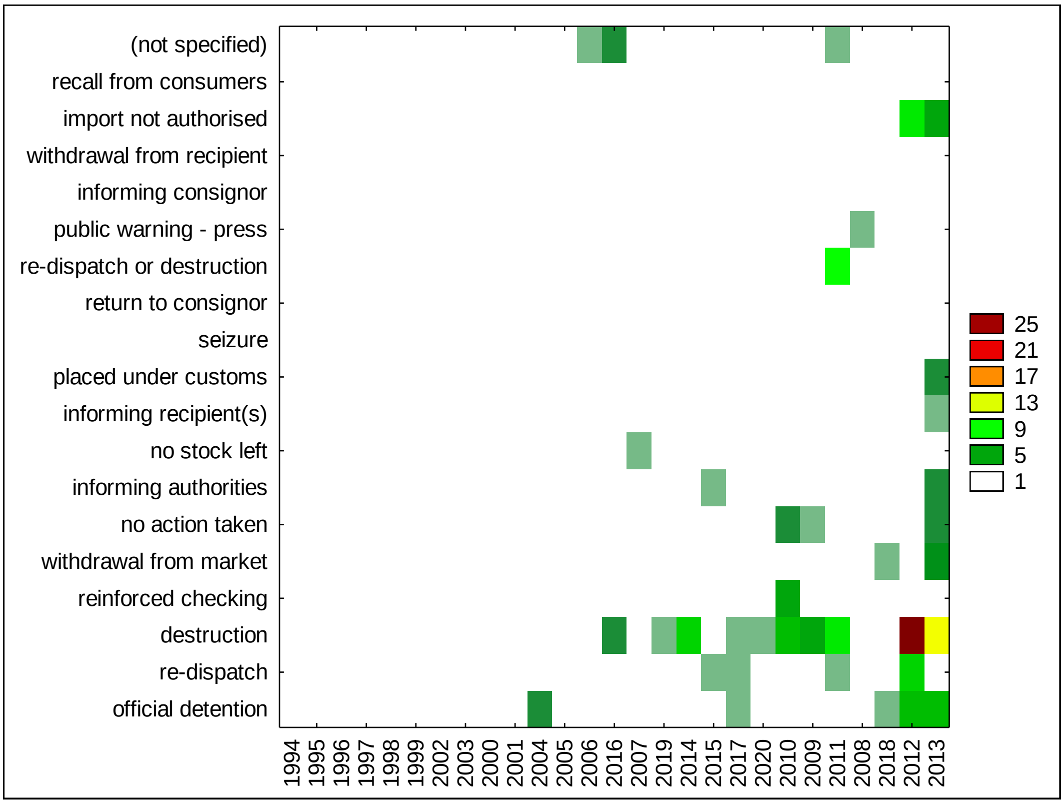

Figure S18g. Results of two-way joining cluster analysis for triazophos (action taken)
